# Preference of Small Molecules for Local Minimum Conformations when Binding to Proteins

**DOI:** 10.1371/journal.pone.0000820

**Published:** 2007-09-05

**Authors:** Qi Wang, Yuan-Ping Pang

**Affiliations:** Computer-Aided Molecular Design Laboratory, Mayo Clinic, Rochester, Minnesota, United States of America; The Scripps Research Institute, United States of America

## Abstract

It is well known that small molecules (ligands) do not necessarily adopt their lowest potential energy conformations when binding to proteins. Analyses of protein-bound ligand crystal structures have reportedly shown that many of them do not even adopt the conformations at local minima of their potential energy surfaces (local minimum conformations). The results of these analyses raise a concern regarding the validity of virtual screening methods that use ligands in local minimum conformations. Here we report a normal-mode-analysis (NMA) study of 100 crystal structures of protein-bound ligands. Our data show that the energy minimization of a ligand alone does not automatically stop at a local minimum conformation if the minimum of the potential energy surface is shallow, thus leading to the folding of the ligand. Furthermore, our data show that all 100 ligand conformations in their protein-bound ligand crystal structures are nearly identical to their local minimum conformations obtained from NMA-monitored energy minimization, suggesting that ligands prefer to adopt local minimum conformations when binding to proteins. These results both support virtual screening methods that use ligands in local minimum conformations and caution about possible adverse effect of excessive energy minimization when generating a database of ligand conformations for virtual screening.

## Introduction

Molecular complexation in biology is best described by the conformational induction theory [1]—namely, a ligand (e.g., a small molecule) binds initially to a less compatible conformation of a receptor (e.g., a protein) and then adjusts its conformation to induce the most compatible conformation of the receptor. The conformation induction theory is, however, not ideal for computationally addressing the conformational flexibility of both ligand and receptor in docking studies, because computing the mutually dependent conformational changes of both partners on the fly is time-consuming and unsuitable for parallel computing. Alternatively, the conformation selection theory describes that both ligand and receptor select their *preformed* conformations that are most compatible with one another to effect binding by shifting two equilibriums progressively from less compatible to most compatible conformations for both partners, where the preformed and most compatible conformations are conformations at local minima of their potential energy surfaces (local minimum conformations) [2]–[5]. When the most compatible conformations of both partners are most prevalent, the conformation selection theory becomes the lock–key theory [1]. The conformation selection theory is ideal to computationally account for molecular flexibility in docking, because it can convert a ligand–receptor association best described by the conformational induction theory to a series of associations each of which can be described by the lock-key theory [6]. The conformation selection theory thereby affords parallel computing and enables a docking study to be performed on thousands of IBM Blue Gene processors with high processor utilization [6]–[8].

It is well known that ligands do not necessarily adopt their lowest potential energy conformations when binding to their protein targets [9]–[11]. Analyses of crystal structures of protein-bound ligands have reportedly shown, however, that many of them do not adopt their local minimum conformations [12], [13]. In particular, a study of 150 protein-bound ligand crystal structures showed that more than 60% of them do not adopt local minimum conformations [13]. The results of these analyses raise a concern regarding the validity of virtual screening methods that use ensembles of local minimum conformations of both ligand and receptor to address molecular flexibility according to the conformation selection theory [6]. To address this concern by investigating why many ligands reportedly do not adopt local minimum conformations when binding to proteins [13], we carried out a normal-mode-analysis (NMA) study, that used analytic means to analyze harmonic potential wells and classify possible deformations of these ligands according to their energetic costs [14]–[17], using the second-generation AMBER force field [18], [19].

Here we report an NMA study of 100 available protein-bound ligand crystal structures that were studied in reference 13. Our data show that the energy minimization of a ligand alone does not automatically stop at a local minimum conformation. Furthermore, our data show that all 100 ligand conformations in their protein-bound ligand crystal structures are nearly identical to their local minimum conformations that were obtained from the crystal structures and refined by energy minimization whose progress was monitored with NMA. These results both support the virtual screening methods that use local minimum conformations and caution about possible adverse effect of excessive energy minimization on generating databases of ligand conformations for virtual screening.

## Results

### Ligand folding caused by excessive energy minimization

To understand why more than 60% of the 100 available protein-bound ligand crystal structures are reportedly not in their local minimum conformations [13], we carried out an NMA study of these ligands in the absence of their protein partners. This study was pursued because the translational, rotational, and vibrational frequencies of a nonlinear ligand can be used diagnostically to determine how close the ligand conformation is to its local minimum conformation [17]. Before starting NMA, it was necessary to perform a few steps of energy minimization on a ligand conformation, taken from the ligand-protein complex crystal structure, in the absence of its protein partner to “adapt” the ligand to the force field used by the NMA as well as to reduce the gradient of the ligand potential energy to zero [15]. This preparation was necessary because ligand conformations in crystal structures are refined to best fit the electron density map. Such conformations can be energetically unstable or “strained” if their potential energies are evaluated in the absence of proteins using a force field that is different from the one used by the NMA.

By performing 10^6^ steps of energy minimization on one of the 100 ligands [Protein Data Bank (PDB) code: 1QL9] in the absence of its protein partner, we obtained a conformation that was a local minimum conformation (L1) according to NMA. This energy minimization procedure and the ones described hereafter were carried out using the SANDER module of the AMBER 5 program [20] with the AMBER force field [18], [19]. L1 has a mass-weighted root mean square deviation (mwRMSD) of 1.98 Å relative to the crystal structure conformation, indicating that the ligand conformation in the crystal structure was not in its local minimum conformation. This indication was based on a criterion that two conformations are different if their mwRMSD is more than 1.00 Å. Interestingly, using a new energy minimization procedure that uses NMA to monitor energy minimization progress as described below, we obtained a different local minimum conformation (L2) with an mwRMSD of 0.31 Å, indicating that the ligand conformation in the crystal structure was in its local minimum conformation.

**Figure 1:**
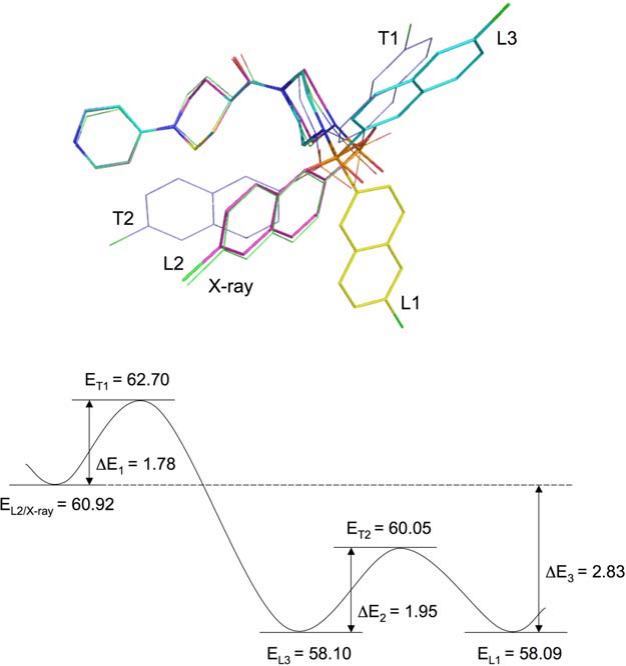
Three local minimum conformations of the ligand taken from crystal structure 1QL9 and two related transition state conformations. Upper panel: local minimum, transition state, and crystal structure conformations of the ligand. The C atom of the crystal structure conformation is green. The C atoms of the local minimum conformations of L1, L2, and L3 are yellow, magenta, and cyan, respectively. The C atoms of the two transition state conformations are blue. The O, N and S atoms are red, blue, and orange, respectively. Lower panel: the potential energy diagram for the exchange between L2 and L1. The unit of the potential energy is kcal/mol.

A further NMA study identified another different local minimum conformation (L3) and two transition state conformations (T1 and T2). These three conformations are related to the exchange between L1 and L2. As shown in Figure 1, L2 changes to T1, then to L3, then to T2, and then to L1; the transition from L2 to L1 markedly changes the ligand conformation found in the crystal structure yet the highest energy barrier for this transition is only 1.95 kcal/mol. Within the context of the AMBER force field (because local minimum conformations are specific to the force field used by NMA), these results show that the energy minimization of a ligand conformation alone does not automatically stop at a local minimum conformation if the minimum of the potential energy surface is shallow. The ligand can progress through multiple shallow, local minima if the progress of the energy minimization is not monitored with NMA.

After energy minimization of the 100 ligands in the absence of their protein partners using 10^6^ steps of energy minimization, we found that all of the energy-minimized conformations were in their local minimum conformations according to NMA, but 42 and 72% of them had mwRMSDs greater than 1.00 and 0.50 Å, respectively, relative to their crystal structure conformations. These results suggest that, similar to the ligand in crystal structure 1QL9, other ligands may have multiple shallow minima on their potential energy surfaces. Within the context of the AMBER force field, these results also caution that excessive energy minimization can fold or partially fold a ligand in its free state. Such folding can mislead the determination of whether the crystal structure conformations are in their local minimal conformations, although the ability to progress through multiple shallow, local minima is desirable in searching for the global minimum conformation or lower local minimum conformations.

### Normal-mode-analysis-monitored energy minimization procedure

**Figure 2:**
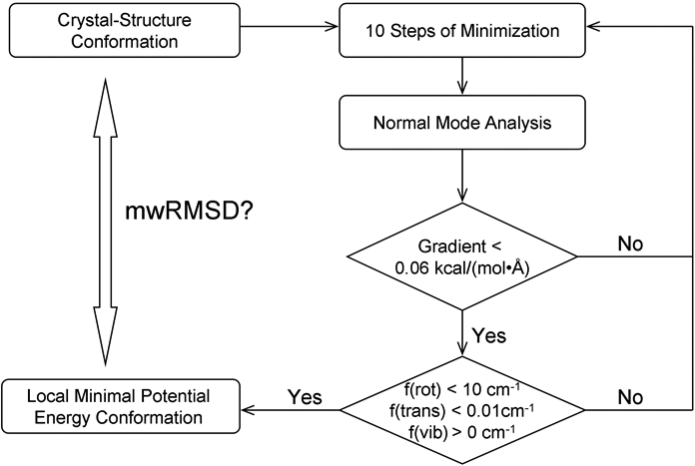
Flowchart of the normal-mode-analysis–monitored energy minimization procedure. mwRMSD stands for mass-weighted root mean square deviation.

To stop energy minimization of a ligand in its free state when it reaches at a local minimum conformation—thus avoiding ligand folding—we devised an NMA-monitored energy minimization (NEM) procedure to investigate whether the 100 ligands under study were in, near, or far from local minimum conformations. As shown in Figure 2, this procedure begins with 10 steps of energy minimization on a ligand (in this study the ligand conformation was taken from its protein–bound ligand crystal structure). The energy-minimized ligand conformation is then subject to NMA to check whether the ligand is in its local minimum conformation. The 10-step energy minimization uses a gradient cut-off of 10^−7^ kcal/(mol•Å) and is repeated until the NMA shows that the ligand is in a local minimum conformation. After each 10-step energy minimization, the gradient of the ligand potential energy is checked first. If the gradient is >0.06 kcal/(mol•Å), NMA is aborted, and the ligand is considered not to be in its local minimum conformation. If the gradient is ≤0.06 kcal/(mol•Å), NMA is performed, and the magnitudes of three translational and three rotational frequencies are checked. If the magnitudes of all translational frequencies are <0.01 cm^−1^ and the magnitudes of all rotational frequencies are <10 cm^−1^, all vibrational frequencies are checked; otherwise, the analysis of vibrational frequencies is aborted and the ligand is considered not to be in its local minimum conformation. If all the vibrational frequencies are positive, the ligand is considered to be in its local minimum conformation [16], [17]. The cut-offs for the gradient and for the translational and rotational frequencies are obtained from reference 16 and based on the fact that geometry cannot be optimized to a gradient of exact zero because of numeric truncations [16]. The NEM procedure is automated by a Perl script shown in Figure S1.

### Preference for local minimum conformations

**Figure 3:**
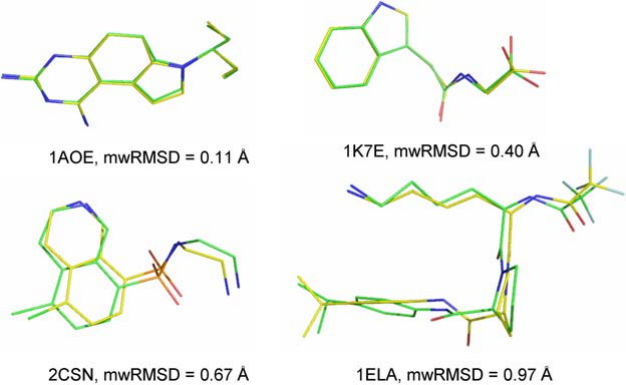
Graphic representation of mass-weighted root mean square deviations (mwRMSDs) of 0.11–0.97 Å between crystal structure conformations and their corresponding local minimum conformations obtained by using normal mode analysis. The C atoms of the crystal structure conformation and the local minimum conformation are green and yellow, respectively. The O, N, F and S atoms are red, blue, grey, and orange, respectively.

For each of the 100 ligands under study, we obtained a local minimum conformation with an mwRMSD of <1.00 Å relative to the ligand conformation in the crystal structure (Tables 1 and S1) when using the NEM procedure, the conjugate gradient minimization method, and a gradient cut-off of 0.06 kcal/(mol•Å). In contrast to the observation that 28 of the 100 ligands have local minimum conformations with mwRMSDs of ≤0.50 Å relative to their crystal structure conformations when using the conventional energy minimization method as described above, 69 of the 100 ligands have local minimum conformations with mwRMSDs of ≤0.50 Å when using the NEM procedure, the conjugate gradient minimization method, and a gradient cut-off of 0.06 kcal/(mol•Å) (Tables 1 and S1). The same result was obtained when using the NEM procedure, the steepest descent minimization method, and a gradient cut-off of 0.06 kcal/(mol•Å) (Table S2). In addition, 78 of the 100 ligands have mwRMSDs of ≤1.00 Å between the ligand conformations in their crystal structures and their corresponding local minimum conformations when using the NEM procedure, the conjugate gradient minimization method, and a smaller gradient cut-off of 10^−2^ kcal/(mol•Å) that adds extra steps of energy minimization (Table S3). Figure 3 shows the closeness between the ligand conformations in the crystal structures and their corresponding local minimum conformations obtained using the NEM procedure (mwRMSDs of 0.11–0.97 Å). Within the context of the AMBER force field, these results indicate that all 100 ligand conformations in the crystal structures of their protein complexes are nearly identical to their local minimum conformations, demonstrating the preference of these ligands for local minimum conformations when binding to proteins.

**Table 1:** Mass-Weighted Root Mean Square Deviations (mwRMSDs) of All Ligand Atoms between the Crystal Structure Conformation and the Local Minimum Conformation for the 100 Protein-Bound Ligands.

PDB^1 ^code	mwRMSD (Å)		PDB code	mwRMSD (Å)		PDB code	mwRMSD (Å)		PDB code	mwRMSD (Å)	
	M1^2^	M2^3^		M1	M2		M1	M2		M1	M2
1AOE	0.11	0.21	3STD	0.29	0.28	1YDT	0.41	0.86	1F4E	0.56	0.57
1H9U	0.13	0.29	1THL	0.29	0.67	1DIB	0.41	0.47	1NHU	0.57	1.03
1EXA	0.13	0.81	1BR6	0.30	0.67	1MQ5	0.41	0.66	1IF7	0.58	0.88
1M48	0.14	0.49	1PPC	0.30	1.65	1PPH	0.42	1.65	1FJS	0.58	0.90
4STD	0.14	0.25	3ERK	0.31	0.45	1AZM	0.42	0.51	1D6V	0.59	0.89
5STD	0.19	0.68	1QL9	0.31	1.98	1G4O	0.42	0.93	1ATL	0.61	1.03
1FCX	0.20	1.32	3CPA	0.31	0.45	1F0R	0.43	2.28	1NHV	0.61	3.00
2PCP	0.20	0.27	1IY7	0.31	0.36	1UVT	0.43	2.26	1EZQ	0.62	1.07
1JSV	0.22	0.34	1H1P	0.32	0.74	1MMB	0.46	1.08	1GWX	0.62	2.90
3TMN	0.22	0.29	1YDS	0.33	0.43	2CGR	0.46	1.15	1I8Z	0.64	0.72
1QHI	0.23	0.33	1EFY	0.35	0.43	966C	0.47	0.53	1HPV	0.65	1.20
1K1J	0.23	0.61	1ETT	0.35	1.76	1HTF	0.47	1.28	1UVS	0.65	1.12
1FM6	0.23	0.38	1HDQ	0.35	0.39	1LQD	0.47	1.59	2CSN	0.67	0.97
1HFC	0.23	0.38	4DFR	0.36	1.74	1R09	0.47	0.55	1SYN	0.67	2.35
1ETR	0.24	0.55	1FM9	0.36	2.01	1FRB	0.50	1.38	1A42	0.69	1.00
1CET	0.25	0.44	1A4K	0.36	0.46	1K22	0.50	1.18	1BQO	0.74	1.21
1I7Z	0.25	0.43	1MQ6	0.36	1.60	1D3P	0.50	1.18	1F4G	0.76	1.71
1FCZ	0.25	0.36	1AFQ	0.38	0.51	1H1S	0.50	0.86	5TLN	0.77	1.27
1DLR	0.26	0.49	2QWI	0.39	0.51	1KV2	0.50	1.28	1QBU	0.79	1.27
3ERT	0.26	0.46	1CIM	0.39	0.51	1L2S	0.51	1.22	7DFR	0.82	1.33
1KV1	0.27	0.36	1K7E	0.40	0.64	1O86	0.52	0.81	1FKG	0.87	1.29
1QPE	0.28	0.30	1EVE	0.40	0.60	830C	0.53	1.30	1A8T	0.88	1.24
1F0T	0.28	1.81	1K7F	0.40	0.58	13GS	0.54	0.76	1D4P	0.93	1.29
1MNC	0.28	0.39	1L8G	0.40	0.52	1OHR	0.55	1.16	1BNW	0.96	1.25
1YDR	0.28	0.31	7EST	0.41	2.25	1F4F	0.56	1.59	1ELA	0.97	1.21

1 PDB: Protein Data Bank;

2 M1: using the normal-mode-analysis-monitored energy minimization procedure;

3 M2: using 10^6^ steps of energy minimization without using normal mode analysis to monitor the progress of the energy miminization.

## Discussion

### Support for the conformation-selection-theory-based virtual screening

**Figure 4:**
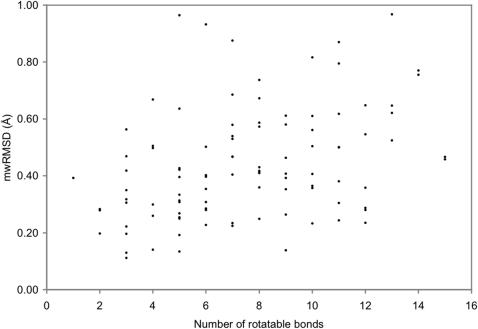
Distribution of the mass-weighted root mean square deviations (mwRMSDs) between the 100 ligand crystal structure conformations and their corresponding local minimum conformations obtained by using normal mode analysis among the number of rotatable bonds of these ligands.

Whereas other approaches such as performing a local conformational search to find multiple minima near the crystal structure conformation may find local minimum conformations with smaller mwRMSDs, this study already shows that all 100 and 69 of the 100 ligands have mwRMSDs less than 1.00 and 0.50 Å, respectively, between the ligand conformations in their complex crystal structures and the corresponding local minimum conformations. Although further studies with other force fields are desirable, given the similarity of the AMBER force field to other force fields and the results of this study, it is reasonable to suggest that ligands do not necessarily adopt their global minimum conformations when binding to proteins but they generally prefer to adopt local minimum conformations upon complexation. This preference seems to be independent of ligand properties such as the number of rotatable bonds because there is no correlation between the mwRMSD obtained with the NMA monitor (M1 column of Table 1) and the number of rotatable bonds for the 100 ligands under study (Figure 4). The results of this study lend support to the conformation-selection-theory–based virtual screening methods that use local minimum conformations generated by using the AMBER force field.

### Adverse effect of excessive energy minimization on chemical database development

**Figure 5:**
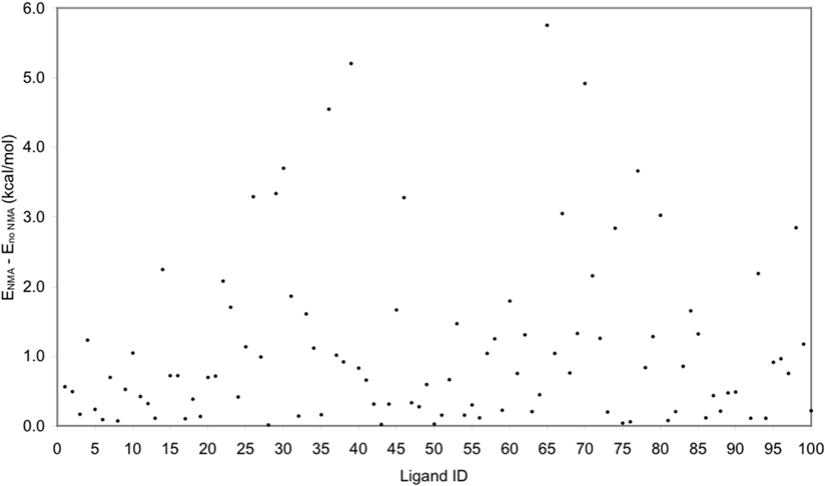
Scatterplot of the potential energy differences between two local minimum conformations obtained without and with normal mode analysis for the 100 ligands. The ligand ID is the row order of the ligand in Table S1—namely, the IDs of the ligands in the first and last rows of Table S1 are 1 and 100, respectively.

The results of this study suggest that excessive energy minimization of a ligand alone may fold or partially fold the ligand, leading to a conformation that is more stable in its free state but not suitable for docking into a protein. The partial folding caused by the conventional energy minimization method is common to the 100 ligands under study, as evident from the observation that the potential energies of all 100 ligand conformations obtained using the energy minimization without the NMA monitor are by average 1.1 kcal/mol lower than those using the NMA monitor except for the ligand of crystal structure 3STD (Figure 5). It is worth noting that the potential energy of the ligand local minimum conformation of crystal structure 3STD obtained without the NMA monitor is only 0.08 kcal/mol higher than that obtained with the NMA monitor. Docking a folded or partially folded ligand into a protein binding site requires computational effort to unfold the ligand. This effort is necessary in view of the entropy contribution to binding. This is because the unfolded ligand conformation such as the NEM-generated local minimum conformation is often captured at a *shallow* minimum of the ligand potential energy surface. The unfolded conformation at a shallow “energy well” can gain more entropic energy than the partially folded conformation at a deep “energy well,” which can outweigh the penalty in potential energy (≈1.1 kcal/mol in average) for moving the conformation from the deep “energy well” to the shallow “energy well.” This cautions about possible adverse effect of excessive energy minimization on generating a database of ligand conformations for virtual screening.

### Generating protein-bound ligand conformations

The NEM procedure described herein appears to be promising for generating unfolded or bound conformations *a priori*, given the fact that computers are nowadays fast enough to run NMA on drug-like ligands and even on proteins. For example, an Apple computer with two 2.0 GHz G5 processors can complete an NMA on a 153-residue protein TSG101 (PDB code: 1M4P [21]) and a 308-residue protein CCP (PDB code: 2AJ5 [22], [23]) in 21 minutes (using 320 MB memory) and 120 minutes (using 1.1 GB memory), respectively. The promise of the NEM procedure is further strengthened by the fact that the NEM procedure can be improved by enabling NMA to be performed on multiple processors and by guiding energy minimization with the information contained in the full Hessian matrix (Thompson and Pang's work in progress).

To test whether the NEM procedure using the AMBER force field can practically generate protein-bound ligand conformations from the two-dimensional (2D) ligand structures in the absence of their protein partners, six of the 100 protein-bound ligand crystal structures were selected as model systems. The selection criterion was that ligands do not have more than four conformation-governing rotatable bonds and this criterion was used in order to better test the necessity of the NEM procedure as described below. The PDB codes of these structures are 1BR6, 1EFY, 1L2S, 1QPE, 2CSN, and 4STD. The procedure for producing the six protein-bound ligand conformations from their 2D chemical structures *a priori* by using the NEM procedure and docking is detailed in METHODS. Briefly, it began with generation of a set of rotamers from the 2D ligand structure by systematically changing all conformation-governing rotatable bonds of the ligand in increments of 60° of arc starting from 0°. All these rotamers were then optimized to local minimum conformations by the NEM procedure. The resulting local minimum conformations were then subject to a cluster analysis with consideration of molecular symmetry to remove duplicated or similar conformations. Different local minimum conformations of the ligand were then docked into the binding site of the protein structure taken from the complex crystal structure using the EUDOC program [6], [8], [24]. The EUDOC-generated protein-bound ligand complex with the strongest intermolecular interaction energy was compared to the corresponding complex crystal structure. Two types of ligand mwRMSDs were calculated. One was calculated in the absence of the protein partner–namely, the ligand mwRMSD was calculated by superimposing the ligand structure of the complex crystal structure over the ligand structure of the EUDOC-generated protein-bound ligand complex. The other was calculated in complex with the protein partner–namely, the ligand mwRMSD was calculated by superimposing the protein structure of the complex crystal structure over the protein structure of the EUDOC-generated protein-bound ligand complex. As apparent from Table 2, both mwRMSDs are <1.00 Å for all six selected ligand crystal structures. These results demonstrate that the NEM procedure can indeed generate protein-bound conformations from their 2D ligand structures *a priori*.

**Table 2:** Reproduction of the Six Selected Protein-Ligand Complex Crystal Structures by Using the Normal-Mode-Analysis-Monitored Energy Minimization Procedure and Docking.

PDB code^1^	Torsions^2^	Conformers^3^	E_total_ 4 (kcal/mol)	E_vdw_ 5 (kcal/mol)	E_ele_ 6 (kcal/mol)	mwRMSD (Å)	
						no protein^7^	with protein^8^
1BR6	3	39	−103.6	−31.6	−72.0	0.35	0.49
1EFY	3	8	−59.3	−37.6	−21.7	0.44	0.80
1L2S	3	27	−98.9	−25.2	−73.7	0.31	0.93
1QPE	2	4	−50.7	−31.6	−19.1	0.31	0.49
2CSN	4	164	−87.1	−30.8	−56.3	0.65	0.96
4STD	4	40	−52.6	−42.2	−10.4	0.23	0.39

1 Protein Data Bank code;

2 number of the conformation-governing rotatable bonds of the ligand;

3 number of different ligand conformations obtained using the normal-mode-analysis-monitored energy minimization procedure;

4 intermolecular interaction energy calculated by the EUDOC program;

5 the van der Waals component of the intermolecular interaction energy;

6 the electrostatic component of the intermolecular interaction energy;

7 mass-weighted root mean square deviation of the ligand calculated in the absence of the protein partner;

8 mass-weighted root mean square deviation of the ligand calculated in complex with the protein partner.

**Figure 6:**
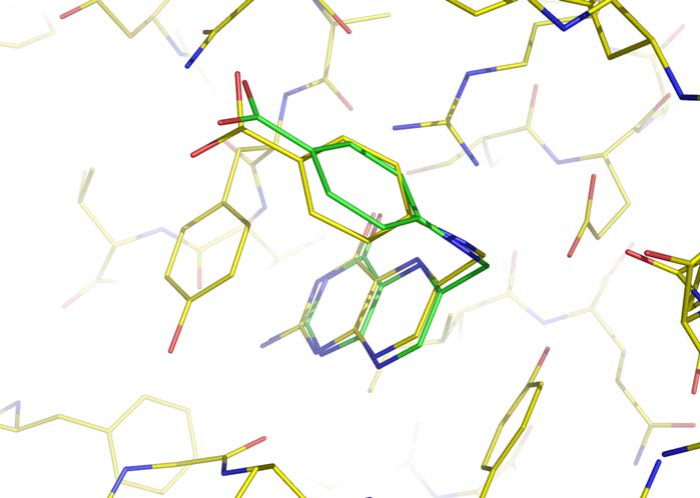
Overlay of crystal structure 1BR6 with the EUDOC-generated complex whose bound ligand conformation was generated by using the NEM procedure. The C atoms of the EUDOC-generated and crystal structures are green and yellow, respectively. The O and N atoms are red and blue, respectively. The mass-weighted root mean square deviation of the ligand conformation between EUDOC-generated and crystal structures is 0.49 Å.

**Figure 7:**
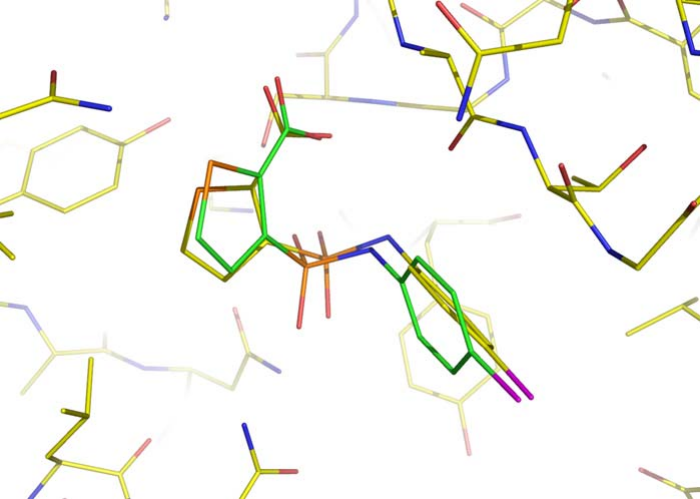
Overlay of crystal structure 1L2S with the EUDOC-generated complex whose bound ligand conformation was generated by using the NEM procedure. The C atoms of the EUDOC-generated and crystal structures are green and yellow, respectively. The O, N, and Cl atoms are red, blue, and magenta, respectively. The mass-weighted root mean square deviation of the ligand conformation between EUDOC-generated and crystal structures is 0.93 Å.

To evaluate the necessity of the NEM procedure for generating protein-bound ligand conformations to be used in docking, the above studies with the six selected ligands were repeated with the same procedure except that the rotamers were energy minimized using a conventional method that uses AMBER's default gradient cut-off of 10^−4^ kcal/(mol•Å) and does not use NMA to monitor the energy minimization progress. Interestingly, of the six ligands, the conventional energy minimization method failed to generate two protein-bound ligand conformations from their 2D ligand structures (PDB codes: 1BR6 and 1L2S). This result is in contrast to the prediction that the conventional energy minimization method might be able to generate ligand bound conformations from their 2D ligand structures in these cases, because the selected ligands are relatively rigid and they can hardly fold. In the case of the ligand in crystal structure 1BR6, using the NEM-generated local minimum conformations of the ligand, EUDOC identified a ligand bound conformation with the strongest interaction energy of –103.6 kcal/mol and an mwRMSD of 0.49 Å relative to the protein-bound ligand crystal structure (Figure 6); however, using the local minimum conformations generated by the conventional energy minimization method, EUDOC identified a ligand bound conformation with the strongest interaction energy of –88.3 kcal/mol and an mwRMSD of 5.33 Å relative to the protein-bound ligand crystal structure. These results demonstrate that excessive energy minimization caused partial folding of the rotamers of the relatively rigid ligand 1BR6. The partial folding consequently made the energy minimized rotamers less complementary to its protein partner both energetically and structurally (Table 3). The study with ligand 1L2S offered the same conclusion (Table 3 and Figure 7). These results suggest that the NEM procedure is not only practical but also desirable for improving the success rate of docking drug-like ligands through working with bound/unfolded local minimum conformations that have inherent entropic advantage as described above. A comprehensive study on the NEM procedure for generating small-molecule bound/unfolded local minimum conformations from their 2D structures via optimizing rotamers using the NEM procedure is in progress and will be reported in due course.

**Table 3:** Comparison of the Normal-Mode-Analysis-Monitored Energy Minimization Procedure (NEM) with the Conventional Energy Minimization Procedure (Conventional) for Reproducing Ligand-Protein Complex Crystal Structures.

PDB code^1^	NEM		Conventional	
	E_total_ 2 (kcal/mol)	mwRMSD^3^ (Å)	E_total_ 2 (kcal/mol)	mwRMSD^3^ (Å)
1BR6	−103.6	0.49	−88.3	5.33
1L2S	−98.9	0.93	−92.1	2.76

1 Protein Data Bank code;

2 intermolecular interaction energy calculated by the EUDOC program;

3 mass-weighted root mean square deviation of the ligand calculated in complex with the protein partner.

## Methods

### Program modules and force field parameter files

Due to varied performances obtained from code optimizations on the Apple G5 processors, the SANDER module of the AMBER 5 program [20] was used for energy minimization; the NMODE modules of the AMBER 5 and 8 programs [20] were used for transition-state structure search and NMA, respectively. The parm99.dat and gaff.dat files of the AMBER force field were used for proteins and small molecules, respectively.

### Preparation of the 100 protein-bound ligands

The 100 ligands used in this study were obtained from corresponding crystal structures of protein-ligand complexes available at the Protein Data Bank [25]. The hydrogen atoms of the ligands were added using the QUANTA97 program (Accelrys Software, Inc, San Diego, California). The protonation state of each ligand shown in Figures S2, S3, S4, S5 and S6 was determined according to p*K*­a values of the functional groups of the ligand and its nearby residues of the protein partner at pH 7.4. Because the coordinates of the *p*-nitroanilide group of the ligand in crystal structure 5TLN were undetermined, this group was manually added to the truncated ligand by using the QUANTA97 program; it was included in the energy minimization and NMA but excluded in the mwRMSD calculation. The force field parameters for ligand atom types that are unavailable in the AMBER force field parameter file were generated with the ANTECHAMBER module of the AMBER 7 program using gaff.dat [19] (Table S4 and Figures S7, S8, S9, S10, S11, S12 and S13). The atomic charges of all the ligands (Table S5) were generated according to the RESP procedure [26] with ab initio calculations at the HF/6-31G* level using the Gaussian98 program [27]. The topology and coordinate files of all the ligands used in energy minimization and NMA were generated with the PREP, LINK, EDIT, and PARM modules of the AMBER 5 program [20].

### Energy minimization and normal mode analysis

Energy minimization used (1) 10^6 ^steps of energy minimization, (2) a dielectric multiplicative constant of 80.0, (3) the steepest descent or conjugate gradient method, (4) a nonbonded cut-off of 12 Å, (9) a 10^−7^-kcal/(mole•Å) cut-off for the root-mean-square of the Cartesian elements of the gradient, and (10) defaults for other inputs of the SANDER module. NMA used (1) a dielectric multiplicative constant of 80.0, (2) a nonbonded cut-off of 12 Å, and (3) defaults for other inputs of the NMODE module. The transition state structures were obtained by using (1) 10^5 ^steps of energy minimization, (2) a flag to save coordinates at every step of the energy minimization, (3) initial minimization step length of 0.01 Å, (4) 200 eigenvectors, (5) the ordering of the “true” vibrational normal modes of ≤200, and (6) defaults for other inputs of the NMODE module.

### Generation of protein-bound conformations using the NEM Procedure

For each ligand, a set of local minimum conformations as potential protein-bound ligand conformations was generated from the 2D ligand structure in the absence of a protein partner according to the following steps. (1) A 2D ligand chemical structure was converted to a 3D structure by using the QUANTA97 program (Accelrys Software, Inc, San Diego, California). The atomic charges of the 3D ligand structure were generated using the same method described above for the atomic charges of the 100 protein-bound ligands. (2) New conformations of the 3D ligand structure were generated by systematically changing all conformation-governing rotatable bonds using the INTERFACE module of the AMBER 5 program [20] at a torsion increment of 60° of arc starting from 0°. The INTERFACE module generated 6^n^ conformations in total, where n is the number of conformation-governing rotatable bonds. The torsional restraints used by the INTERFACE module were set as parabolic to the designated angle ± 40° of arc and linear sides beyond that torsion range. The force constant used to restrain the conformation-governing rotatable bonds was set to 50 kcal/(mol•rad^2^). (3) Each conformation generated by the INTERFACE module was energy minimized to a local minimum conformation by using a Perl script for the NEM procedure (Figure S1). (4) The resulting local minimum conformations were subject to a cluster analysis with consideration of molecular symmetry. Each cluster contained all the conformations each of whose conformation-governing rotatable bonds is within ± 30° of arc of the average torsion of all conformations in the cluster. (5) One conformation was randomly chosen from each cluster as a representative conformation.

### Docking studies using the EUDOC program

The algorithm of the EUDOC program has been reported elsewhere [6]. Briefly, it uses a systematic search protocol, translating and rotating a ligand in a putative binding pocket of a protein and repeating the translations and rotations with different conformations of both protein and ligand to search for energetically favorable conformations, orientations, and positions of the ligand relative to those of the protein. A docking box is defined within the binding pocket to confine the translation of the ligand. The intermolecular interaction energy is the potential energy of the protein-ligand complex relative to the potential energies of both partners in their free state. This energy is calculated according to Equations 1 and 2 using the second-generation AMBER force field [18]. In calculating the intermolecular interaction energy, the multiplicative dielectric constant was set to 1.0, and the distance cut-offs for steric and electrostatic interactions were set to 10^9^ Å.
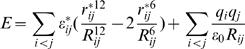
(Eq.1)

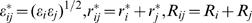
(Eq.2)In this study, a docking box was defined to enclose the ligand structure in the complex crystal structure; each dimension of the box is greater than 6 Å; the complex-prediction module of the EUDOC program was used to translate and rotate the ligand at translational and rotational increments of 1.0 Å and 10° of arc, respectively; the EUDOC program automatically repeated the translations and rotations with all different local minimum conformations of a ligand and one protein conformation taken from the complex crystal structure.

### Mass-weighted root mean square deviation calculations

The mwRMSDs of the 100 ligand local minimum conformations obtained using the NEM or conventional procedure relative to the corresponding crystal structures were calculated by superimposing the ligand local minimum conformation over the ligand conformation in the corresponding crystal structure using the PTRAJ module of the AMBER 8 program [20].

## Supporting Information

Table S1 Root mean square deviations, rotational and vibrational frequencies, and gradients of local minimum conformations obtained from normal-mode-analysis-monitored energy minimization of protein-bound ligand conformations taken from the 100 crystal structures using the conjugated gradient minimization method with a gradient cut-off of 0.06 kcal/(mol•Å). All three translational frequencies were zero and are not listed.
(0.04 MB PDF)

Table S2 Root mean square deviations, rotational and vibrational frequencies, and gradients of local minimum conformations obtained from normal-mode-analysis-monitored energy minimization of protein-bound ligand conformations taken from the 100 crystal structures using the steepest descent minimization method with a gradient cut-off of 0.06 kcal/(mol•Å). All three translational frequencies were zero and are not listed.
(0.04 MB PDF)

Table S3 Root mean square deviations, rotational and vibrational frequencies, and gradients of local minimum conformations obtained from normal-mode-analysis-monitored energy minimization of protein-bound ligand conformations taken from the 100 crystal structures using the steepest descent minimization method with a gradient cut-off of 0.01 kcal/(mol•Å). All three translational frequencies were zero and are not listed.
(0.04 MB PDF)

Table S4 The AMBER force field parameters developed for the 100 protein-bound ligands.
(0.04 MB PDF)

Table S5 The AMBER atom types and RESP charges of the 100 protein-bound ligands. The atom labels are shown in Figures S7, S8, S9, S10, S11, S12 and S13.
(0.21 MB PDF)

Figure S1 Perl script for the normal-mode-analysis-monitored energy minimization procedure.
(0.05 MB PDF)

Figure S2 Chemical structures and protonation states of the 100 protein-bound ligands (Part I).
(6.16 MB TIF)

Figure S3 Chemical structures and protonation states of the 100 protein-bound ligands (Part II).
(6.25 MB TIF)

Figure S4 Chemical structures and protonation states of the 100 protein-bound ligands (Part III).
(6.26 MB TIF)

Figure S5 Chemical structures and protonation states of the 100 protein-bound ligands (Part IV).
(6.21 MB TIF)

Figure S6 Chemical structures and protonation states of the 100 protein-bound ligands (Part V).
(4.23 MB TIF)

Figure S7 Definitions of atom labels of the 100 protein-bound ligands (Part I).
(6.46 MB TIF)

Figure S8 Definitions of atom labels of the 100 protein-bound ligands (Part II).
(6.28 MB TIF)

Figure S9 Definitions of atom labels of the 100 protein-bound ligands (Part III).
(6.47 MB TIF)

Figure S10 Definitions of atom labels of the 100 protein-bound ligands (Part IV).
(6.44 MB TIF)

Figure S11 Definitions of atom labels of the 100 protein-bound ligands (Part V).
(6.61 MB TIF)

Figure S12 Definitions of atom labels of the 100 protein-bound ligands (Part VI).
(6.55 MB TIF)

Figure S13 Definitions of atom labels of the 100 protein-bound ligands (Part VII).
(2.80 MB TIF)
