# Supplementary material for: Preference of Small Molecules for Local Minimum Conformations when Binding to Proteins
Source: PLoS One. 2007 Sep 5;2(9):e820. doi: 10.1371/journal.pone.0000820 (PMC1959118; doi:10.1371/journal.pone.0000820)
Supplement: Table S1 — Root mean square deviations, rotational and vibrational frequencies, and gradients of local minimum conformations obtained from normal-mode-analysis-monitored energy minimization of protein-bound ligand conformations taken from the 100 crystal structures using the conjugated gradient minimization method with a gradient cut-off of 0.06 kcal/(mol•Å). All three translational frequencies were zero and are not listed. (0.04 MB PDF) [file pone.0000820.s001.pdf]

Table S1. Root mean square deviations, rotational and vibrational frequencies, and gradients of local minimum conformations obtained from NMODE-monitored energy minimization of protein-bound ligand conformations taken from the 100 crystal structures using the conjugated gradient minimization method with a gradient cutoff of 0.06 kcal/(mol•Å). All three translational frequencies were zero and are not listed.

PDB[a] code	RSMD[b] (Å)	1st rot. freq. ilevel = 0[c]	2nd rot. freq. ilevel = 0	3rd rot. freq. ilevel = 0	1st vib. freq. ilevel = 0[d]	1st vib. freq. ilevel = 1[e]	Gradient[f] (kcal/mol•Å)
13GS	0.54	0.77	2.41	3.88	7.11	7.06	5.73E-02
1A42	0.69	0.00	0.00	3.14	13.24	13.24	5.62E-02
1A4K	0.36	0.00	2.14	2.76	14.27	14.27	5.56E-02
1A8T	0.88	0.00	0.00	1.61	3.01	0.00	5.83E-02
1AFQ	0.38	0.00	0.00	0.27	5.43	5.28	5.99E-02
1AOE	0.11	0.00	2.14	3.40	21.66	21.66	5.79E-02
1ATL	0.61	0.00	0.00	2.28	5.94	5.38	5.98E-02
1AZM	0.42	0.00	1.00	2.05	18.76	18.75	5.70E-02
1BNW	0.96	0.00	1.00	1.31	6.59	6.58	5.19E-02
1BQO	0.74	0.00	0.00	1.19	1.81	0.00	5.93E-02
1BR6	0.30	0.00	0.00	2.73	10.10	10.08	5.93E-02
1CET	0.25	0.00	0.00	3.10	8.29	8.08	5.93E-02
1CIM	0.39	0.00	1.22	3.01	23.41	23.40	5.91E-02
1D3P	0.50	0.00	0.00	0.56	2.46	0.00	5.30E-02
1D4P	0.93	0.00	0.00	2.87	12.00	12.00	5.98E-02
1D6V	0.59	0.00	0.00	3.48	12.36	12.35	5.77E-02
1DIB	0.41	0.00	0.00	1.09	11.76	11.76	5.89E-02
1DLR	0.26	0.00	0.00	0.00	3.81	0.00	5.56E-02
1EFY	0.35	0.00	0.00	1.53	33.61	33.61	5.74E-02
1ELA	0.97	0.00	0.00	1.80	9.48	9.48	5.83E-02
1ETR	0.24	0.00	0.00	3.00	9.18	9.12	5.93E-02
1ETT	0.35	0.00	0.00	0.00	0.93	0.00	4.62E-02
1EVE	0.40	0.00	1.57	2.06	10.22	10.09	5.88E-02
1EXA	0.13	0.42	1.37	2.73	6.11	6.05	5.62E-02
1EZQ	0.62	0.00	0.00	2.51	5.03	4.88	5.99E-02
1F0R	0.43	0.00	0.00	1.08	2.41	0.00	5.98E-02
1F0T	0.28	0.00	1.35	2.39	3.08	0.00	6.00E-02
1F4E	0.56	0.00	1.35	1.75	13.56	12.86	2.37E-02
1F4F	0.56	0.00	0.55	0.91	2.23	0.00	5.55E-02
1F4G	0.76	0.00	0.00	0.00	2.76	0.00	5.98E-02
1FCX	0.20	0.00	2.16	2.54	3.68	0.00	5.99E-02
1FCZ	0.25	0.00	0.00	3.40	24.65	24.64	5.99E-02
1FJS	0.58	0.00	0.00	0.00	1.74	0.00	5.96E-02
1FKG	0.87	0.00	0.00	2.68	4.15	3.81	6.00E-02
1FM6	0.23	0.00	0.00	0.00	7.69	7.68	5.92E-02
1FM9	0.36	0.00	0.00	0.00	1.18	0.00	3.63E-02
1FRB	0.50	0.00	0.00	0.00	0.92	0.00	5.70E-02
1G4O	0.42	0.55	1.78	3.31	14.84	14.83	5.97E-02
1GWX	0.62	0.00	1.68	2.74	3.30	2.58	5.96E-02
1H1P	0.32	0.00	0.00	0.90	1.76	0.00	3.87E-02
1H1S	0.50	0.00	0.00	2.39	12.31	12.30	5.74E-02
1H9U	0.13	0.00	0.00	3.14	20.72	20.72	5.75E-02
1HDQ	0.35	0.00	2.57	2.81	24.53	24.53	4.57E-02
1HFC	0.23	0.00	0.27	3.25	12.33	12.32	5.64E-02
1HPV	0.65	0.00	0.00	0.00	2.21	0.00	5.94E-02
1HTF	0.47	0.00	0.32	1.73	2.20	0.00	5.98E-02
1I7Z	0.25	0.00	0.00	2.90	21.82	21.78	5.78E-02
1I8Z	0.64	0.00	0.37	2.44	10.82	10.78	5.91E-02
1IF7	0.58	0.00	1.21	2.42	12.27	12.19	5.73E-02
1IY7	0.31	1.14	1.62	2.43	21.45	21.45	4.79E-02

1JSV	0.22	0.97	2.13	2.35	20.25	20.23	5.57E-02
1K1J	0.23	0.00	0.00	2.72	8.90	8.85	5.96E-02
1K22	0.50	0.00	0.00	0.45	2.61	0.00	5.87E-02
1K7E	0.40	0.00	0.00	2.11	4.80	4.17	4.95E-02
1K7F	0.40	0.85	2.86	3.37	13.43	13.39	5.58E-02
1KV1	0.27	0.00	0.11	1.85	9.41	9.39	5.88E-02
1KV2	0.50	0.00	0.00	0.00	0.92	0.00	5.83E-02
1L2S	0.51	0.71	2.21	2.60	17.49	17.48	5.79E-02
1L8G	0.40	0.00	2.13	2.29	9.92	9.92	5.90E-02
1LQD	0.47	0.00	0.00	0.00	0.67	0.00	3.91E-02
1M48	0.14	0.00	0.00	1.51	3.00	0.00	4.93E-02
1MMB	0.46	0.00	1.30	2.33	7.59	7.57	5.97E-02
1MNC	0.28	0.00	0.82	2.91	10.76	10.73	5.88E-02
1MQ5	0.41	0.00	1.98	2.36	8.00	7.94	5.98E-02
1MQ6	0.36	0.00	0.00	0.33	1.13	0.00	5.99E-02
1NHU	0.57	0.00	0.00	1.83	3.28	0.00	6.00E-02
1NHV	0.61	0.00	0.21	0.63	5.78	5.36	5.94E-02
1O86	0.52	0.00	1.14	2.46	11.91	11.90	5.69E-02
1OHR	0.55	0.00	1.47	2.54	10.15	10.13	5.73E-02
1PPC	0.30	0.00	0.00	2.00	6.57	6.47	5.60E-02
1PPH	0.42	0.00	0.00	0.00	8.02	8.00	3.34E-02
1QBU	0.79	0.00	1.65	2.19	5.41	5.35	5.98E-02
1QHI	0.23	0.00	0.00	1.30	13.74	13.72	5.99E-02
1QL9	0.31	0.00	0.00	2.18	2.99	0.00	5.96E-02
1QPE	0.28	0.00	0.00	1.42	28.20	28.19	5.42E-02
1R09	0.47	0.00	0.00	0.00	2.17	0.00	5.65E-02
1SYN	0.67	0.00	1.56	1.93	2.77	0.00	5.94E-02
1THL	0.29	1.45	2.05	2.86	5.90	5.83	5.91E-02
1UVS	0.65	0.00	0.00	1.08	2.80	0.00	5.92E-02
1UVT	0.43	0.00	0.00	0.00	1.90	0.00	5.00E-02
1YDR	0.28	0.89	2.17	3.16	11.22	11.19	5.92E-02
1YDS	0.33	0.00	2.69	3.34	19.51	19.50	5.70E-02
1YDT	0.41	0.00	0.00	0.00	1.33	0.00	3.40E-02
2CGR	0.46	0.00	0.00	0.00	1.99	0.00	5.57E-02
2CSN	0.67	0.00	0.00	3.09	3.50	0.00	5.62E-02
2PCP	0.20	0.00	1.34	2.67	37.18	37.18	5.83E-02
2QWI	0.39	0.00	0.00	1.90	23.23	23.22	5.93E-02
3CPA	0.31	0.00	1.49	3.45	15.26	15.17	5.94E-02
3ERK	0.31	0.00	0.00	0.00	1.69	0.00	5.99E-02
3ERT	0.26	1.83	2.37	2.91	16.17	16.16	5.97E-02
3STD	0.29	0.00	0.00	4.08	4.52	0.00	5.84E-02
3TMN	0.22	0.00	0.00	2.76	10.51	10.44	5.95E-02
4DFR	0.36	0.00	0.00	1.32	2.97	0.00	5.93E-02
4STD	0.14	0.00	0.00	0.00	13.37	13.36	5.91E-02
5STD	0.19	0.00	0.81	2.34	4.74	3.06	4.20E-02
5TLN	0.77	0.00	1.24	2.25	7.97	7.93	6.00E-02
7DFR	0.82	0.00	0.00	0.00	5.04	0.00	5.99E-02
7EST	0.41	0.00	0.00	0.00	1.30	0.00	4.04E-02
830C	0.53	0.00	0.00	1.69	2.49	0.00	5.99E-02
966C	0.47	0.00	0.00	1.54	12.15	12.13	5.98E-02

a: PDB: Protein Data Bank

b: RMSD of all ligand atoms between minimized and initial conformations.

c: First rotational frequency (cm^{-1}) computed with the second derivative matrix not adjusted to put rotation and translation vectors to a high frequency (ilevel = 0).

d: First vibrational frequency (cm^{-1}) computed with the second derivative matrix not adjusted to put rotation and translation vectors to a high frequency (ilevel = 0).

e: First vibrational frequency (cm^{-1}) computed with the second derivative matrix adjusted to put rotation and translation vectors to a high frequency (ilevel = 1).

f: Root mean square gradient.
