# Supplementary material for: Preference of Small Molecules for Local Minimum Conformations when Binding to Proteins
Source: PLoS One. 2007 Sep 5;2(9):e820. doi: 10.1371/journal.pone.0000820 (PMC1959118; doi:10.1371/journal.pone.0000820)
Supplement: Table S2 — Root mean square deviations, rotational and vibrational frequencies, and gradients of local minimum conformations obtained from normal-mode-analysis-monitored energy minimization of protein-bound ligand conformations taken from the 100 crystal structures using the steepest descent minimization method with a gradient cut-off of 0.06 kcal/(mol•Å). All three translational frequencies were zero and are not listed. (0.04 MB PDF) [file pone.0000820.s002.pdf]

Table S2. Root mean square deviations, rotational and vibrational frequencies, and gradients of local minimum conformations obtained from NMODE-monitored energy minimization of protein-bound ligand conformations taken from the 100 crystal structures using the steepest descent minimization method with a gradient cutoff of 0.06 kcal/(mol•Å). All three translational frequencies were zero and are not listed.

PDB[a] code	RSMD[b] (Å)	1st rot. freq. ilevel = 0[c]	2nd rot. freq. ilevel = 0	3rd rot. freq. ilevel = 0	1st vib. freq. ilevel = 0[d]	1st vib. freq. ilevel = 1[e]	Gradient[f] (kcal/mol•Å)
13GS	0.51	0.00	2.62	3.81	5.90	5.76	5.97E-02
1A42	0.54	0.00	0.00	3.08	13.37	13.35	5.82E-02
1A4K	0.32	0.00	2.02	2.63	14.46	14.46	5.84E-02
1A8T	0.85	0.00	0.00	0.39	3.00	0.00	5.95E-02
1AFQ	0.30	0.00	0.00	2.89	5.11	4.75	5.97E-02
1AOE	0.10	0.00	0.00	2.94	21.88	21.87	5.31E-02
1ATL	0.62	0.00	0.00	3.49	6.80	6.33	5.99E-02
1AZM	0.36	0.00	0.84	2.24	18.43	18.42	5.82E-02
1BNW	0.90	0.00	0.00	0.00	7.68	7.54	5.75E-02
1BQO	0.58	0.00	0.00	1.85	2.71	0.00	5.95E-02
1BR6	0.25	0.00	0.00	3.24	10.03	9.99	5.70E-02
1CET	0.18	0.00	0.00	2.50	8.54	8.23	5.96E-02
1CIM	0.31	0.00	0.00	1.73	22.19	22.19	5.92E-02
1D3P	0.55	0.00	0.00	0.45	2.10	0.00	5.61E-02
1D4P	0.63	0.00	0.00	4.91	5.53	5.39	5.94E-02
1D6V	0.53	0.00	0.00	3.16	11.12	11.10	5.24E-02
1DIB	0.38	0.00	1.31	1.86	11.88	11.88	5.37E-02
1DLR	0.22	0.00	0.00	1.73	4.02	0.00	5.99E-02
1EFY	0.29	0.00	0.00	2.11	30.79	30.79	5.90E-02
1ELA	0.95	0.00	1.08	2.05	3.48	3.46	6.00E-02
1ETR	0.22	0.00	0.00	3.70	8.69	8.53	5.99E-02
1ETT	0.37	0.00	0.00	0.00	0.08	0.00	3.24E-02
1EVE	0.17	0.00	0.00	1.23	5.27	0.00	5.91E-02
1EXA	0.12	0.00	0.00	2.04	5.39	5.09	5.71E-02
1EZQ	0.56	0.00	0.00	0.00	4.44	0.00	5.58E-02
1F0R	0.36	0.00	0.00	0.30	2.17	1.15	5.54E-02
1F0T	0.28	0.00	1.98	2.72	3.12	0.00	5.89E-02
1F4E	0.57	0.00	1.09	1.29	3.86	0.00	2.01E-02
1F4F	0.57	0.00	0.40	1.04	1.92	0.00	4.90E-02
1F4G	0.61	0.00	0.00	0.00	5.27	4.26	5.58E-02
1FCX	0.15	0.00	1.14	2.08	4.54	0.00	5.90E-02
1FCZ	0.15	0.00	0.00	3.33	23.89	23.88	5.39E-02
1FJS	0.53	0.00	0.00	1.69	1.92	0.00	5.76E-02
1FKG	0.69	0.00	0.00	0.00	3.75	0.00	5.96E-02
1FM6	0.20	0.00	0.00	3.86	7.93	7.90	5.86E-02
1FM9	0.36	0.00	0.00	0.00	1.35	0.00	3.58E-02
1FRB	0.51	0.00	0.00	0.00	0.05	0.00	3.07E-02
1G4O	0.30	0.00	0.00	0.00	1.36	0.00	5.94E-02
1GWX	0.54	0.00	0.00	1.99	3.03	0.00	5.94E-02
1H1P	0.23	0.00	0.00	2.31	4.88	0.00	5.67E-02
1H1S	0.37	0.00	0.00	2.95	11.73	11.69	5.66E-02
1H9U	0.10	0.00	1.66	3.13	20.38	20.38	5.99E-02
1HDQ	0.33	0.00	2.30	2.56	24.53	24.51	4.90E-02
1HFC	0.18	0.00	0.00	3.62	11.03	10.97	5.98E-02
1HPV	0.69	0.00	0.00	2.05	2.82	0.00	5.92E-02
1HTF	0.53	0.00	0.00	0.79	1.53	0.00	5.98E-02
1I7Z	0.20	0.00	0.00	2.94	23.04	22.94	5.65E-02
1I8Z	0.55	0.00	0.00	2.75	9.31	9.08	5.18E-02
1IF7	0.43	0.00	0.00	2.63	11.73	11.42	5.98E-02
1IY7	0.26	0.00	0.82	2.93	21.57	21.55	5.68E-02

1JSV	0.20	0.00	1.99	2.91	20.83	20.82	5.91E-02
1K1J	0.19	0.00	0.50	3.42	8.87	8.63	5.69E-02
1K22	0.45	0.00	0.00	0.38	3.62	0.00	5.94E-02
1K7E	0.32	0.00	0.00	0.86	4.88	3.23	5.96E-02
1K7F	0.36	0.00	3.13	3.57	14.65	14.57	5.97E-02
1KV1	0.23	0.00	0.00	2.23	12.86	12.82	5.89E-02
1KV2	0.52	0.00	0.00	0.00	0.83	0.00	4.02E-02
1L2S	0.30	0.00	0.00	1.50	2.05	0.00	5.96E-02
1L8G	0.37	0.00	2.14	2.52	9.69	9.69	5.80E-02
1LQD	0.51	0.00	0.00	0.00	0.37	0.00	4.25E-02
1M48	0.17	0.00	0.00	0.00	1.10	0.00	5.87E-02
1MMB	0.37	0.00	0.00	2.47	3.83	0.00	5.98E-02
1MNC	0.22	0.00	0.00	2.76	9.28	9.17	5.97E-02
1MQ5	0.34	0.00	2.56	3.31	7.37	7.14	5.94E-02
1MQ6	0.32	0.00	1.02	1.66	3.18	0.00	5.61E-02
1NHU	0.55	0.00	0.00	0.00	0.49	0.00	5.53E-02
1NHV	0.59	0.00	1.31	1.73	6.21	5.40	6.00E-02
1O86	0.52	0.00	1.74	2.71	13.30	13.29	5.84E-02
1OHR	0.41	0.00	2.21	3.46	11.04	10.86	5.83E-02
1PPC	0.22	0.00	0.00	0.00	2.67	0.00	5.99E-02
1PPH	0.41	0.00	0.00	1.06	2.90	0.00	5.24E-02
1QBU	0.59	0.00	2.24	2.63	5.17	4.88	5.61E-02
1QHI	0.21	0.00	0.00	3.00	13.87	13.82	5.86E-02
1QL9	0.31	0.00	0.00	1.54	2.49	0.00	5.87E-02
1QPE	0.25	0.00	0.00	1.33	27.13	27.13	5.97E-02
1R09	0.46	0.00	1.98	2.35	4.12	0.00	3.31E-02
1SYN	0.89	0.00	1.77	2.06	2.68	0.00	4.68E-02
1THL	0.27	1.73	2.30	3.03	4.99	4.91	5.91E-02
1UVS	0.58	0.00	0.00	0.00	2.35	0.00	6.00E-02
1UVT	0.46	0.00	0.00	0.00	3.06	0.00	5.90E-02
1YDR	0.24	0.00	1.06	2.90	7.51	6.91	5.98E-02
1YDS	0.32	0.00	4.49	5.22	19.12	19.06	5.48E-02
1YDT	0.43	0.00	0.00	0.00	0.14	0.00	3.33E-02
2CGR	0.44	0.00	0.00	0.00	2.12	0.00	5.37E-02
2CSN	0.52	0.00	4.25	4.80	13.93	13.90	5.96E-02
2PCP	0.16	0.00	2.56	4.16	42.28	42.28	5.95E-02
2QWI	0.37	0.00	0.00	1.85	24.96	24.95	5.84E-02
3CPA	0.24	0.00	1.60	3.98	11.36	11.34	5.97E-02
3ERK	0.37	0.00	0.00	0.00	20.33	20.33	5.93E-02
3ERT	0.21	0.00	2.19	3.71	16.22	16.10	5.98E-02
3STD	0.38	0.00	3.87	4.42	12.15	11.97	5.58E-02
3TMN	0.20	0.00	0.00	2.01	6.61	5.14	5.76E-02
4DFR	0.29	0.00	0.00	1.07	2.71	0.00	5.47E-02
4STD	0.13	0.00	0.00	3.70	12.97	12.96	5.94E-02
5STD	0.16	0.00	0.21	1.80	5.37	0.00	5.54E-02
5TLN	0.78	0.00	1.04	2.14	7.87	7.84	5.91E-02
7DFR	0.65	0.00	0.00	0.00	4.88	0.00	5.99E-02
7EST	0.39	0.00	0.00	0.00	0.11	0.00	5.73E-02
830C	0.53	0.00	0.00	1.69	2.49	0.00	5.99E-02
966C	0.44	0.00	0.00	0.00	2.09	0.00	5.97E-02

a: PDB: Protein Data Bank
b: RMSD of all ligand atoms between minimized and initial conformations.
c: First rotational frequency (cm^{-1}) computed with the second derivative matrix not adjusted to put rotation and translation vectors to a high frequency (ilevel = 0).
d: First vibrational frequency (cm^{-1}) computed with the second derivative matrix not adjusted to put rotation and translation vectors to a high frequency (ilevel = 0).
e: First vibrational frequency (cm^{-1}) computed with the second derivative matrix adjusted to put rotation and translation vectors to a high frequency (ilevel = 1).
f: Root mean square gradient.
