# Supplementary material for: Preference of Small Molecules for Local Minimum Conformations when Binding to Proteins
Source: PLoS One. 2007 Sep 5;2(9):e820. doi: 10.1371/journal.pone.0000820 (PMC1959118; doi:10.1371/journal.pone.0000820)
Supplement: Table S3 — Root mean square deviations, rotational and vibrational frequencies, and gradients of local minimum conformations obtained from normal-mode-analysis-monitored energy minimization of protein-bound ligand conformations taken from the 100 crystal structures using the steepest descent minimization method with a gradient cut-off of 0.01 kcal/(mol•Å). All three translational frequencies were zero and are not listed. (0.04 MB PDF) [file pone.0000820.s003.pdf]

Table S3. Root mean square deviations, rotational and vibrational frequencies, and gradients of local minimum conformations obtained from NMODE-monitored energy minimization of protein-bound ligand conformations taken from the 100 crystal structures using the steepest descent minimization method with a gradient cutoff of 0.01 kcal/(mol•Å). All three translational frequencies were zero and are not listed.

| PDB code | RSMD[a] (Å) | 1st rot. freq. ilevel = 0[b] | 2nd rot. freq. ilevel = 0 | 3rd rot. freq. ilevel = 0 | 1st vib. freq. ilevel = 0[c] | 1st vib. freq. ilevel = 1[d] | Gradient[e] (kcal/mol•Å) |
|---|---|---|---|---|---|---|---|
| 13GS | 0.68 | 0.00 | 1.16 | 1.59 | 7.10 | 7.10 | 8.39E-03 |
| 1A42 | 0.91 | 0.00 | 0.19 | 1.44 | 12.76 | 12.76 | 9.43E-03 |
| 1A4K | 0.41 | 0.00 | 1.29 | 1.77 | 13.88 | 13.88 | 9.46E-03 |
| 1A8T | 1.06 | 0.00 | 0.05 | 1.46 | 6.27 | 6.27 | 9.30E-03 |
| 1AFQ | 0.46 | 0.00 | 0.00 | 0.46 | 6.88 | 6.87 | 9.78E-03 |
| 1AOE | 0.15 | 0.00 | 0.00 | 1.10 | 16.80 | 16.80 | 7.02E-03 |
| 1ATL | 0.76 | 0.00 | 0.00 | 1.20 | 4.95 | 4.95 | 9.14E-03 |
| 1AZM | 0.49 | 0.00 | 0.36 | 0.75 | 18.34 | 18.34 | 8.20E-03 |
| 1BNW | 1.20 | 0.00 | 0.41 | 0.72 | 9.10 | 9.10 | 9.20E-03 |
| 1BQO | 1.00 | 0.00 | 0.00 | 1.71 | 7.73 | 7.73 | 8.85E-03 |
| 1BR6 | 0.56 | 0.00 | 0.00 | 1.70 | 9.06 | 9.06 | 9.13E-03 |
| 1CET | 0.38 | 0.00 | 0.00 | 1.16 | 6.85 | 6.82 | 9.22E-03 |
| 1CIM | 0.54 | 0.00 | 0.50 | 1.22 | 21.52 | 21.52 | 9.31E-03 |
| 1D3P | 0.94 | 0.00 | 0.81 | 1.56 | 7.81 | 7.80 | 9.09E-03 |
| 1D4P | 1.21 | 0.00 | 0.00 | 2.20 | 16.86 | 16.86 | 9.06E-03 |
| 1D6V | 0.81 | 0.00 | 0.00 | 0.36 | 13.41 | 13.41 | 9.92E-03 |
| 1DIB | 0.43 | 0.00 | 0.00 | 0.65 | 11.65 | 11.65 | 9.28E-03 |
| 1DLR | 0.41 | 0.00 | 0.68 | 1.84 | 8.91 | 8.89 | 8.76E-03 |
| 1EFY | 0.41 | 0.00 | 0.18 | 0.50 | 34.30 | 34.30 | 9.35E-03 |
| 1ELA | 1.08 | 0.00 | 0.36 | 0.87 | 9.27 | 9.27 | 9.44E-03 |
| 1ETR | 0.40 | 0.00 | 0.57 | 1.69 | 9.62 | 9.62 | 9.14E-03 |
| 1ETT | 0.68 | 0.00 | 0.20 | 1.79 | 7.71 | 7.71 | 8.33E-03 |
| 1EVE | 0.44 | 0.00 | 0.44 | 1.01 | 10.42 | 10.41 | 9.75E-03 |
| 1EXA | 0.16 | 0.00 | 1.07 | 1.35 | 4.60 | 4.60 | 9.60E-03 |
| 1EZQ | 0.88 | 0.00 | 0.79 | 1.42 | 8.00 | 8.00 | 9.99E-03 |
| 1F0R | 1.42 | 0.00 | 0.72 | 1.68 | 2.12 | 1.02 | 9.21E-03 |
| 1F0T | 0.63 | 0.00 | 0.00 | 1.54 | 5.23 | 5.23 | 9.51E-03 |
| 1F4E | 0.57 | 0.00 | 0.72 | 0.92 | 2.19 | 0.00 | 9.10E-03 |
| 1F4F | 1.54 | 0.00 | 0.66 | 0.79 | 12.54 | 12.54 | 8.74E-03 |
| 1F4G | 1.64 | 0.00 | 0.56 | 0.83 | 8.20 | 8.20 | 9.85E-03 |
| 1FCX | 0.94 | 0.00 | 0.95 | 1.32 | 7.65 | 7.65 | 9.46E-03 |
| 1FCZ | 0.34 | 0.00 | 0.00 | 0.00 | 24.82 | 24.82 | 9.96E-03 |
| 1FJS | 0.77 | 0.00 | 0.00 | 0.70 | 4.74 | 4.74 | 9.64E-03 |
| 1FKG | 1.12 | 0.00 | 0.00 | 1.56 | 6.52 | 6.51 | 9.89E-03 |
| 1FM6 | 0.30 | 0.00 | 0.00 | 1.29 | 7.96 | 7.96 | 9.70E-03 |
| 1FM9 | 0.42 | 0.00 | 0.00 | 0.53 | 1.30 | 0.00 | 8.40E-03 |
| 1FRB | 1.03 | 0.00 | 1.27 | 1.42 | 4.21 | 4.21 | 9.89E-03 |
| 1G4O | 0.86 | 0.00 | 0.24 | 1.13 | 11.97 | 11.97 | 8.84E-03 |
| 1GWX | 1.63 | 0.00 | 0.53 | 1.76 | 5.34 | 5.34 | 9.44E-03 |
| 1H1P | 0.68 | 0.00 | 0.00 | 0.22 | 17.29 | 17.29 | 7.67E-03 |
| 1H1S | 0.74 | 0.00 | 0.00 | 1.27 | 12.98 | 12.98 | 9.63E-03 |
| 1H9U | 0.26 | 0.00 | 0.00 | 1.42 | 21.95 | 21.95 | 6.86E-03 |
| 1HDQ | 0.38 | 0.00 | 0.00 | 1.04 | 24.13 | 24.13 | 7.42E-03 |
| 1HFC | 0.33 | 0.00 | 0.81 | 1.18 | 13.87 | 13.87 | 9.47E-03 |
| 1HPV | 1.08 | 0.00 | 0.62 | 1.07 | 8.99 | 8.98 | 9.73E-03 |
| 1HTF | 1.09 | 0.00 | 0.00 | 1.23 | 1.46 | 0.00 | 8.58E-03 |
| 1I7Z | 0.38 | 0.00 | 0.00 | 1.48 | 18.01 | 18.01 | 8.14E-03 |
| 1I8Z | 0.69 | 0.00 | 0.58 | 1.35 | 13.15 | 13.15 | 9.85E-03 |
| 1IF7 | 0.84 | 0.00 | 0.92 | 1.21 | 12.64 | 12.63 | 9.49E-03 |
| 1IY7 | 0.35 | 0.00 | 0.47 | 0.70 | 21.79 | 21.79 | 9.96E-03 |

| | | | | | | | |
|---|---|---|---|---|---|---|---|
| 1JSV | 0.32 | 0.00 | 0.00 | 0.00 | 18.35 | 18.35 | 9.74E-03 |
| 1K1J | 0.45 | 0.00 | 0.00 | 1.46 | 9.97 | 9.97 | 9.37E-03 |
| 1K22 | 1.09 | 0.00 | 1.33 | 1.58 | 8.40 | 8.39 | 9.94E-03 |
| 1K7E | 0.53 | 0.00 | 0.00 | 0.70 | 5.75 | 5.74 | 8.79E-03 |
| 1K7F | 0.53 | 0.00 | 1.31 | 1.40 | 12.35 | 12.35 | 9.98E-03 |
| 1KV1 | 0.31 | 0.00 | 0.53 | 1.79 | 10.76 | 10.76 | 9.60E-03 |
| 1KV2 | 0.73 | 0.00 | 0.00 | 0.88 | 5.34 | 5.34 | 9.08E-03 |
| 1L2S | 0.62 | 0.00 | 0.99 | 1.24 | 5.31 | 5.30 | 8.09E-03 |
| 1L8G | 0.50 | 0.00 | 0.00 | 0.48 | 9.58 | 9.58 | 6.20E-03 |
| 1LQD | 1.10 | 0.00 | 0.00 | 1.99 | 6.35 | 6.35 | 9.69E-03 |
| 1M48 | 0.31 | 0.00 | 0.37 | 0.97 | 1.78 | 0.00 | 8.97E-03 |
| 1MMB | 0.50 | 0.00 | 0.00 | 1.17 | 8.61 | 8.61 | 9.97E-03 |
| 1MNC | 0.33 | 0.00 | 0.95 | 1.44 | 11.14 | 11.14 | 8.73E-03 |
| 1MQ5 | 0.54 | 0.00 | 0.33 | 0.88 | 8.64 | 8.64 | 9.72E-03 |
| 1MQ6 | 0.46 | 0.00 | 0.00 | 0.00 | 0.88 | 0.00 | 9.96E-03 |
| 1NHU | 0.70 | 0.00 | 0.00 | 1.45 | 5.63 | 5.63 | 9.55E-03 |
| 1NHV | 0.63 | 0.00 | 0.85 | 1.02 | 6.30 | 6.29 | 9.95E-03 |
| 1O86 | 0.74 | 0.00 | 0.00 | 0.91 | 12.74 | 12.74 | 7.98E-03 |
| 1OHR | 0.93 | 0.00 | 0.85 | 1.37 | 8.22 | 8.22 | 9.39E-03 |
| 1PPC | 1.00 | 0.00 | 0.00 | 1.80 | 6.38 | 6.37 | 9.60E-03 |
| 1PPH | 0.45 | 0.00 | 0.00 | 1.76 | 8.60 | 8.60 | 9.14E-03 |
| 1QBU | 1.11 | 0.00 | 0.89 | 1.02 | 4.76 | 4.66 | 9.39E-03 |
| 1QHI | 0.29 | 0.00 | 0.00 | 0.86 | 16.13 | 16.13 | 9.43E-03 |
| 1QL9 | 0.64 | 0.00 | 0.00 | 1.45 | 2.03 | 0.00 | 9.96E-03 |
| 1QPE | 0.30 | 0.00 | 0.00 | 0.59 | 28.78 | 28.78 | 6.95E-03 |
| 1R09 | 0.50 | 0.00 | 1.39 | 1.51 | 2.97 | 2.89 | 7.76E-03 |
| 1SYN | 2.33 | 0.00 | 0.00 | 0.87 | 1.16 | 0.00 | 8.99E-03 |
| 1THL | 0.53 | 0.00 | 0.00 | 1.31 | 8.00 | 8.00 | 9.75E-03 |
| 1UVS | 0.94 | 0.00 | 0.55 | 1.26 | 6.16 | 6.16 | 9.24E-03 |
| 1UVT | 0.79 | 0.00 | 0.00 | 0.00 | 1.41 | 0.00 | 9.88E-03 |
| 1YDR | 0.31 | 0.00 | 0.00 | 1.43 | 13.37 | 13.37 | 8.73E-03 |
| 1YDS | 0.41 | 0.00 | 1.15 | 1.30 | 25.03 | 25.03 | 8.12E-03 |
| 1YDT | 0.75 | 0.00 | 0.00 | 0.99 | 1.32 | 0.00 | 9.53E-03 |
| 2CGR | 1.12 | 0.00 | 0.98 | 1.58 | 7.23 | 7.23 | 9.42E-03 |
| 2CSN | 0.96 | 0.00 | 1.32 | 1.63 | 37.68 | 37.68 | 7.64E-03 |
| 2PCP | 0.24 | 0.00 | 0.00 | 1.43 | 33.14 | 33.14 | 8.50E-03 |
| 2QWI | 0.49 | 0.00 | 0.92 | 1.18 | 25.45 | 25.45 | 1.00E-02 |
| 3CPA | 0.43 | 0.00 | 0.40 | 0.83 | 11.60 | 11.59 | 7.43E-03 |
| 3ERK | 0.42 | 0.00 | 0.00 | 1.20 | 21.72 | 21.72 | 9.67E-03 |
| 3ERT | 0.39 | 0.00 | 0.77 | 0.99 | 17.96 | 17.96 | 8.88E-03 |
| 3STD[f] | 0.72 | 0.69 | 1.11 | 1.28 | 13.34 | 13.34 | 3.81E-03 |
| 3TMN | 0.24 | 0.00 | 1.12 | 1.45 | 14.37 | 14.37 | 9.90E-03 |
| 4DFR | 1.22 | 0.00 | 1.39 | 1.63 | 6.60 | 6.60 | 9.89E-03 |
| 4STD | 0.22 | 0.00 | 0.00 | 0.72 | 16.83 | 16.83 | 8.82E-03 |
| 5STD | 0.60 | 0.00 | 1.10 | 1.33 | 14.60 | 14.60 | 9.54E-03 |
| 5TLN | 1.06 | 0.00 | 0.58 | 0.69 | 7.06 | 7.05 | 9.21E-03 |
| 7DFR | 1.14 | 0.00 | 0.14 | 0.78 | 6.23 | 6.20 | 7.90E-03 |
| 7EST | 0.66 | 0.00 | 0.00 | 1.35 | 5.70 | 5.70 | 9.83E-03 |
| 830C | 1.32 | 0.00 | 0.52 | 1.03 | 8.55 | 8.55 | 8.66E-03 |
| 966C | 0.51 | 0.00 | 0.00 | 1.22 | 15.07 | 15.07 | 9.15E-03 |

a: PDB: Protein Data Bank
b: RMSD of all ligand atoms between minimized and initial conformations.
c: First rotational frequency ($cm^{-1}$) computed with the second derivative matrix not adjusted to put rotation and translation vectors to a high frequency (ilevel = 0).
d: First vibrational frequency ($cm^{-1}$) computed with the second derivative matrix not adjusted to put rotation and translation vectors to a high frequency (ilevel = 0).
e: First vibrational frequency ($cm^{-1}$) computed with the second derivative matrix adjusted to put rotation and translation vectors to a high frequency (ilevel = 1).
f: Root mean square gradient.
g: Required cycles of 20 steps instead of 10 steps due to numeric truncations.
