# Supplementary material for: Preference of Small Molecules for Local Minimum Conformations when Binding to Proteins
Source: PLoS One. 2007 Sep 5;2(9):e820. doi: 10.1371/journal.pone.0000820 (PMC1959118; doi:10.1371/journal.pone.0000820)
Supplement: Table S4 — The AMBER force field parameters developed for the 100 protein-bound ligands. (0.04 MB PDF) [file pone.0000820.s004.pdf]

Table S4. The AMBER force field parameters developed for the 100 protein-bound ligands.

MASS		
Br	79.9	2.88
C	12.01	0.616
C*	12.01	0.36
C2	12.01	0.36
CA	12.01	0.36
CB	12.01	0.36
CC	12.01	0.36
CD	12.01	0.36
CG	12.01	0.36
CK	12.01	0.36
Cl	35.45	1.91
CM	12.01	0.36
CN	12.01	0.36
CR	12.01	0.36
CT	12.01	0.878
CV	12.01	0.36
CW	12.01	0.36
CX	12.01	0.36
CZ	12.01	0.36
F	19	0.32
H	1.008	0.161
H1	1.008	0.135
H2	1.008	0.135
H4	1.008	0.135
H5	1.008	0.135
HA	1.008	0.135
HC	1.008	0.135
HO	1.008	0.135
HP	1.008	0.135
HS	1.008	0.135
N	14.01	0.53
N*	14.01	0.53
N1	14.01	0.53
N2	14.01	0.53
N3	14.01	0.53
NA	14.01	0.53
NB	14.01	0.53
NC	14.01	0.53
NE	14.01	0.53
NF	14.01	0.53
NH	14.01	0.53
NO	14.01	0.53
NT	14.01	0.53
O	16	0.434
OH	16	0.465
OS	16	0.465
S	32.06	2.9
S6	32.06	2.9
SH	32.06	2.9
SY	32.06	2.9
BOND		
C*-C*	473.7	1.39
C*-Cl	322.8	1.729
C*-CM	473.7	1.39
C*-CV	473.7	1.39
C*-OS	364	1.38
C2-N2	581.1	1.288
C2-N3	309.1	1.482

C2-NH	462.6	1.355
C2-OS	392.6	1.357
CA-C*	366	1.472
CA-CA	478.4	1.387
CA-CC	473.7	1.39
CA-CG	406.6	1.438
CA-CR	473.7	1.39
CA-CX	350.8	1.486
CA-CZ	404.1	1.44
CA-N*	440.2	1.37
CA-NA	470.3	1.35
CA-NB	483.1	1.342
CA-NE	361.8	1.431
CA-NF	361.8	1.431
CA-NH	449	1.364
CA-NO	322.6	1.468
CA-OS	364	1.38
CA-S	307.7	1.7
CA-SY	247.7	1.784
CB-CM	473.7	1.39
CB-CV	473.7	1.39
CB-NA	438.8	1.371
CB-OS	364	1.38
CB-SY	248.9	1.782
C-C*	346.5	1.49
CC-C	346.5	1.49
CC-C*	473.7	1.39
CC-CM	473.7	1.39
CC-N	426	1.38
CC-OS	364	1.38
CD-N	426	1.38
CD-NC	494.6	1.335
CG-N1	994.7	1.143
CM-Cl	322.8	1.729
CM-N2	599.8	1.279
CM-NA	440.2	1.37
CM-NC	431.6	1.376
CM-NH	449	1.364
CM-NT	486.3	1.34
CR-C*	473.7	1.39
CR-N	426	1.38
CR-N*	440.2	1.37
CR-S	307.7	1.7
C-S	231.5	1.81
CT-CX	322.5	1.514
CT-NA	334.7	1.456
CT-NH	332.7	1.458
CT-S6	254	1.774
CT-SY	248.9	1.782
CV-CA	473.7	1.39
CV-CT	330.3	1.51
CV-OS	364	1.38
CX-CX	337.3	1.499
CX-HC	345.8	1.086
H-NH	401.2	1.014
N*-C*	440.2	1.37
N*-NA	453.3	1.401
NA-NC	535.7	1.35
NB-N*	535.7	1.35
NB-NB	486.8	1.379
NB-SY	439.8	1.555
NC-NC	550.2	1.342

NE-NF	738.6	1.257
NH-SY	324.2	1.664
N-NC	532.1	1.352
N-OH	395.4	1.41
NO-O	761.2	1.219
NT-S6	353.8	1.632
NT-SY	324.2	1.664
O-S	493	1.466
O-S6	541.1	1.436
O-SY	493	1.466
S-C*	307.7	1.7
S-CB	307.7	1.7
S-CC	307.7	1.7
ANGLE		
C*-C-N	70.2	111.86
CA-C-CT	63.33	115.23
CA-C-NA	62.8	119.53
CA-C-NC	67.19	112.17
CA-N-H	47.6	114.59
CA-S-C*	66.5	95.23
CA-S-CA	62.4	99.92
CB-C-N	70.2	111.86
CC-C-N	68	112.68
CC-C-O	67.1	126.4
C-C-CT	61.7	116.69
CC-N-H	47.5	121.52
CD-N-H	47.5	121.52
CD-N-NC	67.9	117.57
CM-C-CA	64.1	114.03
CM-C-N	68	112.68
C-N-C	62.4	128.18
C-N-C*	64.3	123.71
C-N-CA	64.3	123.71
C-N-CC	65.2	124.19
C-N-CD	65.2	124.19
C-N-CR	65.2	124.19
C-N-NC	68.6	120.31
C-N-OH	69.5	113.39
CR-N-H	47.5	121.52
CR-S-CA	66.5	95.23
CT-N-CA	62.4	121.15
CT-N-NC	63.3	121.17
CT-S-CA	61.3	100.62
H-N-OH	50.7	109.21
N-C-C	61.76	116.17
N-C-CA	68	112.68
N-C-N	71.3	114.54
N-C-OS	75.74	111.02
N-C-S	55.64	113.78
O-C-C*	67.1	126.4
O-C-CA	67.1	126.4
O-C-S	62.8	122.98
OS-C-CA	67.66	110.77
C*-C*-C	62	122.13
C*-C*-C*	63	120
C*-C*-CC	63	120
C*-C*-Cl	57.1	119.36
C*-C*-HA	50	120
C*-C*-OS	66.1	122.03
C*-CA-N*	68.7	111.2
C*-CA-NA	70.2	118.34

C*-CA-SY	62	120.35
C*-CC-C	62	122.13
C*-CC-N	70.7	115.52
C*-CC-NA	68.7	111.2
C*-CC-NB	72.2	106.8
C*-CM-CM	63	120
C*-CM-NA	68.7	111.2
C*-CR-N	70.7	115.52
C*-CR-N*	68.7	111.2
C*-CT-CA	64.3	110.36
C*-CT-H1	47	110.15
C*-CT-NT	66.5	111.47
C*-CV-CT	61.4	122.59
C*-CV-NB	70.2	118.34
C*-OS-CB	62.7	120.91
C*-S-CB	66.5	95.23
C2-N2-CT	66.1	115.3
C2-N3-CT	62.8	110.64
C2-N3-H	46.4	111.36
C2-NH-CA	65.5	123.66
C2-NH-CT	63.2	123.71
C2-NH-H	49	117.94
C2-OS-CT	64.2	112.09
CA-C*-C*	66	120.1
CA-C*-CT	63.8	120.63
CA-C*-S	61.1	120.79
CA-CA-C*	66	120.1
CA-CA-CC	63	120
CA-CA-CG	65.9	120.05
CA-CA-CR	63	120
CA-CA-CX	64.8	119.68
CA-CA-N*	70.2	118.34
CA-CA-NB	69.2	122.63
CA-CA-NC	70.1	119.72
CA-CA-NE	67.7	119.88
CA-CA-NF	67.7	119.88
CA-CA-NH	69.3	120.13
CA-CA-NO	66.9	119.54
CA-CA-S	62	119.93
CA-CA-SY	62	120.35
CA-CB-CV	63	120
CA-CB-NA	72.9	109.42
CA-CB-OS	66.1	122.03
CA-CB-SY	61.6	119.89
CA-CC-CT	61.4	122.59
CA-CC-NB	70.2	115.05
CA-CC-OS	66.1	122.03
CA-CG-N1	58.8	180
CA-CM-C	62	122.13
CA-CM-CA	63	120
CA-CM-Cl	58.4	119.9
CA-CM-CT	61.4	122.59
CA-CM-N2	68.5	120.72
CA-CM-NC	70.1	119.72
CA-CM-NH	69.3	120.13
CA-CR-NA	68.7	111.2
CA-CR-NB	70.1	119.72
CA-CT-CA	64.3	110.36
CA-CT-NH	66.5	111.47
CA-CT-NT	66.5	111.47
CA-CT-OS	67.17	110.49
CA-CX-CA	63.6	112.47

CA-CZ-CZ	56.9	180
CA-N*-C	70	125.2
CA-N*-NA	66.3	123.76
CA-N*-NB	69.3	117.85
CA-NA-CA	67.1	119.8
CA-NA-CB	68.5	113.15
CA-NA-H	48.2	122.77
CA-NA-N*	66.3	123.76
CA-NA-NC	69.3	117.85
CA-NB-CB	68.6	115.86
CA-NB-NB	71.4	109.51
CA-NC-CA	68.6	115.86
CA-NC-CD	71.1	108.82
CA-NC-N	73.8	104.69
CA-NC-N*	70.04	109.6
CA-NC-NA	74.1	104.16
CA-NC-NC	69.4	118.89
CA-NE-NF	69.9	114.72
CA-NF-NE	69.9	114.72
CA-NH-CA	64.3	127.46
CA-NH-CM	64.3	127.46
CA-NH-H	49.6	113.79
CA-NH-SY	64	117.74
CA-NO-O	68.7	118.1
CA-OS-CA	62.7	120.91
CA-S-C*	66.5	95.23
CA-S-CB	66.5	95.23
CA-S-CC	66.5	95.23
CA-SY-CA	61.1	102.78
CA-SY-NH	65.2	100.83
CA-SY-NT	65.2	100.83
CA-SY-O	66.4	107.25
CB-C*-C*	63	120
CB-C*-CA	63	120
CB-C*-CC	63	120
CB-C*-HA	48.4	122.89
CB-CA-OS	66.1	122.03
CB-CB-C*	68.2	114.19
CB-CB-CM	63	120
CB-CB-CV	63	120
CB-CB-OS	66.1	122.03
CB-CB-SY	61.6	119.89
CB-CM-C	62	122.13
CB-CM-CA	63	120
CB-CM-NA	68.7	111.2
CB-CM-OS	66.1	122.03
CB-CV-NB	71	114.98
CB-CV-OS	66.1	122.03
CB-N*-C*	67.7	110.55
CB-N*-CA	67.7	110.55
CB-SY-NB	65.2	100.83
CB-SY-NH	65.2	100.83
CB-SY-O	66.4	107.25
C-C*-C*	64.6	120.14
C-C*-CC	62	122.13
C-C*-S	73.99	117.58
C-CA-S	62	119.93
CC-C*-CM	63	120
CC-CM-CA	63	120
C-CM-OS	66.17	116.86
CC-NB-CC	68.6	110.19
CC-OS-CA	62.7	120.91

C-CT-C	63.7	112.95
C-CT-C*	64.16	110.97
C-CT-CA	64.16	110.97
C-CT-H2	50	109.5
C-CT-NH	68.6	105.72
C-CT-OH	66.94	111.33
C-CT-S	65.98	110.19
CG-CA-NC	67	120
CK-N*-C*	67.7	110.55
CK-NB-CC	68.5	113.15
CM-CA-CA	63	120
CM-CA-CB	63	120
CM-CA-CM	63	120
CM-CA-HA	50	120
CM-CA-N	68	119.89
CM-CA-NH	69.3	120.13
CM-CC-C*	63	120
CM-CC-NB	72.9	109.42
CM-CM-CM	63	120
CM-CM-NH	69.3	120.13
CM-CT-CA	64.3	110.36
CM-CT-NH	66.6	109.78
CM-NA-CA	67.7	110.55
CM-NC-CA	68.6	115.86
CM-NH-CA	64.3	127.46
CM-NH-H	48.9	117.16
CN-CB-CM	63	120
CN-CB-NB	70.1	119.72
CN-NA-CC	67.7	110.55
CR-C*-CV	63	120
CR-C*-HA	50	120
CR-N*-CA	67.7	110.55
CR-N*-CT	62.7	123.21
CR-N*-NB	70.2	113.02
CR-NA-CB	67.7	110.55
CR-NA-CN	67.7	110.55
CR-NB-NB	71.4	109.51
C-S-CT	61.2	98.71
CT-C*-C*	61.4	122.59
CT-C2-NH	66.7	118.59
CT-CA-C*	63.8	120.63
CT-CA-CB	61.4	122.59
CT-CA-CC	61.4	122.59
CT-CA-NA	65.09	115.06
CT-CA-NB	64.46	118.4
CT-CA-NH	66.7	118.59
CT-CA-S	75.22	115.97
CT-CD-N	66.4	121.96
CT-CD-NC	66.4	121.96
CT-CM-CA	61.4	122.59
CT-CT-NA	65.8	112.59
CT-CT-NH	66.6	109.78
CT-CT-S6	62.4	111.9
CT-CT-SY	43.7	108.09
CT-CV-NB	64.46	118.4
CT-CX-CX	61.7	120.67
CT-CX-HC	45.8	117.92
CT-N*-C*	62.7	123.21
CT-N*-CA	63.1	124.36
CT-N*-NA	65.7	120.46
CT-N2-CM	60.6	123.2
CT-NA-CA	63.1	124.36

CT-NA-CB	62.6	125.09
CT-NH-CA	64.6	117.77
CT-NH-CM	63.7	121.18
CT-NH-H	46.5	114.95
CT-NT-CM	16.9	116.39
CT-NT-S	43	113.89
CT-NT-S6	63.9	115.81
CT-NT-SY	63.9	115.81
CT-OS-CA	61.74	117.87
CT-S6-NT	65.2	102.92
CT-S6-O	66.6	108.32
CT-SY-CA	60.9	103.17
CT-SY-O	66	108.48
CV-CA-CA	63	120
CV-CA-H4	50	120
CV-CA-NB	66.9	126.21
CV-CA-S	61.1	120.79
CV-CT-CT	63	114
CV-NB-N*	70.04	109.6
CV-NB-NB	69.4	118.89
CV-NB-SY	62.9	114.81
CV-OS-CT	61.74	117.87
CX-CA-NC	65.8	121.44
CX-CT-H1	46.7	111.35
CX-CT-N	65.9	112.13
CX-CX-CA	62.5	114.61
CX-CX-CX	85.3	63.75
CX-CX-HC	45.8	117.92
CZ-CA-CA	63	120
F-CT-C	63.95	110.33
F-CT-CA	64.12	109.73
H1-CT-CA	50	109.5
H1-CT-NA	49.9	109.5
H1-CT-NH	49.7	109.96
H1-CT-S6	43.8	108.2
H1-CT-SY	43.7	108.09
H4-CA-C*	50	120
H4-CA-CM	50	120
H4-CA-NA	51.9	114.65
H4-CA-NC	51.8	115.94
H4-CV-CA	50	120
H5-CA-NA	50.7	122.11
H5-CA-NC	50.7	122.11
HA-C*-C*	50	120
HA-C*-CA	50	120
HA-C*-CB	50	120
HA-C*-CC	50	120
HA-C*-CV	50	120
HA-CA-CM	50	120
HC-CX-HC	38.6	114.47
H-NA-CB	46.5	125.33
H-NA-CM	46.5	125.33
H-NA-N*	50	119.61
H-NA-NC	50	119.61
H-NH-H	40.1	114.43
H-NH-SY	45.2	109.1
H-NT-S6	45.4	112.52
H-NT-SY	45.2	109.1
HP-CT-C*	50	109.5
HP-CT-CA	50	109.5
HP-CT-CM	50	109.5
N*-C*-C	65.77	116.66

N*-C*-C*	68.7	111.2
N*-C*-CC	68.7	111.2
N*-CA-CM	68.7	111.2
N*-CA-CT	65.09	115.06
N*-CA-NC	70.2	128.56
N*-CB-CA	68.7	111.2
N*-CT-CA	66.3	111.92
N2-C2-OS	74.6	118.96
N2-CM-NH	72.6	124.27
N3-C2-N2	67.1	121.24
N3-C2-OS	51.7	116.11
N3-CT-C*	65.68	111.84
N3-CT-CA	65.68	111.84
N3-CT-CM	65.68	111.84
NA-CA-CA	68.7	111.2
NA-CA-NA	74.5	113.32
NA-CA-NC	70.2	128.56
NA-CA-NH	72.6	118.96
NA-CA-OH	69.8	119.94
NA-CB-CB	68.7	111.2
NA-CB-NA	74.5	113.32
NA-CB-NC	69.7	125.32
NA-CC-C	65.77	116.66
NA-CM-CM	68.7	111.2
NA-CM-NH	72.4	120.11
NA-CM-OS	73.53	113.53
NB-CA-NB	71.2	125.84
NB-CA-SY	62.8	120.01
NB-CB-CM	70.2	115.05
NB-CC-OS	71.64	116.86
NB-CR-S	66.5	114.51
NB-CV-CA	70.1	119.72
NB-CV-NB	71.2	125.84
NB-CV-OS	71.64	116.86
NB-N*-CT	65.7	120.46
NB-NB-NB	70.4	121.04
NB-SY-O	68.9	107.06
N-CA-NC	69.7	123.86
N-CA-NH	71.3	116.16
NC-CA-C	64.99	120
NC-CA-CT	64.46	118.4
NC-CA-NC	71.2	125.84
NC-CA-NH	73.9	111.94
NC-CB-C*	70.1	119.72
NC-CM-NH	73.9	111.94
N-CC-S	67.4	109.31
N-CD-NC	70.9	123.86
N-CM-NH	72.2	117.01
N-CR-N*	70.9	123.86
N-CR-NB	69.7	123.86
N-CR-S	67.4	109.31
N-CT-CA	66.18	112.06
NH-C2-NH	72.9	117.5
NH-CA-NH	71.4	120.98
NH-CM-NH	71.4	120.98
NH-SY-O	70.9	108.46
NT-CM-CA	69.5	121.19
NT-CM-NT	46.6	122.38
NT-CT-C	80	111.2
NT-CT-H2	50	109.5
NT-CT-NT	69.3	110.48
NT-CT-S	65.06	109.64

NT-S6-NT	69.5	104.28		
NT-S6-O	72.9	106.8		
NT-SY-O	70.9	108.46		
OH-CT-CA	67.12	110.72		
O-NO-O	76.4	127.55		
O-S6-O	74.6	119.82		
OS-CA-CA	66.1	122.03		
OS-CA-CC	66.1	122.03		
OS-CA-CT	65.53	115.27		
OS-CA-NC	71.64	116.86		
O-SY-O	72.4	121.88		
S-C*-C*	62	119.93		
S-CA-C*	62	119.93		
S-CA-NB	71.16	117.58		
S-CA-S	65.5	115.15		
S-CA-SY	62.2	122.3		
S-CB-CA	61.1	120.79		
S-CB-CB	62	119.93		
S-CB-SY	62.2	122.3		
S-CC-C*	61.1	120.79		
S-CT-H2	50	109.5		
DIHEDRAL				
X-C*-C*-X	1	3.625	180	2
X-C*-N*-X	1	0.3	180	2
X-C*-S-X	2	0.8	180	2
X-C*-OS-X	1	0.9	180	2
X-C2-N2-X	2	8.3	180	2
X-C2-N3-X	6	0	180	3
X-C2-NH-X	4	2.7	180	2
X-C2-OS-X	2	2.1	180	2
X-CA-C*-X	1	3.625	180	2
X-CA-CA-X	4	14.5	180	2
X-CA-CC-X	1	3.625	180	2
X-CA-CR-X	1	3.625	180	2
X-CA-CV-X	1	3.625	180	2
X-CA-CZ-X	1	0	180	2
X-CA-NA-X	4	1.2	180	2
X-CA-NC-X	2	9.6	180	2
X-CA-NE-X	2	0	180	3
X-CA-NF-X	2	0	180	3
X-CA-NH-X	4	4.2	180	2
X-CA-NO-X	4	2.4	180	2
X-CA-N-X	4	1.8	180	2
X-CA-OS-X	2	1.8	180	2
X-CA-S-X	2	0.8	180	2
X-CA-SY-X	6	7.8	180	2
X-CB-CM-X	1	3.625	180	2
X-CB-S-X	2	0.8	180	2
X-CB-SY-X	6	7.6	180	2
X-C-C*-X	1	3.625	180	2
X-CC-C*-X	1	3.625	180	2
X-CM-CC-X	1	3.625	180	2
X-CC-OS-X	1	0.9	180	2
X-CC-S-X	2	2.2	180	2
X-CC-C-X	1	3.625	180	2
X-C-CT-X	6	0	180	2
X-C-S-X	1	3.1	180	2
X-CD-NC-X	2	9.5	180	2
X-CG-CA-X	2	0	180	2
X-CM-N2-X	2	0	180	3
X-CM-NC-X	2	9.5	180	2

X-CM-NH-X	4	4.2	180	2
X-CM-NT-X	4	1.2	180	2
X-CR-C*-X	4	16	180	2
X-CR-N*-X	4	6.8	180	2
X-CR-N-X	4	2.6	180	2
X-CR-S-X	2	2.2	180	2
X-CT-CA-X	6	0	0	3
X-CT-CX-X	9	1.4	0	3
X-CV-CT-X	1	0	0	2
X-CV-OS-X	1	0.9	180	2
X-CT-NA-X	6	0	0	2
X-CT-NH-X	6	0	0	2
X-CT-S6-X	9	1.3	0	3
X-CT-SY-X	9	1.3	0	3
X-CX-CA-X	6	0	0	2
X-CX-CX-X	9	1.4	0	3
X-CZ-CZ-X	1	0	180	2
X-NA-CB-X	1	0.3	180	2
X-NA-CM-X	1	0.3	180	2
X-NA-N*-X	4	3.6	0	2
X-NA-NC-X	2	9.6	180	2
X-NB-N*-X	2	9.6	180	2
X-NB-NB-X	1	4	180	2
X-NB-SY-X	3	1.5	180	-3
X-NB-SY-X	3	20.4	180	1
X-N-CC-X	4	6.6	180	2
X-N-CD-X	4	6.6	180	2
X-NE-NF-X	1	3	180	-2
X-NE-NF-X	1	2.8	0	1
X-NH-SY-X	6	0.6	180	2
X-N-NC-X	2	9.6	180	2
X-NT-S6-X	6	18.8	0	2
X-NT-SY-X	6	18.8	0	2

IMPROPER

C*-C-C*-CC	1.1	180	2
C*-C-C*-N*	1.1	180	2
CA-C-CA-S	1.1	180	2
C*-C-CC-NA	1.1	180	2
C-C-N-CA	1.1	180	2
O-C-N-N	1.1	180	2
C*-C*-C*-Cl	1.1	180	2
C*-C*-C*-OS	1.1	180	2
C*-C*-C*-S	1.1	180	2
CA-C2-NH-H	1.1	180	2
C*-CA-C*-CT	1.1	180	2
C*-CA-C*-HA	1.1	180	2
Br-CA-CA-CA	1.1	180	2
CA-CA-CA-CC	1.1	180	2
CA-CA-CA-CG	1.1	180	2
CA-CA-CA-CM	1.1	180	2
CA-CA-CA-CR	1.1	180	2
CA-CA-CA-CX	1.1	180	2
CA-CA-CA-CZ	1.1	180	2
CA-CA-CA-F	1.1	180	2
CA-CA-CA-NA	1.1	180	2
CA-CA-CA-NC	1.1	180	2
CA-CA-CA-NE	1.1	180	2
CA-CA-CA-NF	1.1	180	2
CA-CA-CA-NH	1.1	180	2
CA-CA-CA-NO	1.1	180	2
CA-CA-CA-S	1.1	180	2

CA-CA-CA-SY	1.1	180	2
NC-CA-CA-C	1.1	180	2
OH-CA-CA-CA	1.1	180	2
C*-CA-CB-CN	1.1	180	2
C-CA-CM-CB	1.1	180	2
CA-CA-CM-CC	1.1	180	2
CA-CA-CM-Cl	1.1	180	2
CA-CA-CM-CM	1.1	180	2
CA-CA-CM-CT	1.1	180	2
NC-CA-CT-CA	1.1	180	2
NC-CA-H4-CA	1.1	180	2
S-CA-H4-C*	1.1	180	2
C-CA-N-CT	1.1	180	2
CA-CA-NH-H	1.1	180	2
CA-CA-NH-SY	1.1	180	2
NC-CA-NH-CA	1.1	180	2
S-CA-S-C*	1.1	180	2
C*-CB-C*-CT	1.1	180	2
CA-CB-C*-HA	1.1	180	2
CA-CB-CA-CM	1.1	180	2
C-CB-CB-NB	1.1	180	2
C*-CB-CB-NC	1.1	180	2
CA-CB-CB-CV	1.1	180	2
CA-CB-CB-NA	1.1	180	2
CA-CB-CB-NB	1.1	180	2
CA-CB-CB-OS	1.1	180	2
CA-CB-CB-S	1.1	180	2
CA-CB-CB-SY	1.1	180	2
CA-CB-CN-NA	1.1	180	2
CA-CB-NA-CT	1.1	180	2
CB-CC-C*-HA	1.1	180	2
CA-CG-CA-NC	1.1	180	2
C*-CK-N*-CT	1.1	180	2
CC-CM-C*-N*	1.1	180	2
CA-CM-CA-N	1.1	180	2
CM-CM-CA-N	1.1	180	2
CM-CM-CA-N*	1.1	180	2
CB-CM-CB-NB	1.1	180	2
C*-CM-CC-NB	1.1	180	2
C-CM-CM-OS	1.1	180	2
C*-CM-CM-NA	1.1	180	2
CA-CM-CM-CT	1.1	180	2
CM-CM-CM-NH	1.1	180	2
CA-CM-NH-H	1.1	180	2
CM-CN-CB-NB	1.1	180	2
CA-CR-N*-NB	1.1	180	2
CB-CT-C*-CW	1.1	180	2
C*-CT-CA-S	1.1	180	2
CA-CT-CA-NH	1.1	180	2
CC-CT-CA-OS	1.1	180	2
CA-CT-CC-NB	1.1	180	2
C*-CT-CV-NB	1.1	180	2
C-CT-N-NC	1.1	180	2
CA-CT-N*-NA	1.1	180	2
CR-CT-N*-NB	1.1	180	2
CA-CT-NH-CT	1.1	180	2
CA-CT-NH-H	1.1	180	2
CR-CV-C*-HA	1.1	180	2
CA-CX-CA-NC	1.1	180	2
CA-H-NA-N*	1.1	180	2
CA-H-NA-NC	1.1	180	2
CA-H-NH-H	1.1	180	2

CA-H-NH-SY	1.1	180	2
CM-H-NH-H	1.1	180	2
CM-H-NH-NA	1.1	180	2
C*-H4-CA-N*	1.1	180	2
C*-H4-CA-NA	1.1	180	2
C*-H4-CA-S	1.1	180	2
CA-H4-CA-NC	1.1	180	2
CA-H4-CA-S	1.1	180	2
CM-H4-CA-NC	1.1	180	2
CV-H4-CA-S	1.1	180	2
C*-H4-CW-NA	1.1	180	2
CA-N-CA-NC	1.1	180	2
NH-N-CA-NC	1.1	180	2
C*-N-CC-S	1.1	180	2
CT-N-CD-NC	1.1	180	2
CA-N*-CA-NC	1.1	180	2
CB-N*-CB-NC	1.1	180	2
H5-N*-CK-NB	1.1	180	2
C*-N*-CR-N	1.1	180	2
CA-N2-CM-NH	1.1	180	2
N2-N3-C2-OS	1.1	180	2
CA-NA-CA-NC	1.1	180	2
CA-NA-CA-OH	1.1	180	2
H4-NA-CA-NC	1.1	180	2
NH-NA-CA-NA	1.1	180	2
NH-NA-CA-NC	1.1	180	2
CB-NA-CB-NC	1.1	180	2
CB-NA-CM-OS	1.1	180	2
CA-NA-CR-NB	1.1	180	2
H5-NA-CR-NB	1.1	180	2
CT-NB-CA-S	1.1	180	2
CA-NB-CC-OS	1.1	180	2
N-NB-CR-S	1.1	180	2
CB-NB-CV-OS	1.1	180	2
CA-NC-CA-OS	1.1	180	2
H5-NC-CA-NC	1.1	180	2
CA-NE-NE-NF	1.1	180	2
CA-NE-NF-NF	1.1	180	2
NH-NH-C2-NH	1.1	180	2
CA-NH-CA-NA	1.1	180	2
CA-NH-CA-NC	1.1	180	2
CA-NH-CA-NH	1.1	180	2
CM-NH-CA-NC	1.1	180	2
CA-NH-CM-NC	1.1	180	2
CA-NH-CM-NH	1.1	180	2
CM-NH-CM-NA	1.1	180	2
NH-NH-CM-NH	1.1	180	2
CA-O-C-OS	1.1	180	2
CT-O-C-OS	1.1	180	2
N-O-C-OS	1.1	180	2
N-O-C-S	1.1	180	2
CA-O-NO-O	1.1	180	2
C*-S-CA-SY	1.1	180	2
NB-S-CA-SY	1.1	180	2
CB-S-CB-SY	1.1	180	2

NONBOND

	C2	1.908	0.086
	CG	1.908	0.086
	CX	1.908	0.086
	N1	1.824	0.17
	NE	1.824	0.17

NF	1.824	0.17
NH	1.824	0.17
NO	1.824	0.17
S6	2	0.25
SY	2	0.25
