# Supplementary material for: Preference of Small Molecules for Local Minimum Conformations when Binding to Proteins
Source: PLoS One. 2007 Sep 5;2(9):e820. doi: 10.1371/journal.pone.0000820 (PMC1959118; doi:10.1371/journal.pone.0000820)
Supplement: Table S5 — The AMBER atom types and RESP charges of the 100 protein-bound ligands. The atom labels are shown in Figures S7, S8, S9, S10, S11, S12 and S13. (0.21 MB PDF) [file pone.0000820.s005.pdf]

Table S5. The AMBER atom types and RESP charges of the 100 protein-bound ligands. The atom labels are shown in Figures S7–S13.

13gs

N1	NA	-0.009
H1	H	0.300
C1	CA	-0.151
H2	H4	0.218
C2	CA	-0.047
H3	HA	0.180
C3	CA	0.021
H14	HA	0.160
C4	CA	-0.087
H4	HA	0.151
C5	CA	0.135
N2	NH	-0.530
H5	H	0.390
S1	SY	0.929
O1	O	-0.485
O2	O	-0.485
C6	CA	-0.005
C9	CA	-0.168
H8	HA	0.171
C10	CA	-0.212
H9	HA	0.175
C7	CA	-0.168
H6	HA	0.171
C8	CA	-0.212
H7	HA	0.175
C11	CA	0.370
N3	NE	-0.343
N4	NF	0.017
C12	CA	0.083
C13	CA	-0.142
H10	HA	0.012
C14	CA	0.031
C15	C	0.752
O3	O	-0.718
O4	O	-0.718
C16	CA	0.240
O5	OH	-0.499
H11	HO	0.417
C17	CA	-0.363
H12	HA	0.178
C18	CA	-0.059
H13	HA	0.125

1a42

C1	CT	-0.058
H1	HC	0.026
H2	HC	0.026
H3	HC	0.026
C2	CT	0.176
H4	H1	0.026
H5	H1	0.026
O1	OS	-0.450
C3	CT	0.134
H6	H1	0.075
H7	H1	0.075
C4	CT	-0.045
H8	H1	0.113
H9	H1	0.113
N1	NT	-0.271
S1	SY	0.759
O2	O	-0.439
O3	O	-0.439
C5	CT	-0.218
H10	H1	0.150
H11	H1	0.150
C10	CT	0.000
H14	HP	0.123
N3	N3	-0.008
H15	H	0.217
C11	CT	0.035
H16	HP	0.074
H17	HP	0.074
C12	CT	-0.128
H18	HC	0.065
H19	HC	0.065
H20	HC	0.065
H21	H	0.217
C9	C*	-0.027
C8	C*	-0.112
H13	HA	0.169
C6	CA	-0.089
S2	S	0.148
C7	CA	-0.187
S3	SY	1.168
O4	O	-0.612
O5	O	-0.612
N2	NT	-0.979
H12	H	0.379

1a4k

C1	CT	-0.405
H1	HC	0.114
H2	HC	0.114
H3	HC	0.114
C2	C	0.687
O1	O	-0.547
N1	N	-0.429
H4	H	0.249
C3	CA	0.107
C6	CA	-0.127
H7	HA	0.141
C7	CA	-0.234
H8	HA	0.179
C4	CA	-0.127
H6	HA	0.141
C5	CA	-0.234
H5	HA	0.179
C8	CA	0.149
N2	N	-0.039
C10	C	0.403
O3	O	-0.518
C9	C	0.386
O2	O	-0.488
C17	CT	0.004
H19	HC	0.074
C11	CT	-0.144
H18	HC	0.077
C12	CT	0.102
C15	CT	-0.116
C16	CT	-0.164
H13	HC	0.082
H14	HC	0.082
H15	HC	0.034
H16	HC	0.034
H17	HC	0.010
C13	CT	-0.116
H11	HC	0.034
H12	HC	0.034
C14	CT	-0.164
H9	HC	0.082
H10	HC	0.082
C18	CT	0.409
N3	N	-0.849
H20	H	0.383
C19	C	0.905
O4	O	-0.604
O5	OS	-0.452
C20	CT	0.062
H21	H1	0.029
H22	H1	0.029
C21	C	0.855
O7	O	-0.807
O6	O	-0.807

1a8t

N1	NB	-0.652
N2	NB	-0.192
N3	NB	-0.192
N4	NB	-0.652
C1	CV	1.277
C2	CA	-0.616
C7	CA	-0.021
C6	CA	-0.075
C5	CA	-0.247
C4	CA	-0.144
H1	HA	0.107
H2	HA	0.135
H3	HA	0.098
H4	HA	0.076
C3	CA	0.305
C8	CA	-0.149
C13	CA	-0.098
C12	CA	-0.164
H7	HA	0.139
H8	HA	0.123
C9	CA	-0.098
H5	HA	0.123
C10	CA	-0.164
H6	HA	0.139
C11	CA	-0.025
C14	CT	0.011
H9	H1	0.060
H10	H1	0.060
N5	N*	0.016
C15	CA	0.228
C16	CT	-0.160
C17	CT	0.009
C18	CT	0.050
C19	CT	0.039
H17	HC	-0.024
H18	HC	-0.024
H19	HC	-0.024
H15	HC	-0.008
H16	HC	-0.008
H13	HC	-0.015
H14	HC	-0.015
H11	HC	0.136
H12	HC	0.136
N6	NC	-0.531
C20	CA	0.307
C21	CM	-0.267
H20	HA	0.166
C22	CA	-0.155
H21	HA	0.172
C23	CA	0.288
O1	OH	-0.619
H22	HO	0.444
C24	CA	-0.313
H23	HA	0.178
C25	CA	-0.047
C26	C	0.430
O2	O	-0.556

1afq

C5	CT	-0.099
H7	HC	0.034
H8	HC	0.034
H9	HC	0.034
C4	CT	0.107
C6	CT	-0.099
H10	HC	0.034
H11	HC	0.034
H12	HC	0.034
H6	HC	0.065
C3	CT	-0.309
H4	HC	0.140
H5	HC	0.140
C1	CT	-0.005
N1	N3	-0.421
H1	H	0.339
H2	H	0.339
H13	H	0.339
H3	HP	0.145
C2	C	0.455
O1	O	-0.476
N2	N	-0.521
H14	H	0.324
C7	CT	0.109
C9	CT	-0.067
C10	CA	-0.026
C11	CA	-0.127
C13	CA	-0.165
C15	CA	-0.092
C14	CA	-0.165
C12	CA	-0.127
H19	HA	0.133
H21	HA	0.155
H22	HA	0.144
H20	HA	0.155
H18	HA	0.133
H16	HC	0.060
H17	HC	0.060
H15	H1	0.098
C8	C	0.576
O2	O	-0.600
N3	N	-0.463
H23	H	0.311
C16	CT	-0.051
H24	H1	0.113
H25	H1	0.113
C17	CA	0.031
C18	CA	-0.174
H26	HA	0.161
C19	CA	-0.209
H27	HA	0.174
C20	CA	0.310
F1	F	-0.187
C21	CA	-0.209
H28	HA	0.174
C22	CA	-0.174
H29	HA	0.161

1aoe

N5	NH	-0.975
H7	H	0.463
H8	H	0.463
C4	CA	0.766
N2	NA	-0.330
H20	H	0.334
N1	NC	-0.607
C3	CM	0.559
N3	NH	-0.764
H1	H	0.407
H2	H	0.407
C2	CA	0.033
C1	CA	0.040
C5	CA	-0.271
H3	HA	0.197
C6	CA	-0.136
H4	HA	0.185
C7	CB	0.070
C8	CB	-0.007
C9	C*	-0.430
H5	HA	0.204
C10	CA	-0.035
H6	H4	0.202
N4	N*	0.085
C11	CT	-0.011
C14	CT	-0.150
C15	CT	-0.046
H17	HC	0.024
H18	HC	0.024
H19	HC	0.024
H15	HC	0.074
H16	HC	0.074
H9	H1	0.103
C12	CT	-0.150
H10	HC	0.074

H11	HC	0.074
C13	CT	-0.046
H12	HC	0.024
H13	HC	0.024
H14	HC	0.024

1atl

C1	CT	-0.215
H1	HC	0.029
H2	HC	0.029
H3	HC	0.029
C2	CT	0.350
C3	CT	-0.215
H4	HC	0.029
H5	HC	0.029
H6	HC	0.029
H7	HC	-0.003
C4	CT	0.063
H8	HC	-0.049
H9	HC	-0.049
C5	CT	0.028
C6	CT	-0.197
S1	SH	-0.421
H10	HS	0.237
H11	H1	0.136
H12	H1	0.136
H13	HC	0.037
C7	C	0.279
O1	O	-0.489
N1	N	-0.424
H24	H	0.322
C8	CT	0.042
C9	C	0.822
O2	O	-0.784
O3	O	-0.784
H14	H1	0.053
C10	CT	0.003
H15	HC	0.007
H16	HC	0.007
C11	CA	0.047
C12	CA	-0.240
H18	HA	0.209
C13	CA	-0.215
H17	HA	0.127
C16	CA	0.302
O4	OS	-0.346
C17	CT	0.008
H21	H1	0.054
H22	H1	0.054
H23	H1	0.054
C15	CA	-0.215
H20	HA	0.127
C14	CA	-0.240
H19	HA	0.209

1azm

C4	CT	-0.258
H1	HC	0.078
H2	HC	0.078
H3	HC	0.078
C3	C	0.687
O3	O	-0.554
N4	N	-0.550
H5	H	0.281
C2	CR	0.510
S2	S	-0.082
N2	NB	-0.380
N3	NB	-0.358
C1	CA	0.289
S1	SY	1.017
O1	O	-0.621
O2	O	-0.621
N1	NT	-0.918
H4	H	0.323

1bnw

S2	S	-0.036
C1	CA	-0.029
S1	SY	1.125
N2	NT	-0.984
H9	H	0.349
O1	O	-0.627
O2	O	-0.627
C2	C*	-0.190
H1	HA	0.157
C3	C*	-0.045
H2	HA	0.140
C4	CA	-0.074
S3	SY	0.921
O3	O	-0.535
O4	O	-0.535
N1	NT	-0.576
H3	H	0.392
C5	CT	-0.005
H4	H1	0.119
H5	H1	0.119
C6	CA	-0.030
C7	C*	-0.025
H6	HA	0.153
C8	C*	-0.290
H7	HA	0.184
C9	CA	-0.188
H8	H4	0.207
S4	S	-0.068

1bqo

C1	CT	0.001
H1	H1	0.069
H2	H1	0.069
H3	H1	0.069
O1	OS	-0.338
C2	CA	0.295
C5	CA	-0.226
H6	HA	0.141
C6	CA	-0.103
H7	HA	0.140
C3	CA	-0.226
H5	HA	0.141
C4	CA	-0.103
H4	HA	0.140
C7	CA	0.000
S1	SY	0.806
O2	O	-0.529
O3	O	-0.529
N1	NT	-0.153
C13	CT	0.013
C14	C	0.574
N2	N	-0.093
O4	OH	-0.682
H18	H	0.218
O5	O	-0.656
H19	H2	0.110
C8	CT	-0.146
H16	H1	0.077
H17	H1	0.077
C9	CT	0.400
C11	CT	-0.228
H10	HC	0.037
H11	HC	0.037
H12	HC	0.037
C12	CT	-0.228
H13	HC	0.037
H14	HC	0.037
H15	HC	0.037
C10	CT	-0.146
H8	H1	0.077
H9	H1	0.077
N3	NT	-0.153
S2	SY	0.806
O6	O	-0.529
O7	O	-0.529
C15	CA	0.000
C21	CA	-0.103
C20	CA	-0.226
H25	HA	0.141
H26	HA	0.140
C16	CA	-0.103
H20	HA	0.140
C17	CA	-0.226
H21	HA	0.141
C18	CA	0.295
O8	OS	-0.338
C19	CT	0.001
H22	H1	0.069
H23	H1	0.069
H24	H1	0.069

1br6

N1	NC	-0.653
C2	CA	0.218
C1	C	0.752
O2	O	-0.577
N4	NC	-0.741
C4	CA	0.715
N3	NH	-0.913
H1	H	0.400
H2	H	0.400
N2	NA	-0.501
H11	H	0.352
C3	CA	0.342
N5	NC	-0.504
C6	CA	0.099
H10	H4	0.140
C5	CA	0.474
C8	CT	0.039
H3	H1	0.085
H4	H1	0.085
N6	NH	-0.857
H5	H	0.403
C9	CA	0.334
C11	CA	-0.244
C13	CA	-0.179
H8	HA	0.152
H9	HA	0.119
C10	CA	-0.244
H6	HA	0.119
C12	CA	-0.179
H7	HA	0.152
C14	CA	-0.015
C7	C	0.810
O3	O	-0.792
O1	O	-0.792

1cet

C1	CT	-0.080
H1	HC	0.069
H2	HC	0.069
H3	HC	0.069
C2	CT	-0.038
H4	HP	0.098
H5	HP	0.098
N1	N3	-0.075
C3	CT	-0.038
C4	CT	-0.080
H6	HC	0.069
H7	HC	0.069
H8	HC	0.069
H9	HP	0.098
H10	HP	0.098
H28	H	0.275
C5	CT	-0.083
H11	HP	0.107
H12	HP	0.107
C6	CT	-0.010
H13	HC	0.042
H14	HC	0.042
C7	CT	-0.051
H15	HC	0.027
H16	HC	0.027
C8	CT	0.038
C9	CT	-0.143
H17	HC	0.068
H18	HC	0.068
H19	HC	0.068
H20	H1	0.113
N2	NH	-0.236
H21	H	0.273
C10	CA	0.092
C11	CA	-0.149
H22	HA	0.134
C12	CA	-0.026
H23	H4	0.222
N3	NA	-0.213
H24	H	0.353
C13	CA	0.116
C14	CA	-0.139
H25	HA	0.164
C18	CA	-0.002
Cl1	Cl	0.018
C17	CA	0.004
H27	HA	0.157
C16	CA	-0.319
H26	HA	0.204
C15	CA	0.157

1cim

O1	O	-0.630
S1	SY	1.212
O2	O	-0.630
N1	NT	-1.000
H1	H	0.382
C1	CA	-0.105
S2	S	0.169
C2	C*	-0.205
H2	HA	0.165
C4	C*	-0.003
C3	CA	-0.217
S3	SY	0.990
O3	O	-0.528
O4	O	-0.528
C7	CT	-0.036
C8	CT	-0.206
H8	HC	0.087
H9	HC	0.087
H10	HC	0.087
H11	H1	0.063
C6	CT	-0.075
H6	HC	0.076
H7	HC	0.076
C5	CT	0.041
H5	HP	0.141
N2	N3	-0.422
H3	H	0.336
H4	H	0.336
H12	H	0.336

1d3p

C29	CT	-0.029
C30	CT	-0.029
C27	CT	-0.067
H25	HP	0.108
H26	HP	0.108
H31	HC	0.060
H32	HC	0.060
H29	HC	0.060
H30	HC	0.060
C28	CT	-0.067
H27	HP	0.108
H28	HP	0.108
N3	N3	0.067
H38	H	0.268
C32	CT	-0.192
H35	HP	0.134
H36	HP	0.134
C15	CT	-0.034
H9	H1	0.141
H10	H1	0.141
O1	OS	-0.431
C13	CA	0.650
N1	NC	-0.514
C14	CA	0.165
H8	H4	0.106
C12	CA	-0.413
H7	HA	0.191
C11	CA	-0.009
H6	HA	0.162

C10	CA	0.055
C7	C*	-0.180
S1	S	-0.090
C3	CB	0.030
C2	CA	-0.287
H1	HA	0.212
C1	CA	0.274
O3	OH	-0.560
H37	HO	0.433
C6	CA	-0.152
H3	HA	0.167
C5	CA	-0.208
H2	HA	0.150
C4	CB	0.068
C8	C*	0.013
C9	CT	-0.032
H4	HC	0.056
H5	HC	0.056
C17	CA	0.049
C18	CA	-0.161
C19	CA	-0.149
H13	HA	0.157
H12	HA	0.143
C16	CA	-0.161
H11	HA	0.143
C21	CA	-0.149
H14	HA	0.157
C20	CA	0.106
O2	OS	-0.325
C22	CT	0.020
H15	H1	0.098
H16	H1	0.098
C31	CT	-0.087
H33	HP	0.125
H34	HP	0.125
N2	N3	0.019
H39	H	0.272
C25	CT	-0.056
H21	HP	0.094
H22	HP	0.094
C24	CT	-0.021
H19	HC	0.061
H20	HC	0.061
C23	CT	-0.021
H17	HC	0.061
H18	HC	0.061
C26	CT	-0.056
H23	HP	0.094
H24	HP	0.094

1d4p

C1	CC	-0.278
H1	HA	0.182
C2	CA	0.109
C3	CA	-0.171
H2	HA	0.157
C4	CA	-0.095
C5	CA	0.695
N1	NH	-0.907
H3	H	0.362
H4	H	0.362
N2	NK	-0.869
H5	H	0.373
C6	CA	-0.102
H6	HA	0.131
C7	CA	-0.276
H7	HA	0.176
C8	CA	0.117
N3	NA	-0.318
H8	H	0.344
C9	CC	-0.027
C10	C	0.576
O1	O	-0.574
N4	N	-0.209
C13	CT	-0.034
H13	H1	0.060
H14	H1	0.060
C14	CT	-0.055
H15	HC	0.033
H16	HC	0.033
C11	CT	-0.034
H11	H1	0.060
H12	H1	0.060
C12	CT	-0.055
H9	HC	0.033
H10	HC	0.033
C15	CT	0.066
H17	HC	0.020
C16	CT	-0.104
H18	HC	0.049
H19	HC	0.049
C17	CA	0.034
C18	CA	-0.143
H20	HA	0.127
C19	CA	-0.173
H21	HA	0.143
C20	CA	-0.105
H22	HA	0.127
C21	CA	-0.173
H23	HA	0.143
C22	CA	-0.143
H24	HA	0.127

1d6v

C4	CA	-0.153
H3	HA	0.126
C3	CA	-0.145
H2	HA	0.132
C1	CA	-0.141
H1	HA	0.125
C2	CA	-0.145
H5	HA	0.132
C6	CA	-0.153
H4	HA	0.126
C5	CA	-0.006
C7	CT	0.014
C12	CT	-0.027
C11	CT	-0.057
H16	HC	0.023
H17	HC	0.023
H12	HC	0.023
H13	HC	0.023
H11	HC	0.038
C8	CT	-0.047
H14	HC	0.065
H15	HC	0.065
C9	CT	0.062
O1	OH	-0.656
H20	HO	0.424
H19	H1	0.056
C10	CT	-0.020
H18	HC	0.095
C13	CA	-0.001
C18	CA	-0.167
C17	CA	-0.112
H8	HA	0.138
H9	HA	0.123
C14	CA	-0.167
H6	HA	0.123
C15	CA	-0.112
H7	HA	0.138
C16	CA	-0.051
C19	C	0.536
O2	O	-0.630
N1	N	-0.306
H10	H	0.248
C20	CT	-0.054
H21	H1	0.054
H22	H1	0.054
C21	CT	0.005
H23	HC	-0.005
H24	HC	-0.005
C22	CT	-0.055
H25	HC	-0.012
H26	HC	-0.012
C23	C	0.825
O4	O	-0.779
O3	O	-0.779

1dib

O1	O	-0.626
C1	C	0.698
N1	N	-0.322
C2	CA	-0.002
C3	CA	0.251
O2	OH	-0.497
H20	HO	0.504
N2	NA	-0.233
H1	H	0.327
C4	CA	0.683
N3	NH	-0.943
H2	H	0.449
H3	H	0.449
N4	NC	-0.625
C5	CA	0.468
N5	NH	-0.385
H4	H	0.314
C6	CT	0.068
H5	H1	0.064
H6	H1	0.064
C7	CT	-0.007
H7	H1	0.086
C8	CT	-0.015
H8	H1	0.100
H9	H1	0.100
N6	N	-0.188
C9	CA	-0.003
C12	CA	-0.143
H12	HA	0.114
C13	CA	-0.089
H13	HA	0.142
C10	CA	-0.143
H11	HA	0.114
C11	CA	-0.089
H10	HA	0.142
C14	CA	-0.020
C15	C	0.468
O3	O	-0.618
N7	N	-0.216
H14	H	0.274
C16	CT	-0.051
C17	C	0.844
O4	O	-0.826
O5	O	-0.826
H15	H1	0.051
C18	CT	-0.039
H16	HC	0.019
H17	HC	0.019
C19	CT	-0.065
H18	HC	-0.020
H19	HC	-0.020
C20	C	0.776
O7	O	-0.788
O6	O	-0.788

1dlr

C1	CT	-0.011
H1	HC	0.044
H2	HC	0.044
H3	HC	0.044
C2	CA	-0.003
C3	CA	-0.046
C4	CA	0.497
N1	NH	-0.696
H4	H	0.384
H5	H	0.384
N2	NC	-0.581
C5	CA	0.698
N3	NH	-0.914
H6	H	0.452
H7	H	0.452
N4	NA	-0.302
H20	H	0.323
C6	CA	0.343
N5	NC	-0.570
C7	CA	0.337
H8	H4	0.109
C8	CM	-0.122
C9	CT	-0.119
H9	HC	0.117
H10	HC	0.117
C10	CA	-0.045
C11	CA	-0.256
H11	HA	0.145
C16	CA	0.301
O2	OS	-0.332
C17	CT	0.007
H17	H1	0.071
H18	H1	0.071
H19	H1	0.071
C15	CA	-0.225
H16	HA	0.167
C14	CA	-0.266
H15	HA	0.178
C12	CA	0.238
O1	OS	-0.296
C13	CT	-0.104
H12	H1	0.097
H13	H1	0.097
H14	H1	0.097

1efy

C15	CT	-0.037
H11	H1	0.085
H12	H1	0.085
H13	H1	0.085
O2	OS	-0.320
C12	CA	0.248
C13	CA	-0.188
C14	CA	-0.144
C11	CA	-0.171
H5	HA	0.130
H6	HA	0.159
H7	HA	0.146
C10	CA	-0.087
H4	HA	0.094
C6	CA	-0.097
C2	CR	0.423
N2	NA	-0.390
H10	H	0.340
N1	NB	-0.547
C1	CB	0.256
C3	CN	0.120
C4	CA	-0.264
H3	HA	0.189
C8	CA	-0.181
H2	HA	0.162
C7	CA	-0.140
H1	HA	0.158
C5	CM	-0.133
C9	C	0.745
O1	O	-0.606
N3	N	-0.886
H8	H	0.383
H9	H	0.383

1ela

N2	N3	-0.248
H11	H	0.277
H12	H	0.277
H13	H	0.277
C8	CT	-0.009
H9	HP	0.076
H10	HP	0.076
C7	CT	-0.033
H7	HC	0.043
H8	HC	0.043
C6	CT	-0.016
H5	HC	0.037
H6	HC	0.037
C5	CT	-0.075
H3	HC	0.053
H4	HC	0.053
C3	CT	0.112
N1	N	-0.296
C1	C	0.469
O1	O	-0.493
C2	CT	0.354

F1	F	-0.130
F2	F	-0.130
F3	F	-0.130
H1	H	0.225
H2	H1	0.076
C4	C	0.287
O2	O	-0.531
N3	N	-0.019
C13	CT	0.016
C12	CT	-0.056
C11	CT	-0.081
H15	HC	0.072
H16	HC	0.072
H17	HC	0.057
H18	HC	0.057
H19	H1	0.049
H20	H1	0.049
C9	CT	-0.026
H14	H1	0.090
C10	C	0.454
O3	O	-0.544
N4	N	-0.408
H22	H	0.316
C14	CA	0.083
C20	CA	-0.086
C19	CA	-0.268
H25	HA	0.169
H26	HA	0.159
C16	CA	-0.086
H23	HA	0.159
C17	CA	-0.268
H24	HA	0.169
C18	CA	0.030
C15	CT	0.247
C22	CT	-0.244
H30	HC	0.068
H31	HC	0.068
H32	HC	0.068
H21	HC	-0.009
C21	CT	-0.244
H27	HC	0.068
H28	HC	0.068
H29	HC	0.068

1etr

C1	CT	-0.077
H1	HC	0.043
H2	HC	0.043
C2	CT	0.220
C3	CT	-0.273
H3	HC	0.061
H4	HC	0.061
H5	HC	0.061
H6	HC	-0.007
C4	CT	-0.111
H7	H1	0.072
H36	H1	0.072
N1	NH	-0.226
H8	H	0.252
C5	CA	0.027
C6	CA	-0.003
C7	CA	-0.143
H9	HA	0.150
C8	CA	-0.258
H10	HA	0.165
C9	CA	-0.092
H11	HA	0.164
C10	CA	-0.010
S1	SY	0.758
O1	O	-0.497
O2	O	-0.497
N2	NT	-0.384
H12	H	0.228
C11	CT	0.061
C12	CT	-0.033
C13	CT	-0.010
C14	CT	0.313
N3	NH	-0.704
C15	CM	0.883
N4	NH	-0.892
H13	H	0.415
H14	H	0.415
N5	NH	-0.892
H15	H	0.415
H16	H	0.415
H17	H	0.366
H18	H1	0.010
H19	H1	0.010
H20	HC	0.031
H21	HC	0.031
H22	HC	0.022
H23	HC	0.022
H24	H1	0.069
C16	C	0.437
O3	O	-0.550
N6	N	-0.148
C17	CT	-0.282
C18	C	0.701
O4	O	-0.647
O5	O	-0.647
H25	H1	0.155
C19	CT	-0.068
H26	HC	0.046
H27	HC	0.046
C20	CT	0.159
C21	CT	-0.225
H28	HC	0.047
H29	HC	0.047
H30	HC	0.047
H31	HC	0.040
C22	CT	-0.135
H32	HC	0.040
H33	HC	0.040
C23	CT	0.011
H34	H1	0.067
H35	H1	0.067

1ett

N2	NH	-0.787
H9	H	0.431
H10	H	0.431
C17	CA	0.668
N3	NH	-0.787
H11	H	0.431
H12	H	0.431
C16	CA	-0.090
C15	CA	-0.154
C13	CA	-0.086
H17	HA	0.156
H19	HA	0.153
C14	CA	-0.154
H18	HA	0.153
C12	CA	-0.086
H16	HA	0.156
C11	CA	0.011
C10	CT	0.020
H14	HC	0.043
H15	HC	0.043
C8	CT	0.011
C9	C	0.426
O3	O	-0.522
N4	N	-0.220
C18	CT	-0.016
C19	CT	0.042
C20	CT	-0.030
C21	CT	0.042
C22	CT	-0.016
H28	H1	0.054
H29	H1	0.054
H26	HC	0.015
H27	HC	0.015
H24	HC	0.017
H25	HC	0.017
H22	HC	0.015
H23	HC	0.015
H20	H1	0.054
H21	H1	0.054
H13	H1	0.076
N1	NT	-0.371
H8	H	0.282
S1	SY	0.927
O1	O	-0.536
O2	O	-0.536
C1	CA	0.024
C2	CA	-0.148
H1	HA	0.161
C3	CA	-0.195
H2	HA	0.158
C4	CA	0.144
C7	CT	-0.198
H5	HC	0.078
H6	HC	0.078
H7	HC	0.078
C5	CA	-0.195
H3	HA	0.158
C6	CA	-0.148
H4	HA	0.161

1eve

C9	CT	-0.046
H28	HC	0.041
H29	HC	0.041
C4	CA	-0.101
C3	CA	-0.149
H22	HA	0.155
C2	CA	0.156
O3	OS	-0.340
C24	CT	-0.001
H17	H1	0.076
H18	H1	0.076
H19	H1	0.076
C1	CA	0.208
O2	OS	-0.229
C23	CT	-0.087
H14	H1	0.093
H15	H1	0.093
H16	H1	0.093
C6	CA	-0.217
H21	HA	0.168
C5	CA	-0.110
C7	C	0.514
O1	O	-0.522
C8	CT	0.016
H20	HC	0.032
C10	CT	-0.090
H12	HC	0.057
H13	HC	0.057
C11	CT	0.022
C15	CT	0.001
C14	CT	-0.096
H10	HP	0.111
H11	HP	0.111
H8	HC	0.035
H9	HC	0.035
H7	HC	0.026
C12	CT	0.001
H5	HC	0.035
H6	HC	0.035
C13	CT	-0.096
H3	HP	0.111
H4	HP	0.111
N1	N3	-0.017
H30	H	0.259
C16	CT	-0.088
H1	HP	0.115
H2	HP	0.115
C17	CA	0.009
C18	CA	-0.128
H23	HA	0.133
C19	CA	-0.160
H24	HA	0.167
C20	CA	-0.071
H25	HA	0.155
C21	CA	-0.160
H26	HA	0.167
C22	CA	-0.128
H27	HA	0.133

1exa

F1	F	-0.206
C1	CA	0.146
C2	CA	-0.184
H25	HA	0.156
C3	CA	-0.011
C7	C	0.807
O3	O	-0.781
O4	O	-0.781
C4	CA	-0.203
H24	HA	0.153
C5	CA	-0.168
H23	HA	0.163
C6	CA	-0.021
N1	N	-0.170
H17	H	0.233
C23	C	0.435
O2	O	-0.555
C22	CT	0.112
O1	OH	-0.643
H18	HO	0.423
H19	H1	0.085
C9	CA	0.025
C10	CA	-0.088
C11	CA	-0.319
H20	HA	0.179
H21	HA	0.146
C8	CA	-0.189
H22	HA	0.139
C17	CA	-0.043
C12	CA	0.004
C13	CT	0.291
C20	CT	-0.184
H7	HC	0.030
H8	HC	0.030
H9	HC	0.030
C21	CT	-0.184
H10	HC	0.030
H11	HC	0.030
H12	HC	0.030
C14	CT	-0.030
H13	HC	0.013
H14	HC	0.013
C15	CT	-0.127
H15	HC	0.011
H16	HC	0.011
C16	CT	0.286
C19	CT	-0.177
H4	HC	0.038
H5	HC	0.038
H6	HC	0.038
C18	CT	-0.177
H1	HC	0.038
H2	HC	0.038
H3	HC	0.038

1ezq

C20	CA	-0.005
H9	HA	0.084
C21	CA	-0.182
C26	CM	0.685
N3	NH	-0.779
H14	H	0.427
H15	H	0.427
N4	NH	-0.779
H16	H	0.427
H17	H	0.427
C22	CA	-0.106
H10	HA	0.143
C23	CA	-0.141
H11	HA	0.174
C24	CA	-0.095
H12	HA	0.173
C25	CA	0.004
C18	CT	-0.019
H21	HC	0.031
H22	HC	0.031
C16	CT	-0.044
C17	C	0.823
O1	OS	-0.420
C27	CT	-0.025

H18	H1	0.096
H19	H1	0.096
H20	H1	0.096
O2	O	-0.587
H23	HC	0.013
C14	CT	0.033
C15	CT	-0.317
H25	HC	0.111
H26	HC	0.111
H27	HC	0.111
H24	H1	0.084
N1	N	-0.390
H13	H	0.252
C13	C	0.636
O3	O	-0.559
C8	CA	-0.075
C9	CA	-0.158
C10	CA	-0.108
H5	HA	0.134
H6	HA	0.154
C7	CA	-0.158
H7	HA	0.154
C12	CA	-0.108
H8	HA	0.134
C11	CA	0.007
C2	CA	0.023
C1	CA	-0.133
H4	HA	0.138
C6	CA	-0.062
C19	CT	0.012
N2	N3	-0.353
H30	H	0.324
H31	H	0.324
H32	H	0.324
H28	HP	0.130
H29	HP	0.130
C5	CA	-0.136
H1	HA	0.161
C4	CA	-0.189
H2	HA	0.186
C3	CA	-0.051
H3	HA	0.143

1f0r

S1	S	-0.051
C10	CB	-0.071
C11	CA	-0.054
H7	HA	0.196
C12	CA	-0.334
H8	HA	0.161
C13	CA	0.341
H9	H4	0.066
N2	NC	-0.592
C14	CB	0.430
C15	C*	-0.110
H10	HA	0.166
C16	CA	-0.132
S2	SY	1.059
O1	O	-0.526
O2	O	-0.526
N4	NT	-0.673
H1	H	0.373
C18	CT	0.126
C19	CT	-0.076
C20	CT	-0.171
H14	H1	0.094
H15	H1	0.094
H12	HC	0.091
H13	HC	0.091
H11	H1	0.114
C17	C	0.420
O3	O	-0.538
N3	N	-0.088
C21	CT	-0.040
H18	H1	0.077
H19	H1	0.077
C6	CA	0.083
C5	CA	-0.243
H4	HA	0.168
C1	CA	-0.184

H2	HA	0.151
C2	CA	-0.230
H3	HA	0.166
C3	CA	0.153
C4	CA	-0.036
C9	CA	0.644
N5	NH	-0.906
H16	H	0.375
H17	H	0.375
N1	NC	-0.565
C8	CA	0.090
H6	H4	0.129
C7	CA	-0.306
H5	HA	0.142

1f0t

S1	S	-0.053
C9	CB	-0.014
C10	CA	-0.081
H10	HA	0.206
C11	CA	-0.323
H11	HA	0.173
C12	CA	0.335
H12	H4	0.085
N3	NC	-0.592
C13	CB	0.395
C14	C*	-0.018
H13	HA	0.167
C15	CA	-0.242
S2	SY	1.009
O2	O	-0.497
O3	O	-0.497
N5	NT	-0.787
H19	H	0.457
C17	CT	0.211
C18	CT	-0.138
C19	CT	-0.022
H17	H1	0.078
H18	H1	0.078
H15	HC	0.085
H16	HC	0.085
H14	H1	0.128
C16	C	0.507
O4	O	-0.539
N4	N	-0.253
C8	CT	-0.136
H7	H1	0.145
H8	H1	0.145
C4	CA	0.019
C5	CA	-0.140
H3	HA	0.198
C3	CA	0.327
O1	OH	-0.566
H9	HO	0.460
C2	CA	-0.268
H2	HA	0.178
C1	CA	-0.100
H1	HA	0.159
C6	CA	-0.200
C7	CM	0.756
N2	NH	-0.821
H6	H	0.430
H20	H	0.430
N1	NH	-0.821
H4	H	0.430
H5	H	0.430

1f4e

C4	CA	-0.074
H3	HA	0.140
C3	CA	-0.269
H2	HA	0.151
C2	CA	0.163
C7	CT	-0.126
H5	HC	0.038
H6	HC	0.038
H7	HC	0.038
C1	CA	-0.269
H1	HA	0.151

C6	CA	-0.074
H4	HA	0.140
C5	CA	-0.025
S1	SY	0.791
O1	O	-0.563
O2	O	-0.563
N1	NT	0.021
C8	CT	-0.083
H8	H1	0.024
H9	H1	0.024
C9	CT	-0.002
H10	HC	0.026
H11	HC	0.026
C10	CT	-0.031
H12	HC	-0.005
H13	HC	-0.005
C11	CT	0.043
H14	H1	0.039
C12	C	0.763
O4	O	-0.763
O3	O	-0.763

1f4f

O8	O	-0.857
C16	C	0.823
O9	O	-0.857
C15	CT	-0.049
H16	HC	-0.015
H17	HC	-0.015
C14	CT	0.068
H14	HC	-0.002
H15	HC	-0.002
C13	CT	0.033
C17	C	0.774
O6	O	-0.793
O7	O	-0.793
H13	H1	0.047
N2	N	-0.485
H12	H	0.268
C12	C	0.572
O5	O	-0.623
C9	CA	0.008
C10	CA	-0.160
C11	CA	-0.094
H11	HA	0.149
H10	HA	0.146
C8	CA	-0.160
H9	HA	0.146
C7	CA	-0.094
H8	HA	0.149
C6	CA	0.057
S1	SY	0.568
O3	O	-0.505
O4	O	-0.505
N1	NT	-0.245
C2	CT	-0.114
H3	H1	0.103
H4	H1	0.103
C1	CT	-0.037
H1	HC	0.027
H2	HC	0.027
C4	CT	0.078
H6	HC	-0.025
H7	HC	-0.025
C3	CT	0.054
H5	H1	0.057
C5	C	0.768
O2	O	-0.785
O1	O	-0.785

1f4g

O7	O	-0.843
C16	C	0.821
O8	O	-0.843
C15	CT	-0.070
H17	HC	-0.015
H18	HC	-0.015
C14	CT	-0.030
H15	HC	0.030

H16	HC	0.030
C13	CT	0.028
C17	C	0.788
O5	O	-0.795
O6	O	-0.795
H14	H1	0.069
N3	N	-0.582
H13	H	0.308
C12	C	0.744
O4	O	-0.650
C9	CA	-0.086
C10	CA	-0.135
C11	CA	-0.119
H12	HA	0.139
H11	HA	0.152
C8	CA	-0.135
H10	HA	0.152
C7	CA	-0.119
H9	HA	0.139
C6	CA	0.010
S1	SY	0.832
O2	O	-0.523
O3	O	-0.523
N1	NT	-0.298
C2	CT	-0.003
H3	H1	0.063
H4	H1	0.063
C1	CT	-0.047
H1	HC	0.035
H2	HC	0.035
C4	CT	-0.003
H6	HC	0.020
H7	HC	0.020
C3	CT	0.063
H5	H1	0.030
C5	C	0.598
O1	O	-0.603
N2	N	-0.494
H8	H	0.290
C18	CT	0.096
H19	H1	0.041
H20	H1	0.041
C19	CT	-0.043
H21	HC	-0.018
H22	HC	-0.018
C20	C	0.821
O10	O	-0.828
O9	O	-0.828

1fcx

C16	CT	-0.197
H14	HC	0.035
H15	HC	0.035
H16	HC	0.035
C12	CT	0.310
C17	CT	-0.197
H17	HC	0.035
H18	HC	0.035
H19	HC	0.035
C11	CT	-0.054
H6	HC	0.014
H7	HC	0.014
C10	CT	-0.085
H4	HC	0.008
H5	HC	0.008
C9	CT	0.278
C14	CT	-0.192
H8	HC	0.038
H9	HC	0.038
H10	HC	0.038
C15	CT	-0.192
H11	HC	0.038
H12	HC	0.038
H13	HC	0.038
C8	CA	-0.036
C23	CA	-0.231
H23	HA	0.141
C13	CA	-0.011
C24	CA	-0.297
H24	HA	0.178

C25	CA	-0.078
H25	HA	0.132
C22	CA	0.002
C26	CT	0.172
O3	OH	-0.669
H27	HO	0.423
H26	H1	0.062
C19	CA	0.059
C18	CA	-0.136
H20	HA	0.142
C5	CA	0.027
C4	CA	-0.272
H2	HA	0.129
C3	CA	-0.093
H1	HA	0.125
C2	CA	0.002
C1	C	0.783
O1	O	-0.777
O2	O	-0.777
C7	CA	-0.229
H3	HA	0.135
C6	CA	0.124
C21	CA	-0.199
H22	HA	0.149
C20	CA	-0.264
H21	HA	0.134

1fcz

C1	CA	-0.156
H1	HA	0.118
C2	CA	-0.178
H2	HA	0.139
C3	CA	0.042
C4	C	0.792
O1	O	-0.777
O2	O	-0.777
C5	CA	-0.178
H3	HA	0.139
C6	CA	-0.156
H4	HA	0.118
C7	CA	0.018
C8	CM	-0.040
H24	HA	0.102
C9	CM	-0.362
H25	HA	0.171
C10	C	0.605
O3	O	-0.572
C11	CA	-0.030
C12	CA	-0.263
H5	HA	0.192
C13	CA	-0.006
C14	CT	0.297
C15	CT	-0.210
H6	HC	0.042
H7	HC	0.042
H8	HC	0.042
C16	CT	-0.210
H9	HC	0.042
H10	HC	0.042
H11	HC	0.042
C17	CT	-0.087
H12	HC	0.015
H13	HC	0.015
C18	CT	-0.066
H14	HC	0.016
H15	HC	0.016
C19	CT	0.262
C20	CT	-0.212
H16	HC	0.044
H17	HC	0.044
H18	HC	0.044
C21	CT	-0.212
H19	HC	0.044
H20	HC	0.044
H21	HC	0.044
C22	CA	0.001
C23	CA	-0.266
H22	HA	0.174
C24	CA	-0.104
H23	HA	0.118

1fjs

O1	OH	-0.574
H24	HO	0.454
C1	CA	0.339
C2	CA	-0.259
H1	HA	0.162
C3	CA	-0.151
H2	HA	0.148
C4	CA	-0.192
C5	CM	0.772
N1	NH	-0.833
H3	H	0.405
H4	H	0.405
N2	NH	-0.833
H5	H	0.405
H6	H	0.405
C6	CA	-0.091
H7	HA	0.197
C7	CA	0.118
O2	OS	-0.258
C8	CA	0.300
C9	CA	0.194
F1	F	-0.197
C10	CA	0.068
N4	NH	-0.192
C11	CT	-0.216
C12	C	0.866
O3	O	-0.721
O4	O	-0.721
H8	H1	0.104
H9	H1	0.104
C13	CT	-0.216
H10	H1	0.106
H11	H1	0.106
H12	H1	0.106
C14	CA	0.070
F2	F	-0.151
N3	NC	-0.437
C15	CA	0.373
O5	OS	-0.323
C16	CA	0.187
C18	CA	-0.151
H14	HA	0.148
C19	CA	-0.155
H15	HA	0.146
C20	CA	-0.110
H16	HA	0.134
C17	CA	0.005
H13	HA	0.072
C21	CA	-0.253
C22	CM	0.722
N5	NH	-0.359
C23	CT	0.016
H17	H1	0.044
H18	H1	0.044
H19	H1	0.044
C24	CT	0.019
H20	H1	0.042
H21	H1	0.042
C25	CT	0.048
H22	H1	0.068
H23	H1	0.068
N6	N2	-0.659

1fkg

C21	CA	-0.183
C22	CA	-0.134
H1	HA	0.133
H2	HA	0.145
C20	CA	-0.100
H3	HA	0.129
C19	CA	-0.183
H4	HA	0.145
C18	CA	-0.134
H5	HA	0.133
C17	CA	0.010
C16	CT	-0.025
H11	HC	0.027
H12	HC	0.027
C15	CT	-0.005
H13	HC	0.034
H14	HC	0.034
C14	CT	0.058
C23	CA	0.034
C24	CA	-0.096
C25	CA	-0.175
C26	CA	-0.113
C27	CA	-0.175
C28	CA	-0.096
H6	HA	0.112
H7	HA	0.146
H8	HA	0.134
H9	HA	0.146
H10	HA	0.112
H15	H1	0.106
O1	OS	-0.383
C1	C	0.728
O2	O	-0.557
C2	CT	-0.019
H16	H1	0.074
C3	CT	-0.035
H17	HC	0.028
H18	HC	0.028
C4	CT	-0.032
H19	HC	0.018
H20	HC	0.018
C5	CT	0.014
H21	HC	0.019
H22	HC	0.019
C6	CT	-0.055
H23	H1	0.054
H24	H1	0.054
N1	N	-0.105
C7	C	0.316
O3	O	-0.499
C8	C	0.532
O4	O	-0.510
C9	CT	0.209
C12	CT	-0.159
H25	HC	0.033
H26	HC	0.033
H27	HC	0.033
C13	CT	-0.159
H28	HC	0.033
H29	HC	0.033
H30	HC	0.033
C10	CT	-0.051
H31	HC	0.023
H32	HC	0.023
C11	CT	-0.084
H33	HC	0.017
H34	HC	0.017
H35	HC	0.017

1fm6

O1	O	-0.522
C1	C	0.590
N1	N	-0.477
H1	H	0.359
C2	C	0.538
O2	O	-0.448
S1	S	-0.190
C3	CT	-0.015
H2	H1	0.097
C4	CT	-0.070
H3	HC	0.081
H4	HC	0.081
C5	CA	-0.027
C8	CA	-0.111
H7	HA	0.142
C9	CA	-0.157
H8	HA	0.124
C6	CA	-0.111
H6	HA	0.142
C7	CA	-0.157
H5	HA	0.124
C10	CA	0.108
O3	OS	-0.225
C11	CT	0.252
H9	H1	0.007
H10	H1	0.007
C12	CT	-0.229
H11	H1	0.143
H12	H1	0.143
N2	NH	-0.171
C13	CT	-0.128
H13	H1	0.113
H14	H1	0.113
H15	H1	0.113
C14	CA	0.272
N3	NA	-0.095
H20	H	0.318
C15	CA	-0.157
H16	H4	0.225
C16	CA	-0.114
H17	HA	0.180
C17	CA	-0.045
H18	HA	0.196
C18	CA	-0.170
H19	HA	0.153

1fm9

C30	CA	-0.145
C31	CA	-0.083
H25	HA	0.110
H24	HA	0.138
C29	CA	-0.161
H23	HA	0.142
C28	CA	-0.145
H22	HA	0.138
C27	CA	-0.083
H21	HA	0.110
C26	CA	-0.060
C22	CC	0.522
N2	NB	-0.570
O3	OS	-0.276
C24	CA	0.110
C25	CT	-0.212
H18	HC	0.082
H19	HC	0.082
H20	HC	0.082
C23	CC	0.150
C33	CT	0.028
H28	HC	0.005
H29	HC	0.005
C32	CT	0.070
H26	H1	0.058
H27	H1	0.058
O1	OS	-0.219
C8	CA	0.071
C7	CA	-0.149
C5	CA	-0.130
H6	HA	0.115
H8	HA	0.106
C6	CA	-0.149
H7	HA	0.106
C4	CA	-0.130
H5	HA	0.115
C3	CA	-0.045
C2	CT	-0.023
H3	HC	0.029
H4	HC	0.029
C1	CT	0.285
C34	C	0.601
O4	O	-0.715
O5	O	-0.715
H2	H1	-0.008
N1	NH	-0.616
H1	H	0.366
C9	CA	0.105
C14	CA	-0.062
C13	CA	-0.183
C12	CA	-0.197
C11	CA	-0.272
H9	HA	0.192
H10	HA	0.128
H11	HA	0.139
H12	HA	0.113

C10	CA	0.120
C21	C	0.382
O2	O	-0.550
C15	CA	0.014
C16	CA	-0.122
H13	HA	0.147
C17	CA	-0.177
H14	HA	0.140
C18	CA	-0.108
H15	HA	0.119
C19	CA	-0.177
H16	HA	0.140
C20	CA	-0.122
H17	HA	0.147

1frb

O2	O	-0.787
C18	C	0.807
O3	O	-0.787
C17	CT	-0.089
H10	HC	0.007
H11	HC	0.007
C4	CA	0.354
N2	NC	-0.598
C3	CA	0.027
C8	CA	-0.130
C7	CA	-0.131
C6	CA	-0.177
C5	CA	-0.132
H1	HA	0.141
H2	HA	0.136
H3	HA	0.140
H4	HA	0.151
C2	CA	0.016
C1	C	0.336
O1	O	-0.566
N1	N	0.295
C9	CT	-0.169
H5	H1	0.130
H6	H1	0.130
C10	CA	0.371
S1	S	-0.124
N3	NB	-0.555
C12	CB	0.297
C16	CA	-0.136
H9	HA	0.146
C11	CB	0.046
C13	CA	-0.194
H7	HA	0.211
C14	CA	-0.264
H8	HA	0.178
C15	CA	-0.024
C19	CT	0.488
F2	F	-0.184
F3	F	-0.184
F1	F	-0.184

1g4o

C1	CA	-0.153
H1	HA	0.117
C2	CA	-0.090
H2	HA	0.128
C3	CA	-0.028
S1	SY	1.140
N1	NT	-0.995
H3	H	0.328
O1	O	-0.662
O2	O	-0.662
C4	CA	-0.090
H4	HA	0.128
C5	CA	-0.153
H5	HA	0.117
C6	CA	-0.137
C7	C	0.670
O3	O	-0.558
N2	N	-0.521
H6	H	0.299
C8	CT	0.044
H7	H1	0.052
H8	H1	0.052
C9	CA	0.019
C10	CA	-0.101
H9	HA	0.132
C11	CA	-0.217
H10	HA	0.144
C12	CA	-0.080
H11	HA	0.119
C13	CA	-0.217
H12	HA	0.144
C14	CA	-0.101
H13	HA	0.132

1gwx

O1	O	-0.777
C1	C	0.740
O2	O	-0.777
C2	CT	0.570
C3	CT	-0.185
H12	HC	0.012
H13	HC	0.012
H14	HC	0.012
C4	CT	-0.185
H15	HC	0.012
H16	HC	0.012
H17	HC	0.012
O3	OS	-0.484
C5	CA	0.237
C10	CA	-0.125
C9	CA	-0.220
H10	HA	0.131
H9	HA	0.113
C6	CA	-0.125
H8	HA	0.113
C7	CA	-0.220
H7	HA	0.131
C8	CA	0.021
C11	CT	-0.024
H18	HC	0.020
H19	HC	0.020
C12	CT	-0.014
H20	HC	0.026
H21	HC	0.026
C13	CT	-0.103
H22	H1	0.067
H23	H1	0.067
N1	N	-0.130
C15	C	0.458
O4	O	-0.499
N2	N	-0.248
C16	CA	0.128
C17	CA	-0.136
C18	CA	-0.215
C19	CA	-0.059
C20	CA	-0.014
Cl1	Cl	-0.101
C21	CA	0.031
Cl2	Cl	-0.086
H1	HA	0.121
H2	HA	0.171
H3	HA	0.161
H11	H	0.175
C14	CT	-0.054
H24	H1	0.083
H25	H1	0.083
C22	CT	0.028
H26	HC	0.031
H27	HC	0.031
C23	CA	0.023
C24	CA	-0.057
Cl3	Cl	-0.104
C25	CA	-0.077
H6	HA	0.130
C26	CA	-0.212
H5	HA	0.174
C27	CA	-0.151
H4	HA	0.169
C28	CA	0.171
F1	F	-0.146

1h1p

N1	NA	-0.382
H1	H	0.370
C1	CR	0.238
H2	H5	0.184
N2	NB	-0.536
C2	CB	0.208
C3	CB	0.397
N3	NC	-0.523
C4	CA	0.543
N4	NH	-0.850
H3	H	0.441
H4	H	0.441
N5	NA	-0.208
H18	H	0.284
C5	CM	0.254
O1	OS	-0.186
C6	CT	-0.030
H5	H1	0.096
H6	H1	0.096
C7	CT	0.004
H7	HC	0.062
C8	CT	-0.032
H8	HC	0.019
H9	HC	0.019
C9	CT	-0.012
H10	HC	0.018
H11	HC	0.018
C10	CT	0.006
H12	HC	0.015
H13	HC	0.015
C11	CT	-0.012
H14	HC	0.018
H15	HC	0.018
C12	CT	-0.032
H16	HC	0.019
H17	HC	0.019

1h1s

C1	CM	-0.124
H1	HA	0.144
C2	CA	-0.127
H2	HA	0.189
C3	CA	-0.020
S1	SY	1.146
N1	NT	-1.045
H3	H	0.474
H4	H	0.474
O1	O	-0.544
O2	O	-0.544
C4	CA	-0.127
H5	HA	0.189
C5	CM	-0.124
H6	HA	0.144
C6	CM	0.076
N2	NH	-0.234
H7	H	0.320
C7	CA	0.261
N6	NA	-0.030
H23	H	0.182
N3	NC	-0.571
C8	CB	0.492
N5	NA	-0.459
C10	CR	0.263
N4	NB	-0.558
H9	H5	0.179
H8	H	0.392
C9	CB	0.240
C11	CM	0.185
O3	OS	-0.172
C12	CT	-0.024
H10	H1	0.096
H11	H1	0.096
C13	CT	0.007
H12	HC	0.064
C14	CT	-0.038
H13	HC	0.021
H14	HC	0.021
C15	CT	-0.020
H15	HC	0.020
H16	HC	0.020
C16	CT	0.007
H17	HC	0.016
H18	HC	0.016
C17	CT	-0.020
H19	HC	0.020
H20	HC	0.020
C18	CT	-0.038
H21	HC	0.021
H22	HC	0.021

1h9u

O1	O	-0.759
C17	C	0.720
O2	O	-0.759
C14	CA	-0.029
C15	CA	-0.093
C16	CA	-0.364
H9	HA	0.175
H8	HA	0.133
C13	CA	0.145
H7	H4	0.076
N1	NC	-0.506
C12	CA	0.402
C11	CX	0.028
C18	CX	-0.238
C19	CX	-0.238
H12	HC	0.093
H13	HC	0.093
H10	HC	0.093
H11	HC	0.093
C3	CA	0.000
C4	CA	0.048
C5	CA	-0.305
H2	HA	0.194
C24	CT	-0.091
H26	HC	0.038
H27	HC	0.038
H28	HC	0.038
C2	CA	-0.229
H1	HA	0.175
C1	CA	-0.062
C6	CA	-0.058
C7	CT	0.298
C20	CT	-0.151
H14	HC	0.018
H15	HC	0.018
H16	HC	0.018
C21	CT	-0.151
H17	HC	0.018
H18	HC	0.018
H19	HC	0.018
C8	CT	-0.013
H3	HC	-0.005
H4	HC	-0.005
C9	CT	-0.036
H5	HC	-0.020
H6	HC	-0.020
C10	CT	0.331
C23	CT	-0.138
H23	HC	0.015
H24	HC	0.015
H25	HC	0.015
C22	CT	-0.138
H20	HC	0.015
H21	HC	0.015
H22	HC	0.015

1hdq

O1	OH	-0.723
N1	N	-0.443
H1	H	0.299
C1	C	0.549
O2	O	-0.762
N2	N	-0.212
H2	H	0.244
C2	CT	0.052
C3	C	0.764

O3	O	-0.797
O4	O	-0.797
H3	H1	0.009
C4	CT	-0.048
H4	HC	0.017
H5	HC	0.017
C5	CA	-0.020
C6	CA	-0.097
H6	HA	0.147
C7	CA	-0.216
H7	HA	0.114
C8	CA	-0.139
H8	HA	0.094
C9	CA	-0.216
H9	HA	0.114
C10	CA	-0.097
H10	HA	0.147

1hfc

O1	OH	-0.647
N1	N	-0.253
H1	H	0.286
C1	C	0.527
O2	O	-0.668
C2	CT	-0.111
H2	HC	0.008
H3	HC	0.008
C3	CT	0.097
C4	CT	-0.171
C5	CT	0.102
C6	CT	-0.159
H4	HC	0.029
H5	HC	0.029
H6	HC	0.029
C7	CT	-0.159
H7	HC	0.029
H8	HC	0.029
H9	HC	0.029
H10	HC	0.056
H11	HC	0.056
H12	HC	0.056
H13	HC	0.050
C8	C	0.308
O3	O	-0.532
N2	N	-0.247
H14	H	0.213
C9	CT	0.108
C10	C	0.331
N3	N	-0.338
C11	CT	-0.146
H15	H1	0.081
H16	H1	0.081
H17	H1	0.081
H18	H	0.266
O4	O	-0.514
H19	H1	0.054
C12	CT	0.080
H20	HC	0.030
H21	HC	0.030
C13	CA	-0.090
C14	CA	-0.056
H22	HA	0.125
C15	CA	-0.210
H23	HA	0.128
C16	CA	-0.138
H24	HA	0.117
C17	CA	-0.210
H25	HA	0.128
C18	CA	-0.056
H26	HA	0.125

1hpv

C2	CA	-0.173
H1	HA	0.171
C1	CA	-0.218
H2	HA	0.162
C3	CA	0.340
N1	NH	-0.911
H34	H	0.395
H35	H	0.395
C4	CA	-0.218
H3	HA	0.162
C5	CA	-0.173
H4	HA	0.171
C6	CA	0.036
S1	SY	0.775
O1	O	-0.492
O2	O	-0.492
N2	NT	-0.225
C7	CT	-0.093
C8	CT	0.186
C9	CT	-0.197
H5	HC	0.044
H6	HC	0.044
H7	HC	0.044
C10	CT	-0.197
H8	HC	0.044
H9	HC	0.044
H10	HC	0.044
H11	HC	0.021
H12	H1	0.093
H13	H1	0.093
C11	CT	-0.034
H14	H1	0.103
H15	H1	0.103
C12	CT	0.050
O3	OH	-0.643
H16	HO	0.410
H17	H1	0.117
C13	CT	0.116
C14	CT	-0.005
C15	CA	-0.048
C16	CA	-0.103
C17	CA	-0.164
C18	CA	-0.127
C19	CA	-0.164
C20	CA	-0.103
H18	HA	0.123
H19	HA	0.139
H20	HA	0.131
H21	HA	0.139
H22	HA	0.123
H23	HC	0.047
H24	HC	0.047
H25	H1	0.112
N3	N	-0.501
H26	H	0.229
C21	C	0.807
O4	O	-0.633
O5	OS	-0.372
C22	CT	0.138
H27	H1	0.075
C23	CT	0.125
H28	H1	0.049
H29	H1	0.049
O6	OS	-0.453
C24	CT	0.204
H30	H1	0.022
H31	H1	0.022
C25	CT	-0.121
H32	HC	0.061
H33	HC	0.061

1htf

C23	CA	-0.100
H1	HA	0.124
C22	CA	-0.191
H2	HA	0.145
C21	CA	-0.105
H3	HA	0.131
C26	CA	-0.191
H4	HA	0.145
C25	CA	-0.100
H5	HA	0.124
C24	CA	0.003
C20	CT	-0.042
H19	HC	0.056
H20	HC	0.056
C9	CT	-0.013
C11	CT	-0.076
O4	OH	-0.629
H24	HO	0.435
H22	H1	0.095
H23	H1	0.095
H21	H1	0.109
N2	N	-0.061
H16	H	0.183
C8	C	0.251
O1	O	-0.496
C2	CT	0.012
N1	NT	-0.587
H32	H	0.373
H31	H1	0.176
C1	CT	0.239
C6	CT	-0.179
H28	HC	0.052
H29	HC	0.052
H30	HC	0.052
C7	CT	-0.179
H25	HC	0.052
H26	HC	0.052
H27	HC	0.052
S1	S	-0.274
C3	CT	0.031
H33	H2	0.167
C4	CT	-0.006
N3	N	-0.325
C12	C	0.530
C13	CT	-0.230
C15	CA	0.023
C14	CA	-0.122
C19	CA	-0.172
C18	CA	-0.107
C17	CA	-0.172
C16	CA	-0.122
H10	HA	0.133
H9	HA	0.145
H8	HA	0.129
H7	HA	0.145
H6	HA	0.133
H35	HC	0.108
H36	HC	0.108
O3	O	-0.550
H17	H	0.247
H34	H1	0.085
C5	C	0.370
O2	O	-0.464
N4	N	-0.338
H18	H	0.294
C10	CT	-0.049
H37	H1	0.088
H38	H1	0.088
C31	CA	0.030
C30	CA	-0.113
H15	HA	0.130
C29	CA	-0.189
H14	HA	0.144
C28	CA	-0.105
H13	HA	0.128
C27	CA	-0.189
H12	HA	0.144
C32	CA	-0.113
H11	HA	0.130

1i7z

C1	CT	-0.033
H1	H1	0.105
H2	H1	0.105
H3	H1	0.105
O1	OS	-0.369
C2	C	0.726
O2	O	-0.575
C3	CT	-0.040
H4	HC	0.068
C4	CT	-0.017
H9	HP	0.103
N1	N3	-0.014
C7	CT	-0.333
H10	HP	0.165
H11	HP	0.165
H12	HP	0.165
H22	H	0.291
C5	CT	-0.067
H7	HC	0.069
H8	HC	0.069
C6	CT	-0.053
H5	HC	0.067
H6	HC	0.067
C8	CT	-0.008
H13	HP	0.105
C9	CT	-0.071
H14	HC	0.067
H15	HC	0.067
C10	CT	0.175
H16	H1	0.127
O3	OS	-0.461
C11	C	0.687
O4	O	-0.540
C12	CA	-0.048
C13	CA	-0.121
H17	HA	0.151
C14	CA	-0.150
H18	HA	0.152
C15	CA	-0.083
H19	HA	0.151
C16	CA	-0.150
H20	HA	0.152
C17	CA	-0.121
H21	HA	0.151

1i8z

C1	CT	-0.105
H1	HP	0.122
H2	HP	0.122
C2	CT	0.167
H3	H1	0.064
H4	H1	0.064
O1	OS	-0.399
C3	CT	0.167
H5	H1	0.064
H6	H1	0.064
C4	CT	-0.105
H7	HP	0.122
H8	HP	0.122
N1	N3	-0.042
H21	H	0.213
C5	CT	-0.073
H9	HP	0.122
H10	HP	0.122
C6	CA	-0.158
C7	CA	-0.021
H11	HA	0.160
C8	CB	0.002
C9	C*	-0.148
H12	HA	0.158
C10	CA	-0.114
S1	SY	1.134
O2	O	-0.621
O3	O	-0.621
N2	NT	-0.920
H13	H	0.341
S2	S	0.146
C11	CB	-0.106
S3	SY	0.865
O4	O	-0.480
O5	O	-0.480
N3	NH	-0.078
C12	CA	-0.029
C13	CA	-0.063
H14	HA	0.100
C14	CA	0.183
O6	OS	-0.308
C15	CT	-0.015
H15	H1	0.080
H16	H1	0.080
H17	H1	0.080
C16	CA	-0.144
H18	HA	0.148
C17	CA	-0.202

H19	HA	0.179
C18	CA	-0.100
H20	HA	0.141

1if7

O1	O	-0.667
S1	SY	1.114
O2	O	-0.667
N1	NT	-0.939
H1	H	0.305
C1	CA	-0.020
C4	CA	-0.077
H4	HA	0.118
C5	CA	-0.156
H5	HA	0.125
C2	CA	-0.077
H3	HA	0.118
C3	CA	-0.156
H2	HA	0.125
C6	CA	-0.163
C7	C	0.735
O3	O	-0.579
N2	N	-0.638
H6	H	0.312
C8	CT	0.029
H7	H1	0.048
H8	H1	0.048
C9	CT	0.271
C10	CT	-0.070
H9	HC	0.002
H10	HC	0.002
H11	HC	0.002
H12	HC	0.004
C11	CT	-0.156
H13	H1	0.057
H14	H1	0.057
N3	NA	0.051
C12	CA	-0.191
H15	H4	0.187
C13	C*	-0.337
H16	HA	0.179
C14	CB	0.083
C15	CB	0.065
C16	CA	-0.192
H17	HA	0.146
C17	CA	-0.135
H18	HA	0.143
C18	CA	-0.230
H19	HA	0.146
C19	CA	-0.169
H20	HA	0.145

1iy7

N1	NT	-1.017
H1	H	0.328
S1	S6	1.230
O1	O	-0.710
O2	O	-0.710
N2	NT	-0.686
H2	H	0.344
C1	CT	0.196
C2	C	0.721
O3	O	-0.778
O4	O	-0.778
H3	H1	0.039
C3	CT	-0.059
H4	HC	0.027
H5	HC	0.027
C4	CA	0.006
C5	CA	-0.097
H6	HA	0.133
C6	CA	-0.199
H7	HA	0.109
C7	CA	-0.173
H8	HA	0.101
C8	CA	-0.199
H9	HA	0.109
C9	CA	-0.097
H10	HA	0.133

1jsv

C2	CM	-0.466
H2	HA	0.154
C1	CM	0.562
N1	NH	-0.915
H1	H	0.396
N2	NA	-0.325
H11	H	0.301
C3	CA	0.354
H3	H4	0.114
N3	NC	-0.657
C4	CA	0.521
N4	NH	-0.374
H4	H	0.309
C5	CA	0.065
C10	CA	-0.098
C9	CA	-0.176
H9	HA	0.187
H10	HA	0.130
C6	CA	-0.098
H5	HA	0.130
C7	CA	-0.176
H6	HA	0.187
C8	CA	-0.021
S1	SY	1.147
O2	O	-0.576
N5	NT	-0.975
H7	H	0.438
H8	H	0.438
O1	O	-0.576

1k1j

C17	CT	0.029
H20	H1	0.078
H21	H1	0.078
H22	H1	0.078
O3	OS	-0.400
C16	C	0.709
O2	O	-0.564
C15	CT	0.006
C13	CT	-0.015
C14	CT	0.111
H17	H1	0.030
H18	H1	0.030
H15	HC	0.010
H16	HC	0.010
H19	HC	0.010
C12	CT	-0.015
H13	HC	0.010
H14	HC	0.010
C11	CT	0.111
H11	H1	0.030
H12	H1	0.030
N3	N	-0.317
C10	C	0.507
O1	O	-0.548
C9	CT	0.053
C8	CT	-0.116
C1	CA	0.027
C2	CA	-0.081
C6	CA	-0.150
C5	CA	-0.092
C4	CA	-0.173
C3	CA	-0.067
H2	HA	0.138
H3	HA	0.177
H4	HA	0.132
C7	CA	0.669
N1	NH	-0.733
H5	H	0.405
H6	H	0.405
N2	NH	-0.733
H7	H	0.405
H31	H	0.405
H1	HA	0.122
H8	HC	0.085
H9	HC	0.085
H10	H1	0.186
N4	NT	-0.593
H23	H	0.382
S1	SY	0.855
O4	O	-0.518
O5	O	-0.518
C18	CA	-0.006
C23	CA	-0.064
H26	HA	0.149
C19	CA	-0.199
H24	HA	0.180
C20	CA	-0.205
H25	HA	0.177
C22	CA	0.139
C21	CA	0.057
C27	CA	-0.172
H30	HA	0.149
C26	CA	-0.119
H29	HA	0.146
C25	CA	-0.113
H28	HA	0.151
C24	CA	-0.196
H27	HA	0.147

1k22

N1	NH	-0.687
H1	H	0.380
H2	H	0.380
C1	CM	0.621
N2	NH	-0.687
H3	H	0.380
H4	H	0.380
C2	CA	-0.109
C5	CA	-0.129
H7	HA	0.152
C6	CA	-0.125
H8	HA	0.152
C3	CA	-0.129
H5	HA	0.152
C4	CA	-0.125
H6	HA	0.152
C7	CA	0.075
C8	CT	-0.038
H9	H1	0.108
H10	H1	0.108
N3	N	-0.527
H11	H	0.344
C9	C	0.647
O1	O	-0.513
C10	CT	-0.221
H12	H1	0.142
C11	CT	-0.091
H13	HC	0.097
H14	HC	0.097
C12	CT	-0.010
H15	H1	0.074
H16	H1	0.074
N4	N	-0.145
C13	C	0.479
O2	O	-0.543
C14	CT	-0.015
N5	N3	0.003
C15	CT	-0.062
C16	C	0.744
O3	O	-0.683
O4	O	-0.683
H17	HP	0.077
H18	HP	0.077
H19	H	0.209
H32	H	0.209
H20	HP	0.056
C17	CT	0.024
H21	HC	0.045
C18	CT	-0.040
H22	HC	0.020
H23	HC	0.020
C19	CT	-0.014
H24	HC	0.019
H25	HC	0.019
C20	CT	0.005
H26	HC	0.017
H27	HC	0.017
C21	CT	-0.014
H28	HC	0.019
H29	HC	0.019
C22	CT	-0.040
H30	HC	0.020
H31	HC	0.020

1k7e

O1	O	-0.799
C1	C	0.829
O2	O	-0.799
C2	CT	-0.039
H1	H1	0.054
H2	H1	0.054
N1	N	-0.496
H11	H	0.264
C3	C	0.554
O3	O	-0.605
C4	CT	-0.015
H3	HC	0.017
H4	HC	0.017
C5	C*	-0.064
C6	CW	-0.107
H5	H4	0.238
N2	NA	-0.466
H6	H	0.378
C7	CN	0.174
C8	CB	0.046
C9	CA	-0.181
H7	HA	0.141
C10	CA	-0.200
H8	HA	0.127
C11	CA	-0.165
H9	HA	0.130
C12	CA	-0.229
H10	HA	0.140

1k7f

O1	O	-0.766
C1	C	0.747
O2	O	-0.766
C2	CT	-0.016
C3	CT	0.128
C4	CT	-0.100
H1	HC	0.003
H2	HC	0.003
H3	HC	0.003
C5	CT	-0.100
H4	HC	0.003
H5	HC	0.003
H6	HC	0.003
H7	HC	0.009
H8	H1	0.069
N1	N	-0.226
H17	H	0.194
C6	C	0.366
O3	O	-0.563
C7	CT	0.011
H9	HC	0.025
H10	HC	0.025
C8	C*	-0.084
C9	CW	-0.096
H11	H4	0.219
N2	NA	-0.429
H12	H	0.366
C10	CN	0.143
C11	CA	-0.198
H13	HA	0.133
C12	CA	-0.195
H14	HA	0.136
C13	CA	-0.191
H15	HA	0.128
C14	CA	-0.176
H16	HA	0.133
C15	CB	0.054

1kv1

Cl1	Cl	-0.127
C5	CA	0.009
C6	CA	-0.091
C7	CM	-0.152
H3	HA	0.145
H4	HA	0.142
C4	CA	-0.091
H1	HA	0.142
C3	CM	-0.152
H2	HA	0.145
C2	CA	0.115
N1	N	-0.523
H5	H	0.290
C1	C	0.808
O1	O	-0.491
N2	N	-0.602
H6	H	0.355
C8	CR	0.184
C9	C*	-0.392
H7	HA	0.177
C10	CV	0.149
C15	CT	0.531
C12	CT	-0.199
H14	HC	0.027
H15	HC	0.027
H16	HC	0.027
C13	CT	-0.199
H11	HC	0.027
H12	HC	0.027
H13	HC	0.027
C14	CT	-0.199
H8	HC	0.027
H9	HC	0.027
H10	HC	0.027
N3	NB	-0.543
N4	N*	0.217
C11	CT	-0.121
H17	H1	0.077
H18	H1	0.077
H19	H1	0.077

1kv2

C2	CT	-0.094
H1	HP	0.127
H2	HP	0.127
C1	CT	0.136
H3	H1	0.071
H4	H1	0.071
O1	OS	-0.368
C3	CT	0.136
H5	H1	0.071
H6	H1	0.071
C4	CT	-0.094
H7	HP	0.127
H8	HP	0.127
N1	N3	-0.017
H38	H	0.291
C5	CT	-0.256
H9	HP	0.177
H10	HP	0.177
C6	CT	-0.034
H11	H1	0.140
H12	H1	0.140
O2	OS	-0.312
C7	CA	0.020
C10	CA	0.085
C11	CA	-0.220
C12	CA	-0.116
C13	CA	-0.150
C14	CA	-0.133
H15	HA	0.103
C15	CA	0.097
H16	HA	0.163
H17	HA	0.152
H18	HA	0.149
C8	CA	-0.131
H14	HA	0.153
C9	CM	-0.212
H13	HA	0.195
C16	CA	0.085

N2	N	-0.511
H19	H	0.348
C17	C	0.727
O3	O	-0.525
N3	N	-0.424
H20	H	0.230
C18	CR	0.153
C19	C*	-0.413
H21	HA	0.197
C20	CV	0.170
C21	CT	0.503
C22	CT	-0.274
H22	HC	0.053
H23	HC	0.053
H24	HC	0.053
C23	CT	-0.274
H25	HC	0.053
H26	HC	0.053
H27	HC	0.053
C24	CT	-0.274
H28	HC	0.053
H29	HC	0.053
H30	HC	0.053
N4	NB	-0.480
N5	N*	0.206
C25	CA	0.049
C26	CM	-0.131
H31	HA	0.141
C27	CA	-0.193
H32	HA	0.154
C28	CA	0.085
C29	CT	-0.157
H33	HC	0.064
H34	HC	0.064
H35	HC	0.064
C30	CA	-0.193
H36	HA	0.154
C31	CM	-0.131
H37	HA	0.141

1l2s

Cl1	Cl	-0.165
C6	CA	0.010
C5	CA	-0.149
C3	CA	-0.128
H3	HA	0.152
H5	HA	0.148
C4	CA	-0.149
H4	HA	0.148
C2	CA	-0.128
H2	HA	0.152
C1	CA	0.312
N1	NH	-0.746
H1	H	0.386
S1	SY	0.996
O1	O	-0.545
O2	O	-0.545
C7	CA	-0.019
C8	CA	-0.008
C10	C	0.740
O3	O	-0.685
O4	O	-0.685
S2	S	-0.014
C11	CA	-0.269
H7	H4	0.206
C9	CA	-0.174
H6	HA	0.159

1l8g

C2	CA	-0.162
H1	HA	0.166
C1	CA	-0.136
H2	HA	0.137
C4	CA	-0.051
H3	HA	0.131
C5	CA	-0.091
H4	HA	0.174
C6	CB	-0.054
C3	CB	-0.103

S1	SY	1.115
O1	O	-0.578
O2	O	-0.578
N1	NB	-0.609
C7	CV	0.606
O3	OS	-0.228
C8	CT	-0.026
H5	H1	0.096
H6	H1	0.096
C9	CT	0.202
H7	H1	0.057
C15	CA	-0.102
S2	S	-0.206
O4	OS	-0.423
C10	CT	0.055
H8	H1	0.026
H9	H1	0.026
C11	CT	0.081
H10	HC	0.028
H11	HC	0.028
C12	C*	0.027
C13	C*	-0.179
C14	C	0.790
O5	O	-0.771
O6	O	-0.771
C16	CC	0.064
N2	N	-0.203
H12	H	0.267
C17	C	0.463
O7	O	-0.553
C18	C	0.697
O9	O	-0.754
O8	O	-0.754

1lqd

N3	NH	-0.751
H26	H	0.412
H27	H	0.412
C25	CA	0.676
N4	NH	-0.751
H28	H	0.412
H29	H	0.412
C18	CA	-0.178
C19	CA	-0.068
H21	HA	0.142
C17	CA	-0.067
H20	HA	0.131
C16	CA	-0.163
H19	HA	0.173
C15	CA	-0.064
H18	HA	0.142
C14	CA	0.037
C13	CT	-0.018
H13	H1	0.074
H14	H1	0.074
N1	N*	-0.164
C3	CB	0.048
C2	CA	-0.204
H25	HA	0.162
C1	CA	-0.168
H24	HA	0.166
C6	CA	-0.197
H23	HA	0.159
C5	CA	0.047
C9	CT	-0.134
H10	HC	0.058
H11	HC	0.058
H12	HC	0.058
C4	CB	0.088
C8	C*	-0.221
H22	HA	0.189
C7	C*	-0.037
C10	C	0.423
O1	O	-0.559
N2	N	-0.184
H1	H	0.220
C11	CT	-0.060
H2	H1	0.094
H3	H1	0.094
C12	CA	-0.039

C20	CA	-0.166
H16	HA	0.132
C21	CA	0.033
C26	CT	-0.145
H4	HC	0.063
H5	HC	0.063
H6	HC	0.063
C22	CA	-0.174
H15	HA	0.156
C23	CA	0.033
C27	CT	-0.145
H7	HC	0.063
H8	HC	0.063
H9	HC	0.063
C24	CA	-0.166
H17	HA	0.132

1m48

C2	CT	-0.064
H4	H1	0.069
H5	H1	0.069
N3	NH	-0.204
C1	C2	0.462
N1	NH	-0.616
H1	H	0.350
H31	H	0.350
N2	NH	-0.616
H2	H	0.350
H3	H	0.350
C3	CT	0.003
H6	H1	0.077
H7	H1	0.077
C4	CT	0.003
H8	HC	0.029
H9	HC	0.029
C5	CT	0.003
H10	HC	0.019
H11	HC	0.019
C6	CT	0.078
H12	HC	0.057
C7	CT	-0.111
H13	HC	0.063
H14	HC	0.063
C8	C	0.458
O1	O	-0.522
N4	N	-0.390
H15	H	0.271
C9	CT	-0.037
C10	C	0.816
O2	O	-0.573
O3	OS	-0.417
C11	CT	-0.005
H17	H1	0.092
H18	H1	0.092
H19	H1	0.092
H16	H1	0.110
C12	CT	-0.128
H20	HC	0.088
H21	HC	0.088
C13	CA	0.012
C16	CA	-0.154
C17	CA	-0.088
H25	HA	0.133
H24	HA	0.139
C14	CA	-0.154
H22	HA	0.139
C15	CA	-0.088
H23	HA	0.133
C18	CA	0.052
C19	CZ	-0.094
C20	CZ	-0.132
C21	CA	0.066
C22	CA	-0.084
H26	HA	0.130
C23	CA	-0.188
H27	HA	0.159
C26	CA	-0.090
H30	HA	0.149
C25	CA	-0.188
H29	HA	0.159

C24	CA	-0.084
H28	HA	0.130

1mmb

S1	S	-0.029
C1	CA	-0.222
H1	H4	0.200
C2	C*	-0.254
H2	HA	0.161
C3	C*	-0.010
H3	HA	0.143
C4	CA	-0.001
S2	S	-0.165
C5	CT	-0.170
H4	H1	0.154
H5	H1	0.154
C6	CT	-0.265
C7	C	0.868
N1	N	-0.346
O1	OH	-0.690
H6	H	0.266
O2	O	-0.750
H7	HC	0.066
C8	CT	-0.023
C9	CT	-0.080
C10	CT	0.174
C11	CT	-0.363
H8	HC	0.087
H9	HC	0.087
H10	HC	0.087
C12	CT	-0.363
H11	HC	0.087
H12	HC	0.087
H13	HC	0.087
H14	HC	0.050
H15	HC	0.071
H16	HC	0.071
H17	HC	0.060
C13	C	0.103
O3	O	-0.446
N2	N	-0.252
H18	H	0.292
C14	CT	0.077
C15	C	0.499
N3	N	-0.413
C16	CT	-0.115
H19	H1	0.078
H20	H1	0.078
H21	H1	0.078
H22	H	0.298
O4	O	-0.558
H23	H1	0.057
C17	CT	-0.035
H24	HC	0.038
H25	HC	0.038
C18	CA	0.016
C19	CA	-0.168
H26	HA	0.141
C20	CA	-0.131
H27	HA	0.132
C21	CA	-0.141
H28	HA	0.131
C22	CA	-0.131
H29	HA	0.132
C23	CA	-0.168
H30	HA	0.141

1mnc

O1	OH	-0.604
N1	N	-0.242
H24	H	0.260
C1	C	0.488
O2	O	-0.648
C2	CT	0.011
H1	HC	-0.038
H2	HC	-0.038
C3	CT	0.189
C4	CT	-0.086
C5	CT	0.085
C6	CT	-0.211
H7	HC	0.043
H8	HC	0.043
H9	HC	0.043
C7	CT	-0.211
H10	HC	0.043
H11	HC	0.043
H12	HC	0.043
H6	HC	0.063
H4	HC	0.016
H5	HC	0.016
H3	HC	-0.025
C8	C	0.473
O3	O	-0.589
N2	N	-0.402
H25	H	0.265
C9	CT	0.148
C10	C	0.501
N3	N	-0.391
C11	CT	-0.197
H14	H1	0.096
H15	H1	0.096
H16	H1	0.096
H26	H	0.266
O4	O	-0.566
H13	H1	0.008
C12	CT	-0.091
H17	HC	0.059
H18	HC	0.059
C13	CA	0.090
C14	CA	-0.161
H19	HA	0.110
C15	CA	-0.161
H20	HA	0.109
C16	CA	-0.106
H21	HA	0.108
C17	CA	-0.161
H22	HA	0.109
C18	CA	-0.161
H23	HA	0.110

1mq5

C3	CA	-0.175
H1	HA	0.157
C2	CA	-0.065
H2	HA	0.145
C1	CA	-0.024
Cl1	Cl	-0.086
C4	CA	-0.065
H3	HA	0.145
C5	CA	-0.175
H4	HA	0.157
C6	CA	0.122
N1	N	-0.398
H5	H	0.297
C7	C	0.564
O1	O	-0.522
C8	CA	-0.059
C9	CA	-0.077
H8	HA	0.140
C10	CA	-0.055
Cl2	Cl	-0.056
C11	CA	-0.015
H7	HA	0.149
C12	CA	-0.147
H6	HA	0.155
C13	CA	-0.005
N2	N	-0.214
H9	H	0.221
C14	C	0.598
O2	O	-0.497
C15	C*	-0.154
S1	S	0.180
C17	CA	-0.243
H10	H4	0.233
C16	C*	-0.029
Cl3	Cl	-0.041
C18	C*	0.053
C19	CT	-0.154
H11	HP	0.167
H12	HP	0.167
N3	N3	-0.086
H24	H	0.294
C20	CT	0.018
H13	HP	0.106
H14	HP	0.106
C21	CT	-0.142
H15	HP	0.171
H16	HP	0.171
N4	N3	-0.044
C22	CT	-0.406
H17	HP	0.212
H18	HP	0.212
H19	HP	0.212
H25	H	0.351
C23	CT	-0.142
H20	HP	0.171
H21	HP	0.171
C24	CT	0.018
H22	HP	0.106
H23	HP	0.106

1mq6

C3	CA	-0.331
H1	HA	0.162
C2	CA	0.001
H2	HA	0.163
C1	CM	-0.122
Cl1	Cl	-0.091
C4	CA	0.256
H3	H4	0.101
N1	NC	-0.508
C5	CA	0.589
N2	N	-0.589
H4	H	0.318
C6	C	0.610
O1	O	-0.526
C7	CA	-0.018
C8	CA	-0.113
H9	HA	0.147
C9	CA	-0.110
Cl2	Cl	-0.056
C10	CA	-0.014
H8	HA	0.117
C11	CA	0.148
O2	OS	-0.254
C12	CT	-0.024
H5	H1	0.084
H6	H1	0.084
H7	H1	0.084
C13	CA	0.027
N3	N	-0.402
H10	H	0.305
C14	C	0.613
O3	O	-0.507
C15	C*	-0.050
S1	S	0.027
C17	CA	-0.148
H11	H4	0.237
C16	C*	-0.019
Cl3	Cl	-0.025
C18	C*	-0.050
C19	CT	-0.018
H12	HP	0.111
H13	HP	0.111
N4	N3	-0.146
C20	CT	-0.071
H14	HP	0.106
H15	HP	0.106
H16	HP	0.106
H21	H	0.310
C21	C2	0.736
N5	N2	-0.553
C22	CT	0.041
H17	H1	0.114
H18	H1	0.114
C23	CT	-0.007
H19	H1	0.127
H20	H1	0.127
O4	OS	-0.430

1nhu

F1	F	-0.212
C24	CT	0.578
F2	F	-0.212
F3	F	-0.212
C22	CA	-0.055
C23	CA	-0.158
H16	HA	0.152
C21	CA	-0.132
H13	HA	0.132
C20	CA	-0.195
H14	HA	0.149
C19	CA	-0.079
H15	HA	0.153
C18	CA	0.013
C17	CT	-0.164
H4	H1	0.135
H5	H1	0.135
N1	N	-0.078
C3	C	0.384
O3	O	-0.554
C4	CA	0.106
C5	CA	-0.090
C6	CA	-0.154
C7	CA	0.004
C8	CA	-0.031
C9	CA	-0.070
Cl2	Cl	-0.091
H6	HA	0.116
Cl1	Cl	-0.141
H7	HA	0.153
H8	HA	0.176
C1	CT	0.059
C2	C	0.714
O1	O	-0.753
O2	O	-0.753
H3	H1	0.037
C10	CT	-0.012
H1	HC	0.035
H2	HC	0.035
C11	CA	-0.021
C12	CA	-0.122
H12	HA	0.125
C13	CA	-0.178
H11	HA	0.130
C14	CA	-0.127
H10	HA	0.116
C15	CA	-0.178
H9	HA	0.130
C16	CA	-0.122
H17	HA	0.125

1nhv

C15	CA	-0.157
C16	CA	-0.125
H11	HA	0.118
H10	HA	0.128
C14	CA	-0.159
H9	HA	0.123
C13	CA	-0.157
H8	HA	0.128
C12	CA	-0.125
H7	HA	0.118
C11	CA	-0.015
C10	CT	-0.024
H5	HC	0.050
H6	HC	0.050
C1	CT	0.058
C2	C	0.694
O1	O	-0.748
O2	O	-0.748
H1	H1	0.060
N1	N	-0.172
C3	C	0.443
O3	O	-0.563
C4	CA	0.102
C5	CA	-0.122
C6	CA	-0.123

C7	CA	0.008
C8	CA	-0.033
C9	CA	-0.078
Cl2	Cl	-0.087
H4	HA	0.119
Cl1	Cl	-0.147
H3	HA	0.144
H2	HA	0.184
C17	CT	-0.167
H12	H1	0.175
H13	H1	0.175
C18	CA	0.025
S1	S	-0.130
C19	C*	-0.203
H14	HA	0.185
C20	C*	-0.056
H15	HA	0.124
C21	C*	-0.032
C22	C*	0.220
C23	C*	-0.366
H16	HA	0.217
C24	CB	0.091
C25	CA	-0.203
H17	HA	0.156
C26	CA	-0.210
H18	HA	0.146
C27	CA	-0.139
H19	HA	0.140
C28	CA	-0.267
H20	HA	0.170
C29	CB	0.287
O4	OS	-0.283

1o86

O1	O	-0.683
C1	C	0.680
O2	O	-0.683
C2	CT	0.024
H7	H1	0.056
C3	CT	-0.032
H5	HC	0.038
H6	HC	0.038
C4	CT	-0.051
H3	HC	0.053
H4	HC	0.053
C5	CT	-0.150
H1	H1	0.093
H2	H1	0.093
N1	N	-0.009
C6	C	0.205
O3	O	-0.493
C7	CT	0.003
C8	CT	-0.001
C9	CT	-0.051
C10	CT	-0.082
C11	CT	0.080
N2	N3	-0.337
H8	H	0.284
H9	H	0.284
H31	H	0.284
H10	HP	0.063
H11	HP	0.063
H12	HC	0.048
H13	HC	0.048
H14	HC	0.027
H15	HC	0.027
H16	HC	0.054
H17	HC	0.054
H18	HP	0.076
N3	N3	-0.006
H29	H	0.209
H30	H	0.209
C12	CT	0.006
C13	C	0.717
O4	O	-0.710
O5	O	-0.710
H19	HP	0.054
C14	CT	0.063
H20	HC	-0.005
H21	HC	-0.005
C15	CT	-0.028
H22	HC	0.049
H23	HC	0.049
C16	CA	0.013
C17	CA	-0.150
H24	HA	0.141
C18	CA	-0.175
H25	HA	0.141
C19	CA	-0.103
H26	HA	0.126
C20	CA	-0.175
H27	HA	0.141
C21	CA	-0.150
H28	HA	0.141

1ohr

C3	CT	-0.151
H2	HC	0.068
H3	HC	0.068
H4	HC	0.068
C2	CA	0.039
C1	CA	0.286
O1	OH	-0.588
H1	HO	0.446
C4	CA	-0.237
H5	HA	0.167
C5	CA	-0.207
H6	HA	0.189
C6	CA	-0.122
H7	HA	0.133
C7	CA	-0.069
C8	C	0.327
O2	O	-0.437
N1	N	-0.274
H8	H	0.239
C9	CT	0.143
C10	CT	-0.225
S1	S	-0.246
C11	CA	0.145
C12	CA	-0.089
C13	CA	-0.212
C14	CA	-0.082
C15	CA	-0.212
C16	CA	-0.089
H9	HA	0.126
H10	HA	0.167
H11	HA	0.151
H12	HA	0.167
H13	HA	0.126
H14	H1	0.114
H15	H1	0.114
H16	H1	0.131
C17	CT	0.102
O3	OH	-0.654
H17	HO	0.433
H18	H1	0.166
C18	CT	-0.004
H19	HP	0.075
H20	HP	0.075
N2	N3	-0.001
H46	H	0.168
C19	CT	-0.006
H21	HP	0.065
H22	HP	0.065
C28	CT	0.029
H37	HC	0.049
C29	CT	-0.032
H38	HC	0.020
H39	HC	0.020
C30	CT	0.016
H40	HC	0.014
H41	HC	0.014
C31	CT	-0.009
H42	HC	0.019
H43	HC	0.019
C32	CT	-0.042
H44	HC	0.023
H45	HC	0.023
C27	CT	-0.015
H36	HC	0.013
C26	CT	-0.067
H34	HC	0.084
H35	HC	0.084
C20	CT	0.014
H33	HP	0.079
C21	C	0.095
O4	O	-0.361
N3	N	-0.233
H32	H	0.268
C22	CT	0.192
C24	CT	-0.165
H26	HC	0.058
H27	HC	0.058
H28	HC	0.058
C25	CT	-0.165
H29	HC	0.058
H30	HC	0.058
H31	HC	0.058
C23	CT	-0.165
H23	HC	0.058
H24	HC	0.058
H25	HC	0.058

1ppc

N3	NH	-0.791
H15	H	0.430
H22	H	0.430
C22	CA	0.670
N4	NH	-0.791
H16	H	0.430
H17	H	0.430
C21	CA	-0.079
C20	CA	-0.179
C18	CA	-0.105
H19	HA	0.168
H21	HA	0.154
C19	CA	-0.179
H20	HA	0.154
C17	CA	-0.105
H18	HA	0.168
C16	CA	0.090
C15	CT	-0.092
H13	HC	0.062
H14	HC	0.062
C13	CT	0.121
C14	C	0.413
O4	O	-0.531
N5	N	-0.153
C23	CT	0.018
C24	CT	0.007
C25	CT	-0.067
C26	CT	0.007
C27	CT	0.018
H31	H1	0.034
H32	H1	0.034
H29	HC	0.026
H30	HC	0.026
H27	HC	0.022
H28	HC	0.022
H25	HC	0.026
H26	HC	0.026
H23	H1	0.034
H24	H1	0.034
H12	H1	0.036
N2	N	-0.322
H11	H	0.278
C12	C	0.559
O3	O	-0.591
C11	CT	-0.066
H9	H1	0.121
H10	H1	0.121
N1	NT	-0.655
H8	H	0.395
S1	SY	0.928
O1	O	-0.505
O2	O	-0.505
C2	CA	-0.025
C1	CA	-0.131
H1	HA	0.154
C9	CA	0.106
C8	CA	-0.221
H7	HA	0.165
C7	CA	-0.121
H6	HA	0.151
C6	CA	-0.103
H5	HA	0.149
C5	CA	-0.209
H4	HA	0.153
C10	CA	0.120
C4	CA	-0.197
H3	HA	0.166
C3	CA	-0.165
H2	HA	0.165

1pph

C5	CA	-0.162
C6	CA	-0.155
H4	HA	0.168
H3	HA	0.156
C4	CA	0.111
C7	CT	-0.191
H5	HC	0.080
H6	HC	0.080
H7	HC	0.080
C3	CA	-0.162
H2	HA	0.156
C2	CA	-0.155
H1	HA	0.168
C1	CA	0.013
S1	SY	0.815
O1	O	-0.506
O2	O	-0.506
N1	NT	-0.471
H15	H	0.330
C9	CT	0.070
C10	CT	0.033
C11	CA	-0.004
C12	CA	-0.091
C14	CA	-0.170
C16	CA	-0.077
C15	CA	-0.162
C13	CA	-0.049
H12	HA	0.141
H13	HA	0.170
H14	HA	0.128
C17	CA	0.693
N2	NH	-0.760
H16	H	0.412
H17	H	0.412
N3	NH	-0.760
H18	H	0.412
H19	H	0.412
H11	HA	0.157
H9	HC	0.022
H10	HC	0.022
H8	H1	0.129
C8	C	0.358
O3	O	-0.512
N4	N	-0.124
C18	CT	-0.022
H20	H1	0.055
H21	H1	0.055
C19	CT	0.003
H22	HC	0.023
H23	HC	0.023
C20	CT	-0.041
H24	HC	0.027
H25	HC	0.027
C21	CT	0.003
H26	HC	0.023
H27	HC	0.023
C22	CT	-0.022
H28	H1	0.055
H29	H1	0.055

1qbu

C16	CV	-0.013
N4	NB	-0.383
H15	H4	0.146

C17	CA	-0.317
H16	H4	0.227
S1	S	0.038
C15	CR	0.355
N3	N	-0.307
H17	H	0.175
C14	C	0.582
O4	O	-0.536
C10	CA	-0.078
C11	CA	-0.080
C12	CA	-0.197
C13	CA	-0.131
H11	HA	0.143
H12	HA	0.158
H13	HA	0.149
C9	CA	-0.142
H14	HA	0.141
C8	CA	0.006
C2	CT	-0.041
H18	H1	0.083
H19	H1	0.083
N1	N	0.002
C1	C	0.273
O1	O	-0.528
N2	N	0.005
C7	CT	-0.248
C32	CX	0.018
C33	CX	-0.347
C34	CX	-0.347
H29	HC	0.156
H30	HC	0.156
H31	HC	0.156
H32	HC	0.156
H28	HC	0.072
H26	H1	0.148
H27	H1	0.148
C6	CT	0.006
C25	CT	-0.088
C26	CA	0.012
C27	CA	-0.127
C28	CA	-0.184
C29	CA	-0.096
C30	CA	-0.184
C31	CA	-0.127
H5	HA	0.124
H4	HA	0.147
H3	HA	0.127
H2	HA	0.147
H1	HA	0.124
H33	HC	0.055
H34	HC	0.055
H25	H1	0.053
C5	CT	0.170
O3	OH	-0.650
H35	HO	0.421
H22	H1	0.043
C4	CT	0.087
O2	OH	-0.660
H36	HO	0.444
H21	H1	0.078
C3	CT	-0.007
H20	H1	0.112
C18	CT	-0.029
H23	HC	0.052
H24	HC	0.052
C19	CA	-0.021
C20	CA	-0.121
H10	HA	0.126
C21	CA	-0.126
H9	HA	0.126
C22	CA	-0.173
H8	HA	0.145
C23	CA	-0.126
H7	HA	0.126
C24	CA	-0.121
H6	HA	0.126

1qhi

O1	OH	-0.701
H10	HO	0.429
C1	CT	0.235
H8	H1	0.005
H9	H1	0.005
C2	CT	-0.012
H6	HC	0.044
H7	HC	0.044
C3	CT	-0.056
H4	HC	0.036
H5	HC	0.036
C4	CT	-0.094
H2	H1	0.077
H3	H1	0.077
N1	N*	-0.021
C9	CK	0.112
N5	NB	-0.559
C8	CB	0.199
C7	C	0.525
N4	N	-0.505
H12	H	0.339
O2	O	-0.547
H11	H5	0.161
C5	CB	0.101
N2	NC	-0.399
C6	CA	0.497
N3	NH	-0.471
H1	H	0.325
C13	CM	0.122
C12	CM	-0.117
H13	HA	0.137
C11	CA	-0.201
H17	HA	0.152
C10	CA	-0.067
H16	HA	0.122
C15	CA	-0.201
H15	HA	0.152
C14	CM	-0.117
H14	HA	0.137

1ql9

C2	CA	-0.204
H3	HA	0.173
C1	CA	-0.073
H2	H4	0.212
N1	NA	-0.100
H1	H	0.320
C4	CA	-0.073
H5	H4	0.212
C3	CA	-0.204
H4	HA	0.173
C5	CA	0.321
N2	NH	-0.195
C9	CT	0.004
C8	CT	-0.064
H21	HC	0.066
H22	HC	0.066
H23	H1	0.065
H24	H1	0.065
C6	CT	0.004
H25	H1	0.065
H26	H1	0.065
C7	CT	-0.064
H27	HC	0.066
H28	HC	0.066
C10	CT	0.068
H20	HC	0.023
C11	C	0.335
O1	O	-0.536
N3	N	-0.117
C15	CT	-0.029
C14	CT	-0.022
H18	H1	0.090
H19	H1	0.090
H16	H1	0.090
H17	H1	0.090
C12	CT	-0.029
H14	H1	0.090
H15	H1	0.090
C13	CT	-0.022
H12	H1	0.090
H13	H1	0.090
N4	NT	-0.374
S1	SY	0.844
O2	O	-0.491
O3	O	-0.491
C16	CA	-0.004
C18	CA	-0.217
C19	CA	-0.177
H7	HA	0.167
H6	HA	0.189
C17	CA	-0.039
H11	HA	0.138
C20	CA	0.055
C21	CA	0.075
C22	CA	-0.079
H8	HA	0.129
C25	CA	-0.022
Cl1	Cl	-0.087
C23	CA	-0.053
H9	HA	0.144
C24	CA	-0.225
H10	HA	0.168

1qpe

C13	CT	-0.362
H9	HC	0.137
H10	HC	0.137
H11	HC	0.137
C12	CT	0.111
C14	CT	-0.362
H12	HC	0.137
H13	HC	0.137
H14	HC	0.137
C15	CT	-0.362
H15	HC	0.137
H16	HC	0.137
H17	HC	0.137
N4	N*	-0.003
N3	NA	-0.113
H1	H	0.333
C1	CA	0.340
N1	NC	-0.541
C2	CA	0.526
H4	H5	0.097
N2	NC	-0.641
C3	CA	0.627
N5	NH	-0.832
H2	H	0.414
H3	H	0.414
C4	CA	-0.072
C5	CA	-0.018
C6	CA	0.054
C7	CA	-0.141
H5	HA	0.150
C8	CA	-0.063
H6	HA	0.157
C9	CA	-0.012
Cl1	Cl	-0.032
C10	CA	-0.063
H7	HA	0.157
C11	CA	-0.141
H8	HA	0.150

1r09

C1	CT	0.020
H1	H1	0.092
H2	H1	0.092
H3	H1	0.092
O1	OS	-0.424
C2	CA	0.812
N1	NC	-0.528
N2	NA	0.011
H21	H	0.301
C3	CA	-0.252
H5	HA	0.207
C4	CA	-0.099
H4	HA	0.191
C5	CA	0.173
N3	NH	-0.055
C8	CT	-0.206
H10	H1	0.142
H11	H1	0.142
C9	CT	0.035
H12	H1	0.073
H13	H1	0.073
C6	CT	-0.206
H8	H1	0.142
H9	H1	0.142
C7	CT	0.035
H6	H1	0.073
H7	H1	0.073
N4	NH	-0.286
C10	CA	0.020
C11	CA	-0.075
H14	HA	0.102
C12	CA	0.037
C13	CT	-0.135
H15	HC	0.060
H16	HC	0.060
H17	HC	0.060
C14	CA	-0.098
H18	HA	0.141
C15	CA	-0.227
H19	HA	0.172
C16	CA	-0.141
H20	HA	0.159

1syn

C1	CA	-0.130
H1	HA	0.155
C2	CA	-0.169
H2	HA	0.134
C3	CA	0.017
C4	CA	-0.147
H3	HA	0.152
C5	CA	-0.189
H4	HA	0.136
C6	CA	0.242
N1	NC	-0.615
C7	CD	0.507
C8	CT	-0.162
H5	HC	0.060
H6	HC	0.060
H7	HC	0.060
N2	N	-0.414
H8	H	0.296
C9	C	0.489
O1	O	-0.538
C10	CA	0.011
C11	CA	-0.056
C12	CA	-0.130
H9	HA	0.134
C13	CA	0.013
C14	CT	0.069
H10	H1	0.078
H11	H1	0.078
N3	NH	-0.690
H12	H	0.375
C15	CA	0.106
C18	CA	-0.156
H15	HA	0.166
C16	CA	-0.147
H14	HA	0.097
C17	CA	-0.189
H13	HA	0.139
C21	CA	0.016
C22	C	0.489
O2	O	-0.615
C19	CA	-0.032
C20	CT	-0.079
H21	H1	0.088
H22	H1	0.088
N4	N	-0.063
C23	CT	0.057
H16	H1	0.058
C24	C	0.766
O3	O	-0.810
O4	O	-0.810
C25	CT	-0.027
H17	HC	0.003

H18	HC	0.003
C26	CT	0.040
H19	HC	-0.075
H20	HC	-0.075
C27	C	0.783
O6	O	-0.824
O5	O	-0.824

1thl

C9	CA	-0.140
C8	CA	-0.205
C6	CA	-0.099
H7	HA	0.119
H9	HA	0.124
H10	HA	0.102
C7	CA	-0.205
H8	HA	0.124
C5	CA	-0.099
H6	HA	0.119
C4	CA	0.025
C3	CT	0.022
H4	HC	0.011
H5	HC	0.011
C2	CT	-0.038
H2	HC	-0.012
H3	HC	-0.012
C1	CT	0.071
C10	C	0.720
O1	O	-0.795
O2	O	-0.795
H1	HC	-0.020
C11	CT	0.231
H11	HC	-0.101
H12	HC	-0.101
C12	CT	0.174
C13	CT	0.016
C15	CT	0.006
C16	CT	0.006
C14	CT	0.016
H15	HC	-0.028
H16	HC	-0.028
H19	HC	-0.022
H20	HC	-0.022
H17	HC	-0.022
H18	HC	-0.022
H13	HC	-0.028
H14	HC	-0.028
C17	C	0.328
O3	O	-0.545
N1	N	-0.297
H21	H	0.262
C18	CT	-0.132
C19	C	0.713
O4	O	-0.725
O5	O	-0.725
H22	H1	0.094
C20	CT	-0.124
H23	HC	0.083
H24	HC	0.083
C21	C*	-0.042
C22	CW	-0.101
H25	H4	0.188
N2	NA	-0.389
H26	H	0.345
C24	CN	0.077
C23	CB	0.089
C25	CA	-0.141
H27	HA	0.124
C27	CA	-0.240
H29	HA	0.130
C28	CA	-0.161
H30	HA	0.118
C26	CA	-0.216
H28	HA	0.128

1uvs

C2	CT	-0.003
H7	HC	0.026
H8	HC	0.026
C1	CT	-0.035
H5	HC	0.026
H6	HC	0.026
C6	CT	-0.033
H14	HC	0.025
H15	HC	0.025
C5	CT	-0.035
H12	HC	0.026
H13	HC	0.026
C4	CT	-0.003
H10	HC	0.026
H11	HC	0.026
C3	CT	-0.036
H9	H1	0.153
S1	S6	0.683
O1	O	-0.479
O2	O	-0.479
N1	NT	-0.550
H1	H	0.389
C7	CT	0.003
H16	H1	0.101
H17	H1	0.101
C8	C	0.465
O3	O	-0.552
N2	N	-0.328
H2	H	0.287
C9	CT	0.068
C17	C	0.467
O4	O	-0.558
N5	N	-0.143
C18	CT	0.005
C19	CT	-0.019
C20	CT	-0.026
C21	CT	-0.019
C22	CT	0.005
H35	H1	0.052
H36	H1	0.052
H33	HC	0.020
H34	HC	0.020
H31	HC	0.025
H32	HC	0.025
H29	HC	0.020
H30	HC	0.020
H27	H1	0.052
H28	H1	0.052
H18	H1	0.032
C10	CT	-0.100
H19	HC	0.079
H20	HC	0.079
C11	CT	-0.050
H21	H1	0.119
H22	H1	0.119
N3	NH	-0.444
H3	H	0.337
C12	CA	0.317
C13	CA	-0.162
H23	HA	0.159
C14	CA	-0.069
H24	H4	0.214
N4	NA	-0.123
H4	H	0.326
C15	CA	-0.069
H25	H4	0.214
C16	CA	-0.162
H26	HA	0.159

1uvt

C2	CA	-0.146
H5	HA	0.141
C1	CA	-0.045
H4	H4	0.207
N1	NA	-0.123
H1	H	0.328
C5	CA	-0.045
H7	H4	0.207
C4	CA	-0.146
H6	HA	0.141
C3	CA	0.310
N2	NH	-0.521
H2	H	0.368
C6	CT	-0.008
H8	H1	0.110
H9	H1	0.110
C7	CT	0.131
H10	H1	0.057
H11	H1	0.057
O1	OS	-0.264
C8	CA	0.016
C13	CA	-0.075
C12	CA	-0.017
C11	CA	-0.205
H13	HA	0.176
C14	CT	-0.147
H15	HC	0.072
H16	HC	0.072
H17	HC	0.072
H14	HA	0.116
C9	CA	-0.043
H12	HA	0.149
C10	CA	0.103
N3	NH	-0.571
H3	H	0.375
S1	SY	0.817
O2	O	-0.497
O3	O	-0.497
C15	CA	0.014
C16	CA	-0.096
H18	HA	0.153
C17	CA	-0.160
H19	HA	0.163
C18	CA	-0.075
H20	HA	0.155
C19	CA	-0.160
H21	HA	0.163
C20	CA	-0.096
H22	HA	0.153

1ydr

C2	CA	0.099
C1	CA	-0.359
H1	HA	0.161
C3	CA	0.299
H2	H4	0.085
N1	NC	-0.548
C4	CA	0.263
H3	H4	0.086
C5	CA	0.053
C6	CA	-0.228
H4	HA	0.192
C7	CA	-0.149
H5	HA	0.169
C8	CA	-0.034
H6	HA	0.152
C9	CA	-0.063
S1	SY	0.949
O1	O	-0.490
O2	O	-0.490
N2	NT	-0.412
C10	CT	0.049
C11	CT	0.026
H7	HC	0.026
H8	HC	0.026
H9	HC	0.026
H10	H1	0.088
C12	CT	-0.059
H11	HP	0.110
H12	HP	0.110
N3	N3	-0.140
H13	H	0.284
H18	H	0.284
C13	CT	-0.053
H14	HP	0.107
H15	HP	0.107
C14	CT	0.052
H16	H1	0.109
H17	H1	0.109

1yds

C1	CT	-0.152
H1	HP	0.120
H2	HP	0.120
H3	HP	0.120
N1	N3	-0.040
H4	H	0.277
H16	H	0.277
C2	CT	0.008
H5	HP	0.087
H6	HP	0.087
C3	CT	-0.008
H7	H1	0.109
H8	H1	0.109
N2	NT	-0.674
H9	H	0.409
S1	SY	0.754
O1	O	-0.441
O2	O	-0.441
C4	CA	0.013
C5	CA	-0.038
H10	HA	0.137
C6	CA	-0.166
H11	HA	0.172
C7	CA	-0.200
H12	HA	0.193
C8	CA	0.015
C9	CA	0.259
H13	H4	0.093
N3	NC	-0.526
C10	CA	0.269
H14	H4	0.105
C11	CA	-0.342
H15	HA	0.161
C12	CA	0.135

1ydt

C2	CA	0.121
C1	CA	-0.366
H1	HA	0.172
C3	CA	0.290
H2	H4	0.097
N1	NC	-0.539
C4	CA	0.284
H3	H4	0.083
C5	CA	0.013
C6	CA	-0.214
H4	HA	0.195
C7	CA	-0.165
H5	HA	0.175
C8	CA	-0.048
H6	HA	0.132
C9	CA	0.034
S1	SY	0.794
O1	O	-0.462
O2	O	-0.462
N2	NT	-0.637
H7	H	0.389
C10	CT	0.019
H8	H1	0.090
H9	H1	0.090
C11	CT	0.074
H10	HP	0.061
H11	HP	0.061
N3	N3	-0.132
H12	H	0.280
H21	H	0.280
C12	CT	-0.007
H13	HP	0.105
H14	HP	0.105
C13	CM	-0.221
H15	HA	0.153
C14	CM	-0.106
H16	HA	0.163
C15	CA	0.001
C16	CA	-0.112
H17	HA	0.131
C17	CA	-0.054
H18	HA	0.141
C18	CA	-0.090
Br1	Br	-0.025
C19	CA	-0.054

H19	HA	0.141
C20	CA	-0.112
H20	HA	0.131

2cgr

N1	N1	-0.445
C1	CG	0.353
C2	CA	0.022
C5	CA	-0.157
H3	HA	0.179
C6	CA	-0.113
H4	HA	0.144
C3	CA	-0.157
H2	HA	0.179
C4	CA	-0.113
H1	HA	0.144
C7	CA	0.083
N2	NH	-0.262
H5	H	0.292
C8	C2	0.331
N3	NH	-0.258
C9	CT	-0.086
C10	C	0.730
O1	O	-0.704
O2	O	-0.704
H6	H1	0.062
H7	H1	0.062
H20	H	0.325
N4	NH	-0.482
H8	H	0.310
C11	CT	0.012
H14	H1	0.119
C18	CA	0.080
C19	CA	-0.133
H15	HA	0.135
C20	CA	-0.196
H16	HA	0.158
C21	CA	-0.078
H17	HA	0.135
C22	CA	-0.196
H18	HA	0.158
C23	CA	-0.133
H19	HA	0.135
C12	CA	0.080
C13	CA	-0.133
H13	HA	0.135
C14	CA	-0.196
H12	HA	0.158
C15	CA	-0.078
H11	HA	0.135
C16	CA	-0.196
H10	HA	0.158
C17	CA	-0.133
H9	HA	0.135

2csn

C6	CA	0.000
H5	HA	0.151
C5	CA	-0.115
H4	HA	0.166
C4	CA	-0.085
Cl1	Cl	-0.007
C9	CA	0.242
C3	CA	-0.304
H3	HA	0.155
C2	CA	0.249
H2	H4	0.112
N1	NC	-0.537
C1	CA	0.251
H1	H4	0.120
C8	CA	-0.007
C7	CA	-0.060
S1	SY	0.658
O1	O	-0.443
O2	O	-0.443
N2	NT	-0.765
H6	H	0.454
C10	CT	0.142
H7	H1	0.107
H8	H1	0.107
C11	CT	-0.007
H9	HP	0.095
H10	HP	0.095
N3	N3	-0.157
H11	H	0.276
H12	H	0.276
H13	H	0.276

2pcp

C6	CA	-0.206
C7	CA	-0.088
H1	HA	0.114
H2	HA	0.167
C5	CA	-0.054
H3	HA	0.154
C4	CA	-0.206
H4	HA	0.167
C3	CA	-0.088
H5	HA	0.114
C2	CA	0.098
C8	CT	-0.009
C9	CT	-0.088
C10	CT	-0.011
C11	CT	-0.035
C12	CT	-0.011
C13	CT	-0.088
H12	HC	0.051
H13	HC	0.051
H25	HC	0.037
H26	HC	0.037
H10	HC	0.035
H11	HC	0.035
H8	HC	0.037
H9	HC	0.037
H6	HC	0.051
H7	HC	0.051
N1	N3	-0.136
H14	H	0.305
C14	CT	-0.068
H17	HP	0.108
H18	HP	0.108
C15	CT	-0.060
H19	HC	0.057
H20	HC	0.057
C1	CT	-0.032
H23	HC	0.055
H24	HC	0.055
C16	CT	-0.060
H21	HC	0.057
H22	HC	0.057
C17	CT	-0.068
H15	HP	0.108
H16	HP	0.108

2qwi

O1	O	-0.595
C1	C	0.671
C2	CT	-0.131
H1	HC	0.053
H2	HC	0.053
H3	HC	0.053
N1	N	-0.763
H4	H	0.372
C3	CT	0.043
H5	H1	0.173
C4	CT	0.154
N2	NH	-0.348
C5	CM	0.726
N3	NH	-0.866
H6	H	0.432
H22	H	0.432
N4	NH	-0.866
H7	H	0.432
H8	H	0.432
H9	H	0.304
H10	H1	0.093
C6	CM	-0.424
H23	HA	0.158
C7	CM	0.145
C8	C	0.793
O2	O	-0.741
O3	O	-0.741
O4	OS	-0.252
C9	CT	0.075
H11	H1	0.108
C10	C	0.401
O5	O	-0.490
N5	N	-0.128
C11	CT	-0.119
H12	H1	0.087
H13	H1	0.087
H14	H1	0.087
C12	CT	-0.124
H15	H1	0.099
H16	H1	0.099
C13	CT	-0.003
H17	HC	0.020
H18	HC	0.020
C14	CT	-0.013
H19	HC	0.000
H20	HC	0.000
H21	HC	0.000

3cpa

N1	NT	-1.026
H1	H	0.368
H2	H	0.368
C1	CT	0.208
H3	H1	0.040
H4	H1	0.040
C2	C	0.821
O1	O	-0.718
N2	N	-0.918
H13	H	0.308
C3	CT	0.532
C4	C	0.585
O2	O	-0.718
O3	O	-0.718
H5	H1	-0.033
C5	CT	-0.041
H6	HC	0.017
H7	HC	0.017
C6	CA	0.008
C7	CA	-0.187
H8	HA	0.173
C8	CA	-0.256
H9	HA	0.149
C9	CA	0.285
O4	OH	-0.536
H10	HO	0.352
C10	CA	-0.256
H11	HA	0.149
C11	CA	-0.187
H12	HA	0.173

3erk

C1	CT	0.016
H1	HC	0.051
H2	HC	0.051
C2	CT	-0.083
H3	HP	0.125
H4	HP	0.125
N1	N3	-0.151
H5	H	0.296
H6	H	0.296
C3	CT	-0.083
H7	HP	0.125
H8	HP	0.125
C4	CT	0.016
H9	HC	0.051
H10	HC	0.051
C5	CT	0.087
H11	H1	0.053
N2	N*	0.002
C6	CK	0.033
H12	H5	0.215
N3	NB	-0.434
C7	CC	0.296
C8	CM	0.049
C9	CA	-0.168
C10	CA	-0.216
C11	CA	0.316
C12	CA	-0.216
C13	CA	-0.168
H13	HA	0.164
H14	HA	0.192
F1	F	-0.163
H15	HA	0.192
H16	HA	0.164
C14	C*	-0.128
C15	CM	0.003
N6	NA	-0.072
H21	H	0.213
C16	CM	-0.257
H17	HA	0.152
C17	CA	0.336
H18	H4	0.143
N4	NC	-0.532
C18	CA	0.726
N5	NH	-0.959
H19	H	0.483
H20	H	0.483

3ert

C23	CT	-0.211
H19	HP	0.138
H20	HP	0.138
H21	HP	0.138
N1	N3	0.027
C24	CT	-0.211
H16	HP	0.138
H17	HP	0.138
H18	HP	0.138
H15	H	0.295
C22	CT	-0.132
H24	HP	0.139
H25	HP	0.139
C21	CT	0.015
H22	H1	0.108
H23	H1	0.108
O2	OS	-0.282
C20	CA	0.150
C25	CA	-0.203
C26	CA	-0.082
H10	HA	0.129
H11	HA	0.135
C19	CA	-0.203
H12	HA	0.135
C18	CA	-0.082
H13	HA	0.129
C17	CA	-0.011
C10	CM	-0.054
C11	CA	0.022
C12	CA	-0.142
C13	CA	-0.258
C14	CA	0.336
O1	OH	-0.542
H14	HO	0.398
C15	CA	-0.258
C16	CA	-0.142
H3	HA	0.153
H4	HA	0.166
H1	HA	0.166
H2	HA	0.153
C3	CM	-0.041
C2	CT	-0.050
C1	CT	-0.076
H28	HC	0.026
H29	HC	0.026
H30	HC	0.026
H26	HC	0.054
H27	HC	0.054
C4	CA	0.054
C5	CA	-0.066
H5	HA	0.110
C6	CA	-0.213
H6	HA	0.153

C7	CA	-0.101
H7	HA	0.141
C8	CA	-0.213
H8	HA	0.153
C9	CA	-0.066
H9	HA	0.110

3std

N1	N1	-0.348
C1	CG	0.202
C2	CA	0.375
N2	NC	-0.313
N3	NA	-0.001
H21	H	0.329
C3	CA	0.118
C4	CA	-0.276
H1	HA	0.193
C5	CA	-0.019
H2	HA	0.179
C6	CA	-0.129
H3	HA	0.171
C7	CA	-0.142
H4	HA	0.122
C8	CA	0.102
C9	CA	0.076
N4	NH	-0.288
H5	H	0.274
C10	CT	0.011
H6	H1	0.094
H7	H1	0.094
C11	CT	-0.066
H8	HC	0.062
H9	HC	0.062
C12	CT	-0.017
H10	HC	0.068
C19	CA	0.006
C20	CA	-0.105
H16	HA	0.119
C21	CA	-0.179
H17	HA	0.154
C22	CA	-0.088
H18	HA	0.138
C23	CA	-0.179
H19	HA	0.154
C24	CA	-0.105
H20	HA	0.119
C13	CA	0.006
C14	CA	-0.105
H11	HA	0.119
C15	CA	-0.179
H12	HA	0.154
C16	CA	-0.088
H13	HA	0.138
C17	CA	-0.179
H14	HA	0.154
C18	CA	-0.105
H15	HA	0.119

3tmn

C4	CT	-0.277
H5	HC	0.084
H6	HC	0.084
H7	HC	0.084
C3	CT	0.172
C5	CT	-0.277
H8	HC	0.084
H9	HC	0.084
H10	HC	0.084
H4	HC	-0.007
C1	CT	0.029
N1	N3	-0.316
H1	H	0.284
H2	H	0.284
H11	H	0.284
H3	HP	0.093
C2	C	0.513
O1	O	-0.577
N2	N	-0.374
H12	H	0.254
C6	CT	-0.004
C7	C	0.708
O2	O	-0.712
O3	O	-0.712
H13	H1	0.108
C8	CT	-0.125
H14	HC	0.080
H15	HC	0.080
C9	C*	-0.091
C10	CW	-0.047
H16	H4	0.160
N3	NA	-0.687
H17	H	0.410
C12	CN	0.279
C11	CB	0.068
C13	CA	-0.115
H18	HA	0.195
C15	CA	-0.234
H20	HA	0.166
C16	CA	-0.136
H21	HA	0.152
C14	CA	-0.286
H19	HA	0.149

4dfr

N1	NC	-0.364
C1	CA	0.218
C2	CA	0.346
N2	NH	-0.557
H1	H	0.344
H21	H	0.344
N3	NC	-0.548
C3	CA	0.817
N4	NH	-0.934
H2	H	0.424
H3	H	0.424
N5	NA	-0.655
H4	H	0.429
C4	CA	0.588
N6	NC	-0.586
C5	CA	0.262
H5	H4	0.092
C6	CA	0.037
C7	CT	-0.102
H6	H1	0.109
H7	H1	0.109
N7	NH	-0.339
C8	CT	-0.077
H8	H1	0.067
H9	H1	0.067
H10	H1	0.067
C9	CA	0.061
C12	CA	-0.097
H13	HA	0.124
C13	CA	-0.171
H14	HA	0.153
C10	CA	-0.097
H12	HA	0.124
C11	CA	-0.171
H11	HA	0.153
C14	CA	-0.014
C15	C	0.434
O1	O	-0.568
N8	N	-0.211
H15	H	0.253
C16	CT	0.001
C17	C	0.779
O2	O	-0.756
O3	O	-0.756
H16	H1	0.029
C18	CT	-0.031
H17	HC	0.022
H18	HC	0.022
C19	CT	-0.094
H19	HC	0.004
H20	HC	0.004
C20	C	0.725
O5	O	-0.751
O4	O	-0.751

4std

F1	F	-0.192
C5	CA	0.218
C6	CA	-0.160
H9	HA	0.176
C4	CA	-0.151
H8	HA	0.174
C3	CA	-0.292
H7	HA	0.179
C2	CA	0.176
O2	OH	-0.498
H6	HO	0.427
C1	CA	-0.033
C7	C	0.468
O1	O	-0.543
N1	N	-0.228
H1	H	0.158
C8	CT	0.036
C9	CT	-0.131
H3	HC	0.048
H4	HC	0.048
H5	HC	0.048
H2	H1	0.088
C10	CA	0.024
C11	CA	-0.145
H13	HA	0.142
C12	CA	-0.067
H12	HA	0.133
C13	CA	-0.076
Br1	Br	-0.089
C14	CA	-0.067
H11	HA	0.133
C15	CA	-0.145
H10	HA	0.142

5std

C1	CA	-0.360
H1	HA	0.240
C2	CA	0.336
F1	F	-0.140
C3	CA	0.316
F2	F	-0.158
C4	CA	-0.431
H2	HA	0.263
C5	CA	0.086
C6	CA	0.060
N1	NA	-0.120
H20	H	0.307
C7	CA	0.099
H3	H4	0.191
N2	NC	-0.245
C8	CA	0.308
N3	NH	-0.215
H4	H	0.250
C9	CT	-0.098
C10	CT	-0.042
H5	HC	0.039
H6	HC	0.039
H7	HC	0.039
H8	H1	0.125
C11	CT	-0.050
H14	HC	0.046
C18	CA	0.050
C19	CA	-0.114
H15	HA	0.120
C20	CA	-0.191
H16	HA	0.160
C21	CA	-0.088
H17	HA	0.145
C22	CA	-0.191
H18	HA	0.160
C23	CA	-0.114
H19	HA	0.120
C12	CA	0.050
C13	CA	-0.114
H13	HA	0.120
C14	CA	-0.191
H12	HA	0.160
C15	CA	-0.088
H11	HA	0.145
C16	CA	-0.191
H10	HA	0.160
C17	CA	-0.114
H9	HA	0.120

5tln

O6	O	-0.474
N5	NO	0.746
O7	O	-0.474
C20	CA	0.059
C21	CA	-0.217
C16	CA	-0.128
H19	HA	0.165
H22	HA	0.181
C19	CA	-0.217
H21	HA	0.181
C18	CA	-0.128
H20	HA	0.165
C17	CA	0.091
N4	N	-0.227
H18	H	0.149
C1	C	0.576
O1	O	-0.550
C2	CT	-0.081
H1	H1	0.088
H2	H1	0.088
N1	N	-0.198
H3	H	0.235
C3	C	0.368
O2	O	-0.548
C4	CT	0.186
C5	CT	-0.190
H4	HC	0.060
H5	HC	0.060
H6	HC	0.060
H7	H1	0.098
N2	N	-0.574
H8	H	0.246
C6	C	0.567
O3	O	-0.584
C7	CT	0.044
C8	C	0.361
N3	N	-0.140
O4	OH	-0.686
H9	H	0.240
O5	O	-0.563
H10	HC	-0.010
C9	CT	0.035
H11	HC	0.003
H12	HC	0.003
C10	CA	-0.024
C11	CA	-0.138
H13	HA	0.149
C12	CA	-0.174
H14	HA	0.137
C13	CA	-0.123
H15	HA	0.129
C14	CA	-0.174
H16	HA	0.137
C15	CA	-0.138
H17	HA	0.149

7dfr

N1	NC	-0.374
C1	CA	0.170
C2	C	0.472
O1	O	-0.513
N2	NA	-0.292
H1	H	0.326
C3	CA	0.445
N3	NH	-0.838
H2	H	0.434
H3	H	0.434
N4	NA	-0.309
H18	H	0.303
C4	CA	0.408
N5	NC	-0.490
C5	CA	0.257

H4	H4	0.108
C6	CA	0.089
C7	CT	0.004
H5	H1	0.086
H6	H1	0.086
N6	NH	-0.644
H7	H	0.362
C8	CA	0.099
C11	CA	-0.152
H10	HA	0.148
C12	CA	-0.141
H11	HA	0.143
C9	CA	-0.152
H8	HA	0.148
C10	CA	-0.141
H9	HA	0.143
C13	CA	0.062
C14	C	0.353
O2	O	-0.577
N7	N	-0.107
H12	H	0.164
C15	CT	0.018
C16	C	0.726
O3	O	-0.772
O4	O	-0.772
H13	H1	0.037
C17	CT	0.109
H14	HC	-0.012
H15	HC	-0.012
C18	CT	-0.332
H16	HC	0.083
H17	HC	0.083
C19	C	0.740
O6	O	-0.706
O5	O	-0.706

7est

F1	F	-0.145
C2	CT	0.392
F2	F	-0.145
F3	F	-0.145
C1	C	0.436
O1	O	-0.495
N1	N	-0.274
H1	H	0.242
C3	CT	0.015
C5	CT	-0.152
C6	CT	0.108
C7	CT	-0.047
H6	HC	0.010
H7	HC	0.010
H8	HC	0.010
C8	CT	-0.047
H9	HC	0.010
H10	HC	0.010
H11	HC	0.010
H5	HC	0.010
H3	HC	0.066
H4	HC	0.066
H2	H1	0.106
C4	C	0.268
O2	O	-0.448
N2	N	-0.195
H12	H	0.238
C9	CT	0.089
C11	CT	-0.184
H14	HC	0.064
H15	HC	0.064
H16	HC	0.064
H13	H1	0.080
C10	C	0.445
O3	O	-0.507
N3	N	-0.177
H17	H	0.116
C12	CA	0.075
C13	CA	-0.132
H18	HA	0.167
C14	CA	-0.194
H19	HA	0.164
C15	CA	-0.022
C18	CT	0.606
F4	F	-0.212
F5	F	-0.212
F6	F	-0.212
C16	CA	-0.194
H20	HA	0.164
C17	CA	-0.132
H21	HA	0.167

830c

C4	CA	-0.193
H14	HA	0.152
C3	CA	-0.098
H13	HA	0.155
C2	CA	-0.016
Cl1	Cl	-0.132
C1	CA	-0.098
H12	HA	0.155
C6	CA	-0.193
H15	HA	0.152
C5	CA	0.272
O1	OS	-0.355
C7	CA	0.259
C12	CA	-0.159
C11	CA	-0.163
H17	HA	0.178
H18	HA	0.146
C8	CA	-0.159
H16	HA	0.146
C9	CA	-0.163
H19	HA	0.178
C10	CA	-0.030
S1	SY	0.985
O2	O	-0.599
O3	O	-0.599
C13	CT	-0.018
H1	H1	0.014
H2	H1	0.014
C14	CT	0.042
C19	C	0.549
N1	N	-0.232
O6	OH	-0.713
H11	H	0.280
O5	O	-0.665
C15	CT	-0.068
H3	HC	0.029
H4	HC	0.029
C16	CT	0.159
H5	H1	0.011
H6	H1	0.011
O4	OS	-0.436
C17	CT	0.159
H7	H1	0.011
H8	H1	0.011
C18	CT	-0.068
H9	HC	0.029
H10	HC	0.029

966c

C1	CA	-0.198
H1	HA	0.119
C2	CA	-0.176
H2	HA	0.138
C3	CA	-0.159
H3	HA	0.123
C4	CA	-0.176
H4	HA	0.138
C5	CA	-0.198
H5	HA	0.119
C6	CA	0.442
O1	OS	-0.572
C7	CA	0.605
C10	CA	-0.317
H8	HA	0.179
C11	CA	-0.151
H9	HA	0.178
C8	CA	-0.317
H7	HA	0.179
C9	CA	-0.151
H6	HA	0.178
C12	CA	0.023
S1	SY	0.765
O2	O	-0.569
O3	O	-0.569
C13	CT	0.183
C14	CT	-0.281
C15	C	0.655
O4	O	-0.614
N1	N	-0.510
O5	OH	-0.510
H10	H	0.326
H11	HC	0.057
H12	HC	0.057
C16	CT	0.030
H13	HC	0.011
H14	HC	0.011
C17	CT	0.088
H15	H1	0.037
H16	H1	0.037
O6	OS	-0.428
C18	CT	0.088
H17	H1	0.037
H18	H1	0.037
C19	CT	0.030
H19	HC	0.011
H20	HC	0.011
