# Supplementary material for: Preference of Small Molecules for Local Minimum Conformations when Binding to Proteins
Source: PLoS One. 2007 Sep 5;2(9):e820. doi: 10.1371/journal.pone.0000820 (PMC1959118; doi:10.1371/journal.pone.0000820)
Supplement: Figure S1 — Perl script for the normal-mode-analysis-monitored energy minimization procedure. (0.05 MB PDF) [file pone.0000820.s006.pdf]

Figure S1. Perl script of the NMODE-monitored energy minimization procedure.

all.pl

```
#! /usr/bin/perl

## Perl script of NMODE-monitored energy minimization procedure ##

use Getopt::Long;

$label = 10;
$i = 0;

&GetOptions(
'label|label=s' => \$label,
'i|i=s' => \$i,
);

   system ("cp ../para/prm??? .");
   system ("rm rms");
   system ("rm *.log");
   $end = 0;
   $num = 0;

   system ("min.pl -l $label >> min.log");
   system ("rm CHECK");
   system ("nmode.pl -l $label -i $i >> nmode.log");

   while ($end == 0) {
       if (open (IN, "CHECK") ) {
           system ("cp $label.restrt prmcrd");
           system ("min.pl -l $label >> min.log");
           system ("rm CHECK");
           system ("nmode.pl -l $label -i $i >> nmode.log");
           system ("mv $label.nmode.out $label.$num.nmode.out");
           $num++; print("$num\n");
       }
       else {
       $end = 1;
       print ("The minimization has reached a local minimum!!\n");
       }
    }
```

min.pl

```
#! /usr/bin/perl

## Perl script for minimization ##

use Getopt::Long;

$label = 10;

&GetOptions(
'label|label=s' => \$label,
);

   system ("sed 's/maxcyc =10/maxcyc =$label/g' ../../min.in > tmpmin.in");

   $end = 0;
   $num = 0;
   while ( $end == 0 )
   {
   $num++;
   system ("$amber5/exe/sander-large -O -i tmpmin.in -o min.out -c prmcrd -p prmtop -r restrt");
   system ("grep '  $label  ' min.out | tail -1 > tmp");
   open  (IN,  "tmp")  ||  die  "  cannot  open  tmp,  does  file  tmp  exist?  Sth  wrong  with  the
minimization.\n";
   while (<IN>)
   {
   $_ = ~/(\s+)(\d+)(\s+)(\S+)(\s+)(\S{1})(\S{6})(\S{1})(\S{2})/ ;
   if ($6 < 6 && $8 eq "-" && $9 > 1) {
```

```
      $end = 1;
      }
   elsif ( $8 eq "-" && $9 > 2) {
      $end = 1;
      }
   else
      {system ("cp restrt prmcrd");}
   }
   }
   close (IN);

   system ("mv restrt $label.restrt");
   system ("mv min.out $label.step$num.out");
```

nmode.pl

```
#! /usr/bin/perl

## Perl script for normal mode analysis ##

use Getopt::Long;

$label = 10;
$i = 0;

&GetOptions(
'label|label=s' => \$label,
'i|i=s' => \$i,
);

   system ("$amber8/exe/nmode -O -i ../../nmode$i.in -o $label.nmode.out -c $label.restrt -p
prmtop -r rst");

   system ("grep '     1   ' $label.nmode.out > all.check");
   system ("grep '     4   ' $label.nmode.out >> all.check");
   system ("grep '     5   ' $label.nmode.out >> all.check");
   system ("grep '     6   ' $label.nmode.out >> all.check");
   system ("grep '     7   ' $label.nmode.out >> all.check");

   system ("tail -5 all.check > $label.check");

   open (IN, "$label.check") || die " cannot open check, does file exist? Sth wrong with the
minimization.
";
   while (<IN>)
    {
      if ( $_ =~/(\s+)(\d)(\s+)(\S+)/ ) {
      if ( ($2 == 1 && $4 <= -0.01) || ($2 == 6 && $4 > 10 ) || ($2 == 7 && $4 <= 0 ) ){
      open (OUT, ">>CHECK");
      print OUT ("$label is not a minimum!!\n");
      print ("$label is not a minimum!! The number has been written to file CHECK!!\n");
      close (OUT);
      }
      elsif ($1.$2.$3.$4 eq "          1         0") {
      open (OUT, ">>CHECK");
      print OUT ("$label cannot go through nmode analysis!!\n");
      print ("$label cannot go through nmode analysis!! The number has been written to file
CHECK!!\n");
      close (OUT);
      }
      }
    }

   close (IN);

   system ("cp $label.restrt restrt");
   system ("$amber8/exe/ptraj prmtop ../../rms.trajin");
   system ("echo $label >> rms");
   system ("tail -1 rms0.dat >> rms");

   system ("$amber8/exe/ptraj prmtop ../../pdb.trajin");
   system ("mv restrt.pdb.1 $label.pdb");
```
