# Supplementary figures and images for: Preference of Small Molecules for Local Minimum Conformations when Binding to Proteins
Source: PLoS One. 2007 Sep 5;2(9):e820. doi: 10.1371/journal.pone.0000820 (PMC1959118; doi:10.1371/journal.pone.0000820)

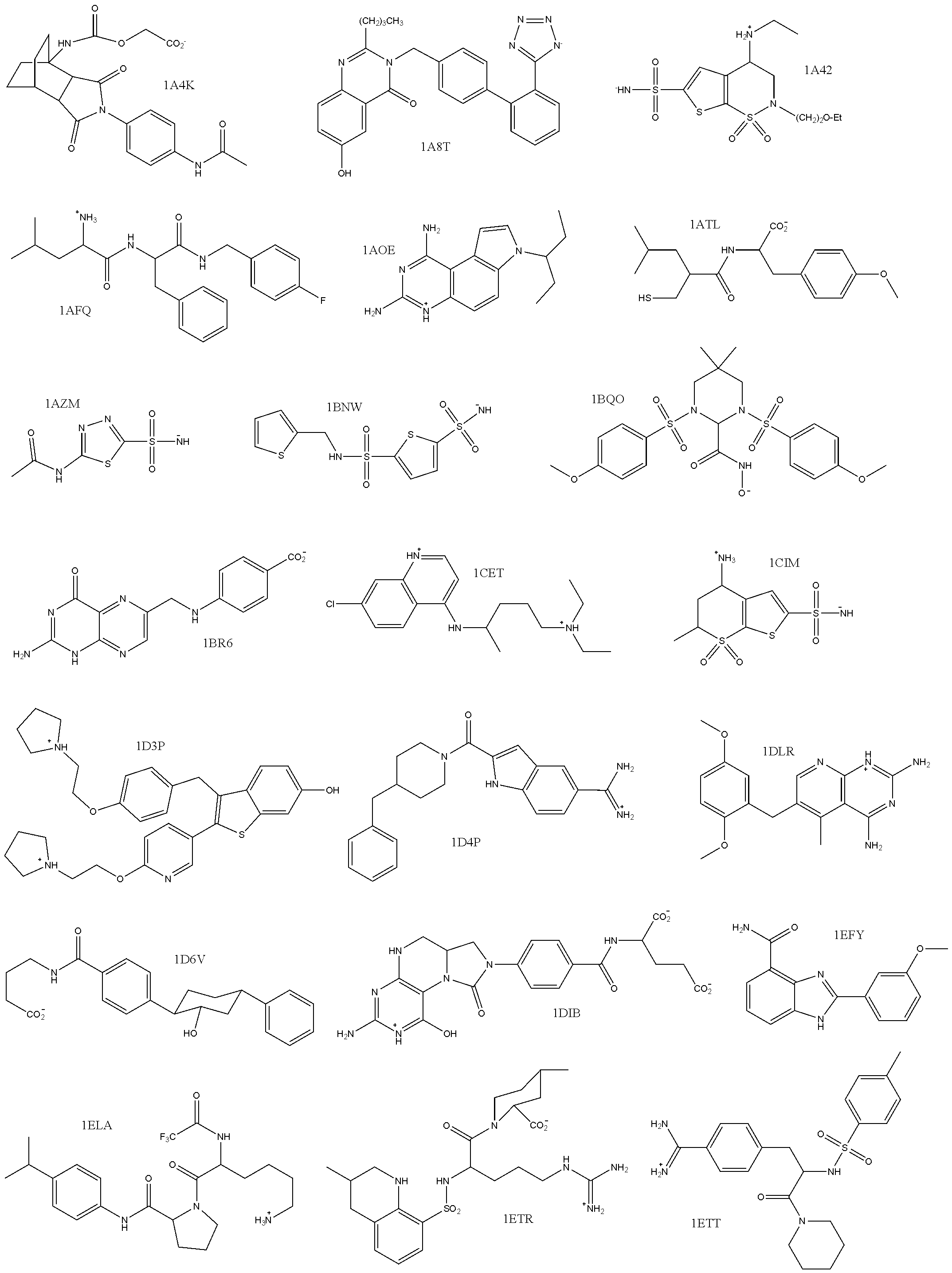

Supplement: Figure S2 — Chemical structures and protonation states of the 100 protein-bound ligands (Part I). (6.16 MB TIF) [file pone.0000820.s007.tif]

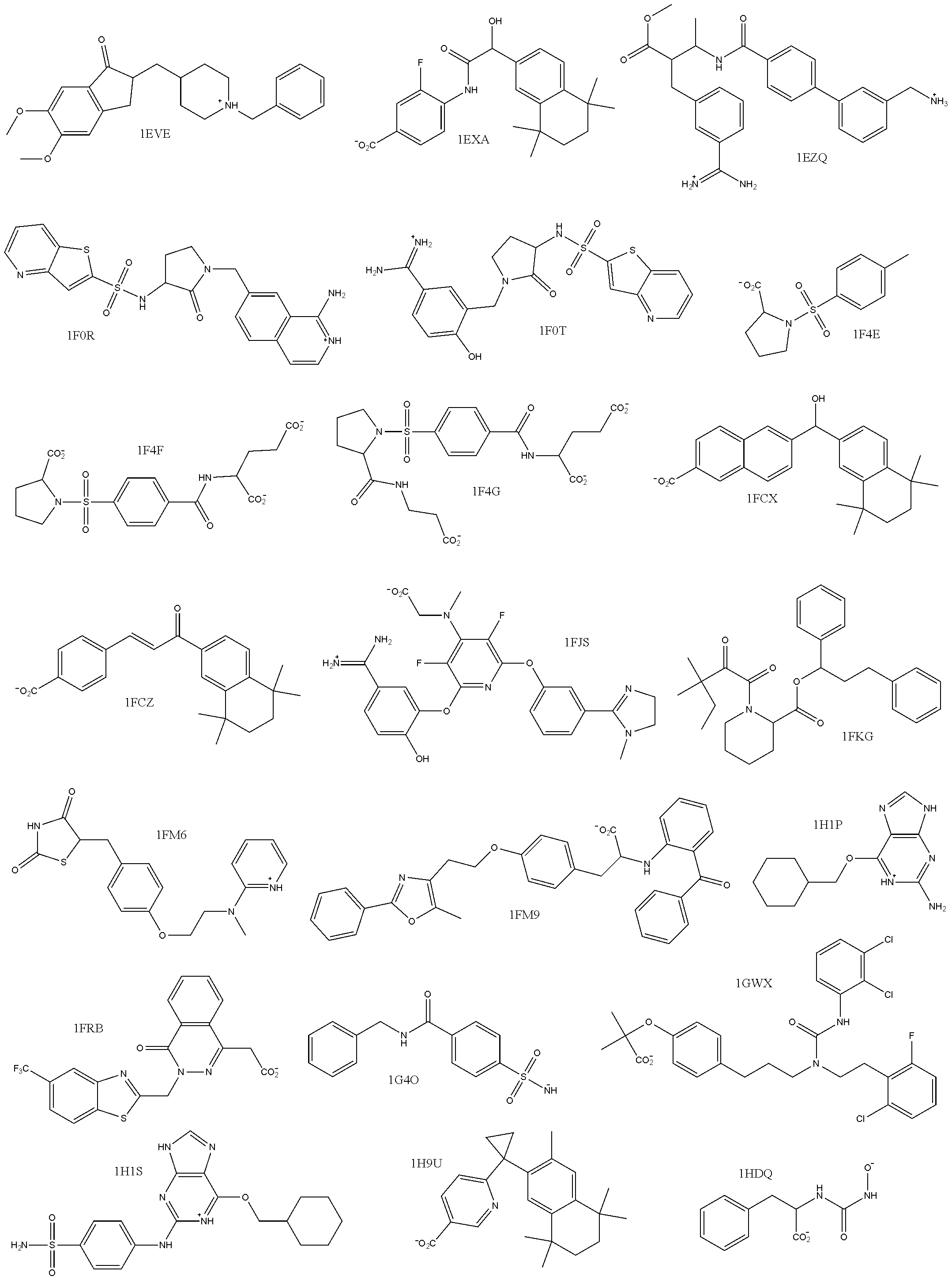

Supplement: Figure S3 — Chemical structures and protonation states of the 100 protein-bound ligands (Part II). (6.25 MB TIF) [file pone.0000820.s008.tif]

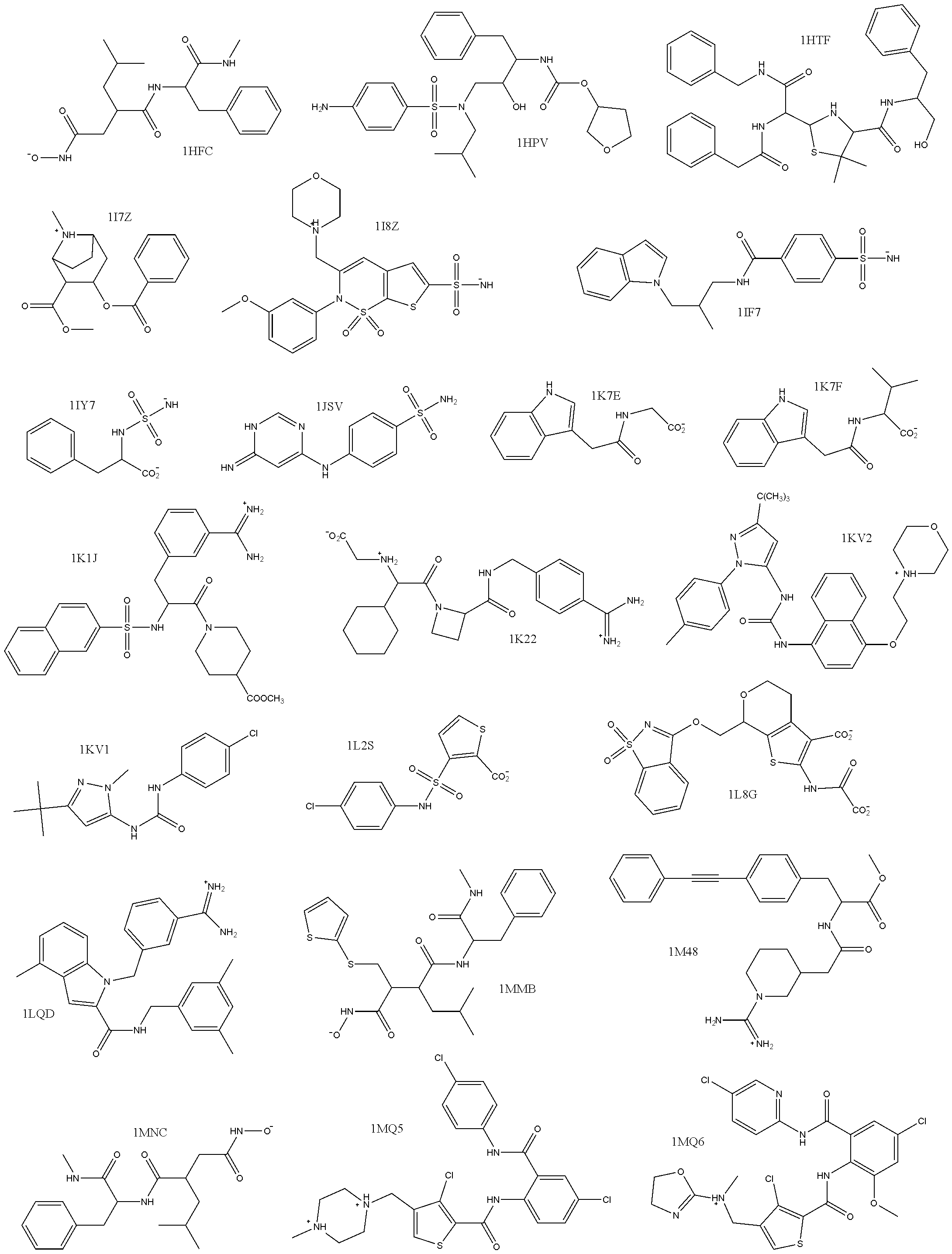

Supplement: Figure S4 — Chemical structures and protonation states of the 100 protein-bound ligands (Part III). (6.26 MB TIF) [file pone.0000820.s009.tif]

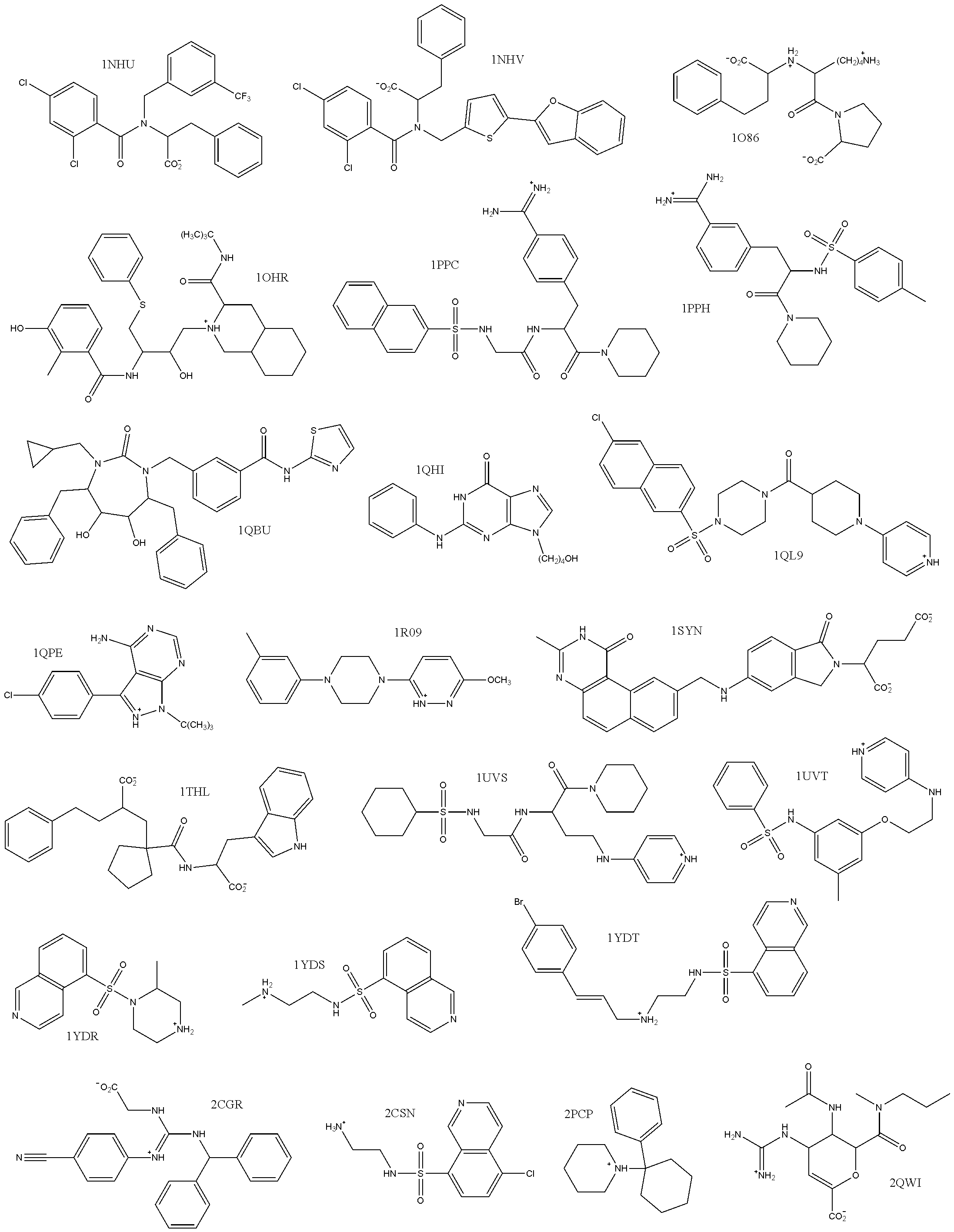

Supplement: Figure S5 — Chemical structures and protonation states of the 100 protein-bound ligands (Part IV). (6.21 MB TIF) [file pone.0000820.s010.tif]

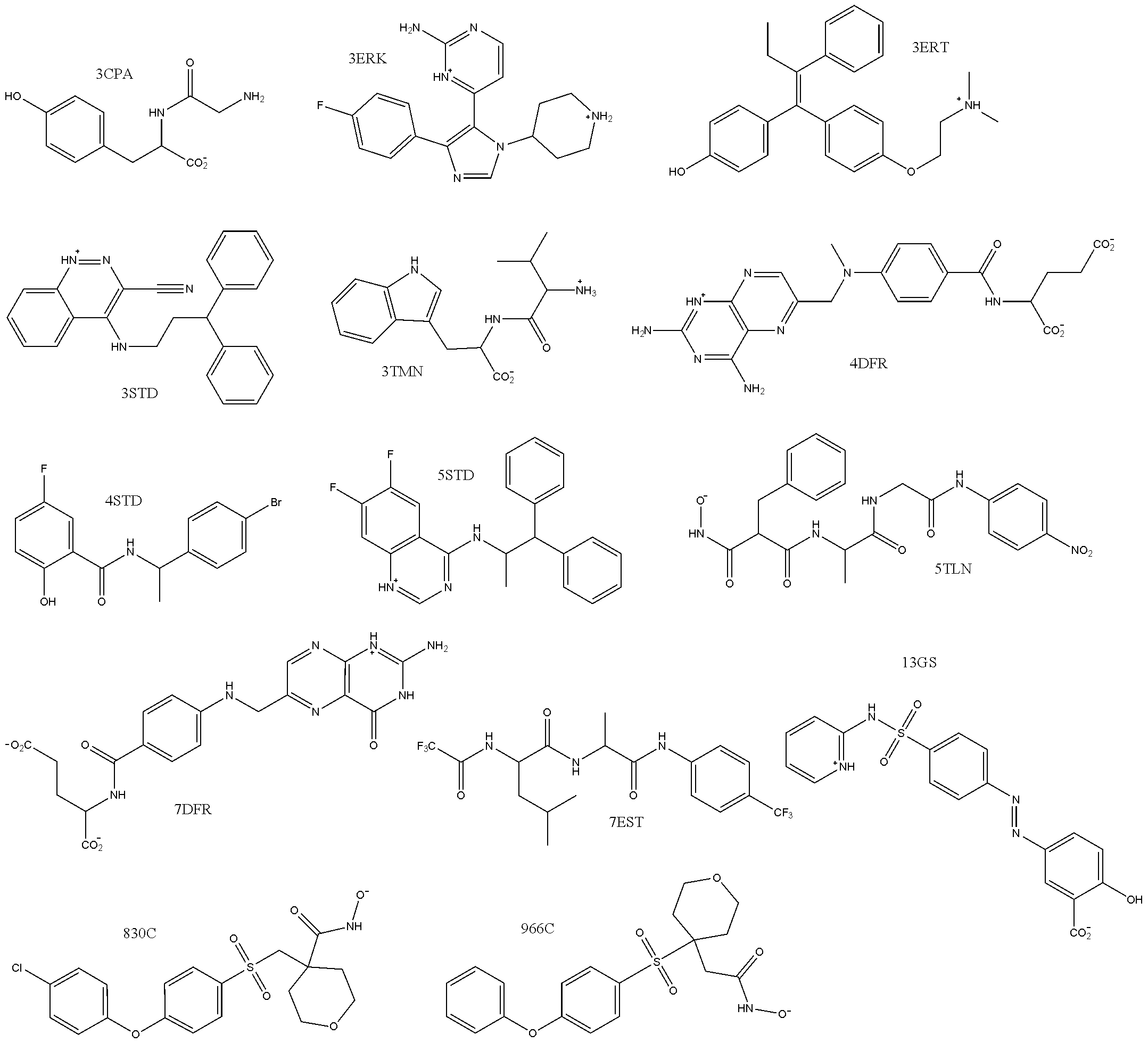

Supplement: Figure S6 — Chemical structures and protonation states of the 100 protein-bound ligands (Part V). (4.23 MB TIF) [file pone.0000820.s011.tif]

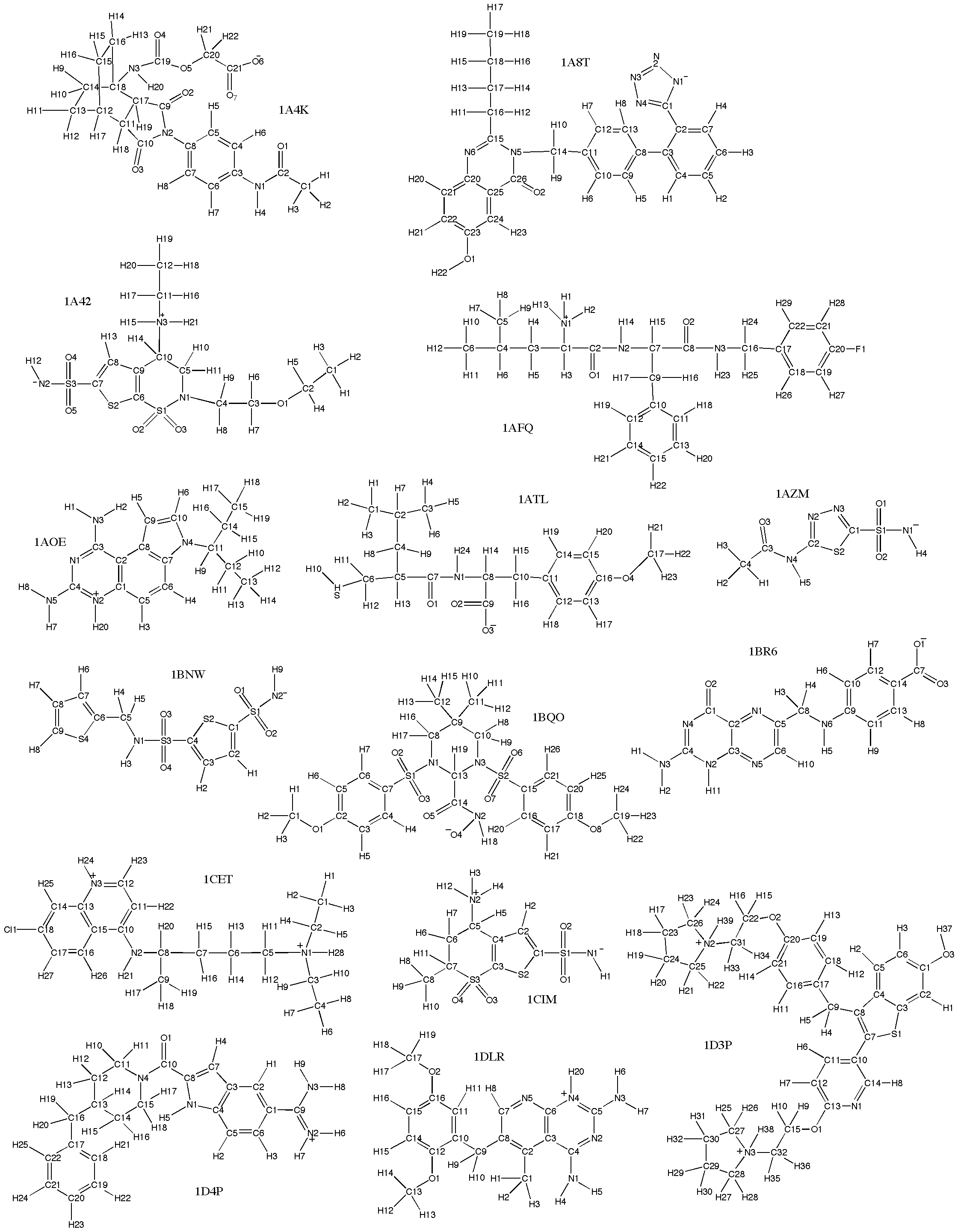

Supplement: Figure S7 — Definitions of atom labels of the 100 protein-bound ligands (Part I). (6.46 MB TIF) [file pone.0000820.s012.tif]

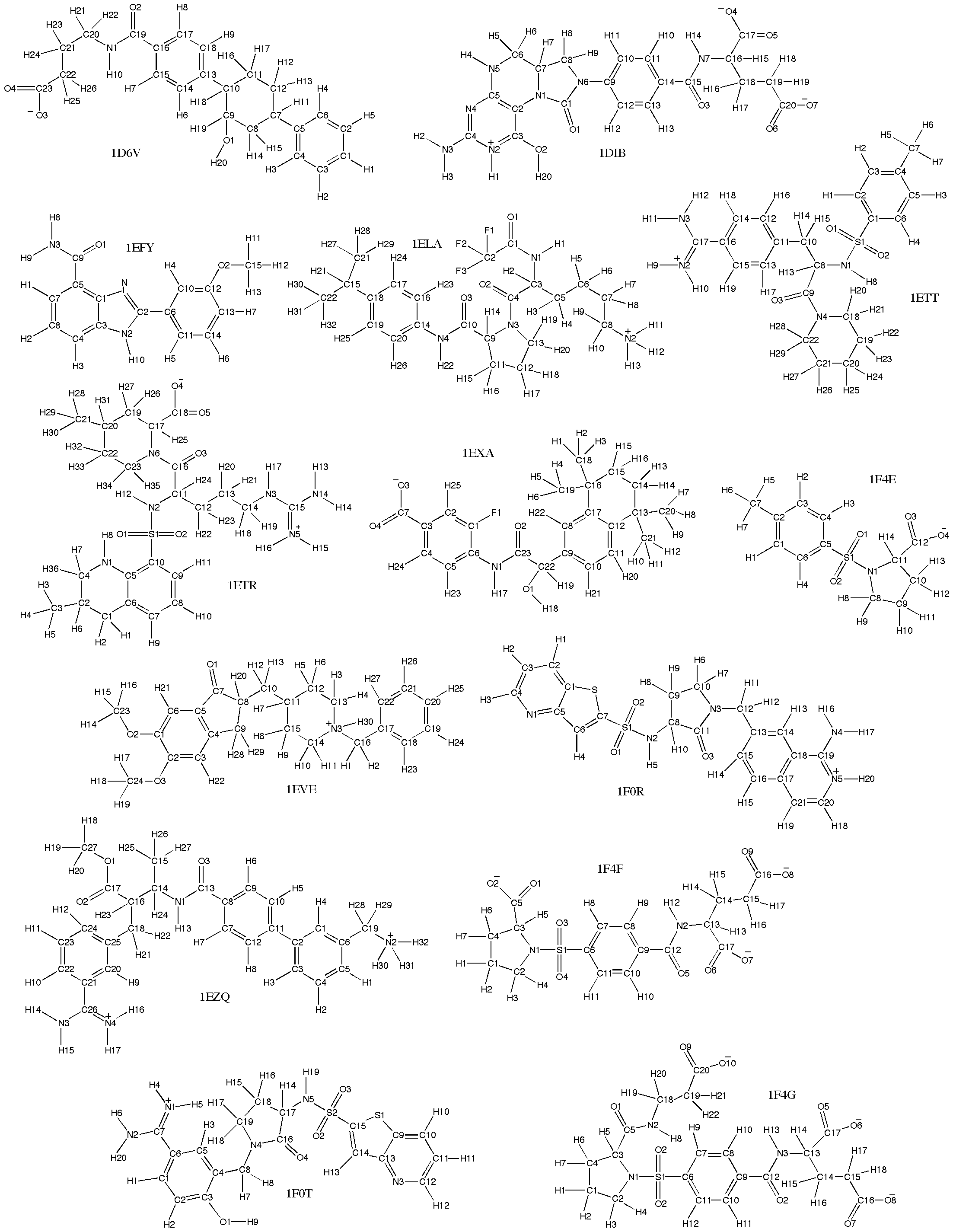

Supplement: Figure S8 — Definitions of atom labels of the 100 protein-bound ligands (Part II). (6.28 MB TIF) [file pone.0000820.s013.tif]

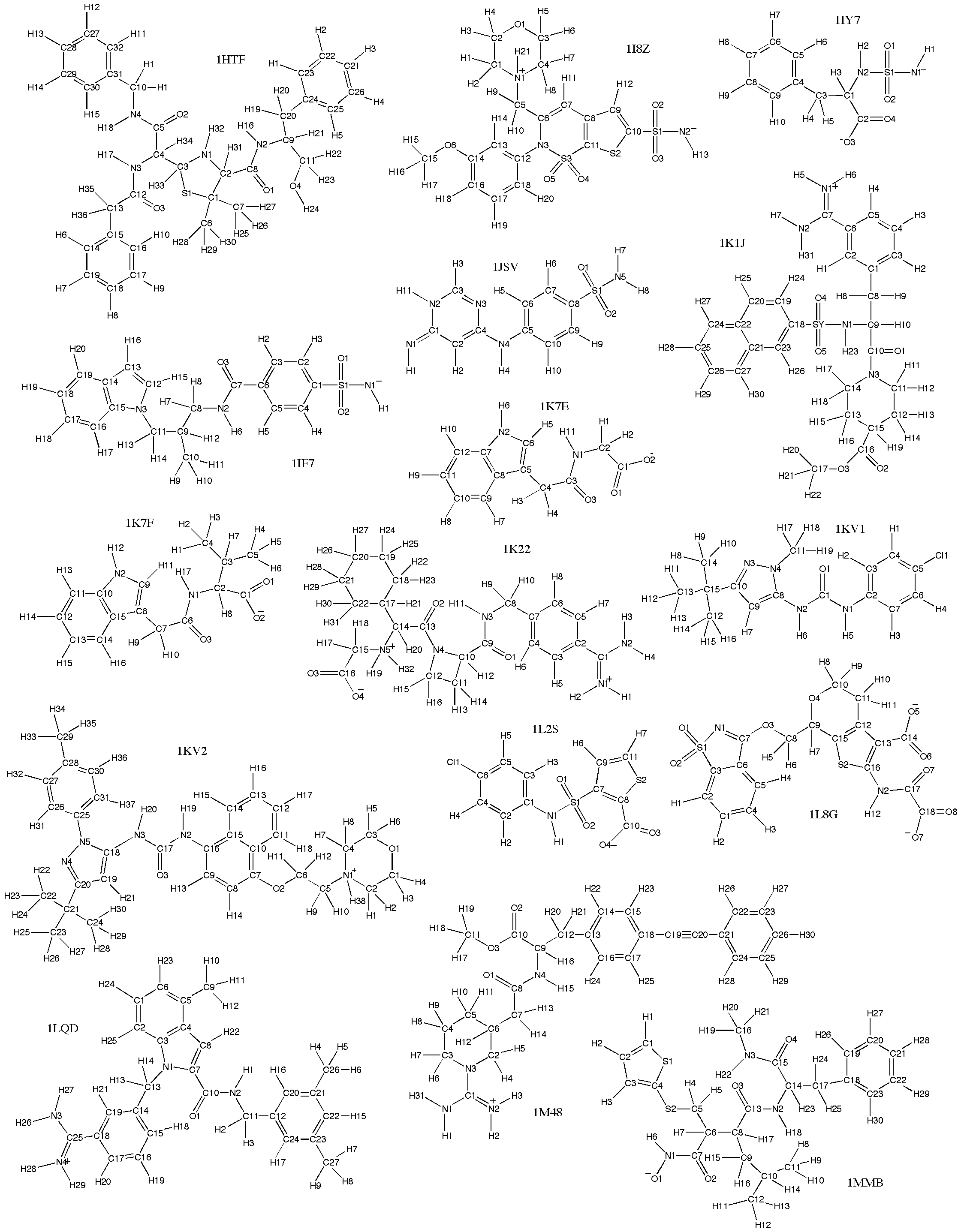

Supplement: Figure S10 — Definitions of atom labels of the 100 protein-bound ligands (Part IV). (6.44 MB TIF) [file pone.0000820.s015.tif]

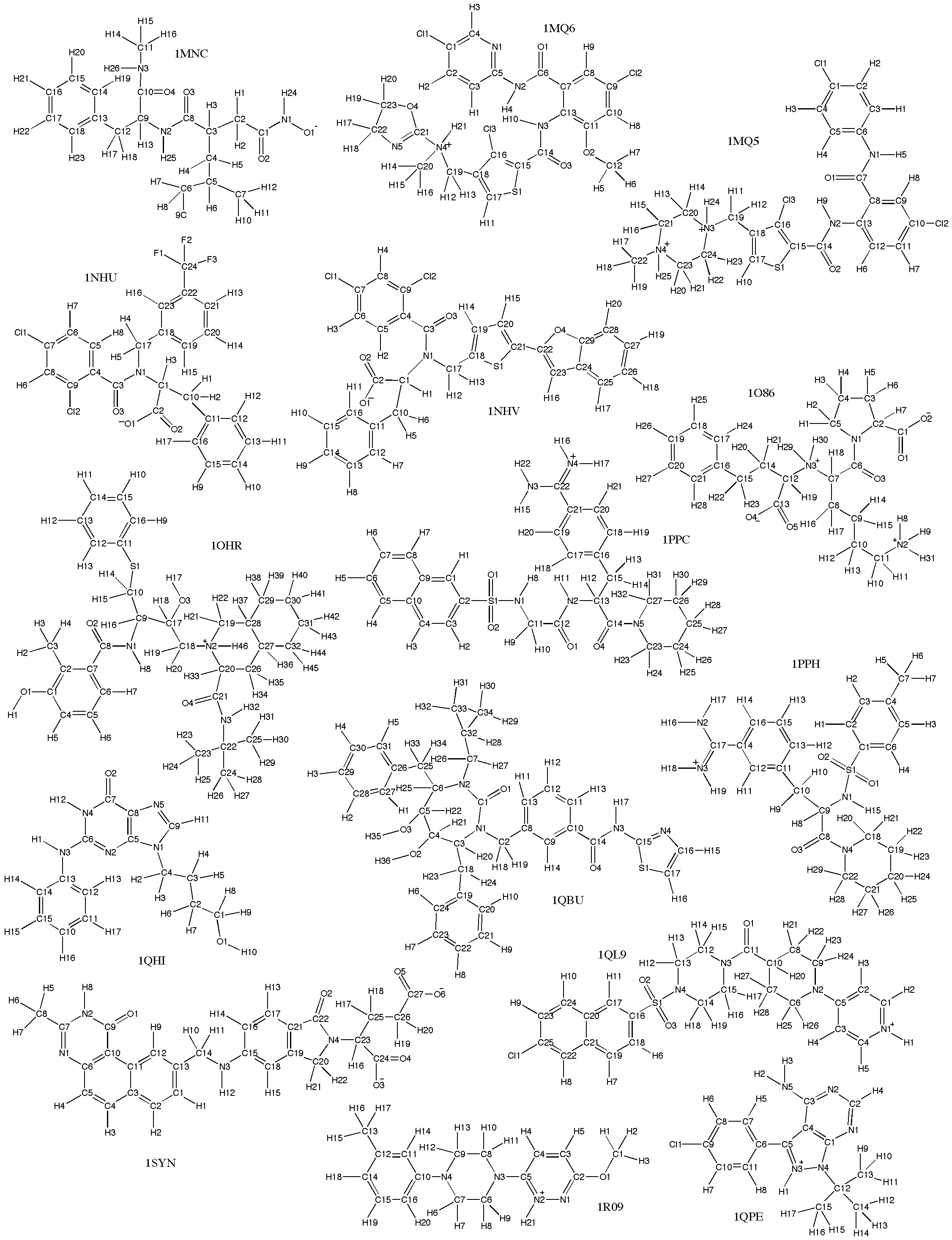

Supplement: Figure S11 — Definitions of atom labels of the 100 protein-bound ligands (Part V). (6.61 MB TIF) [file pone.0000820.s016.tif]

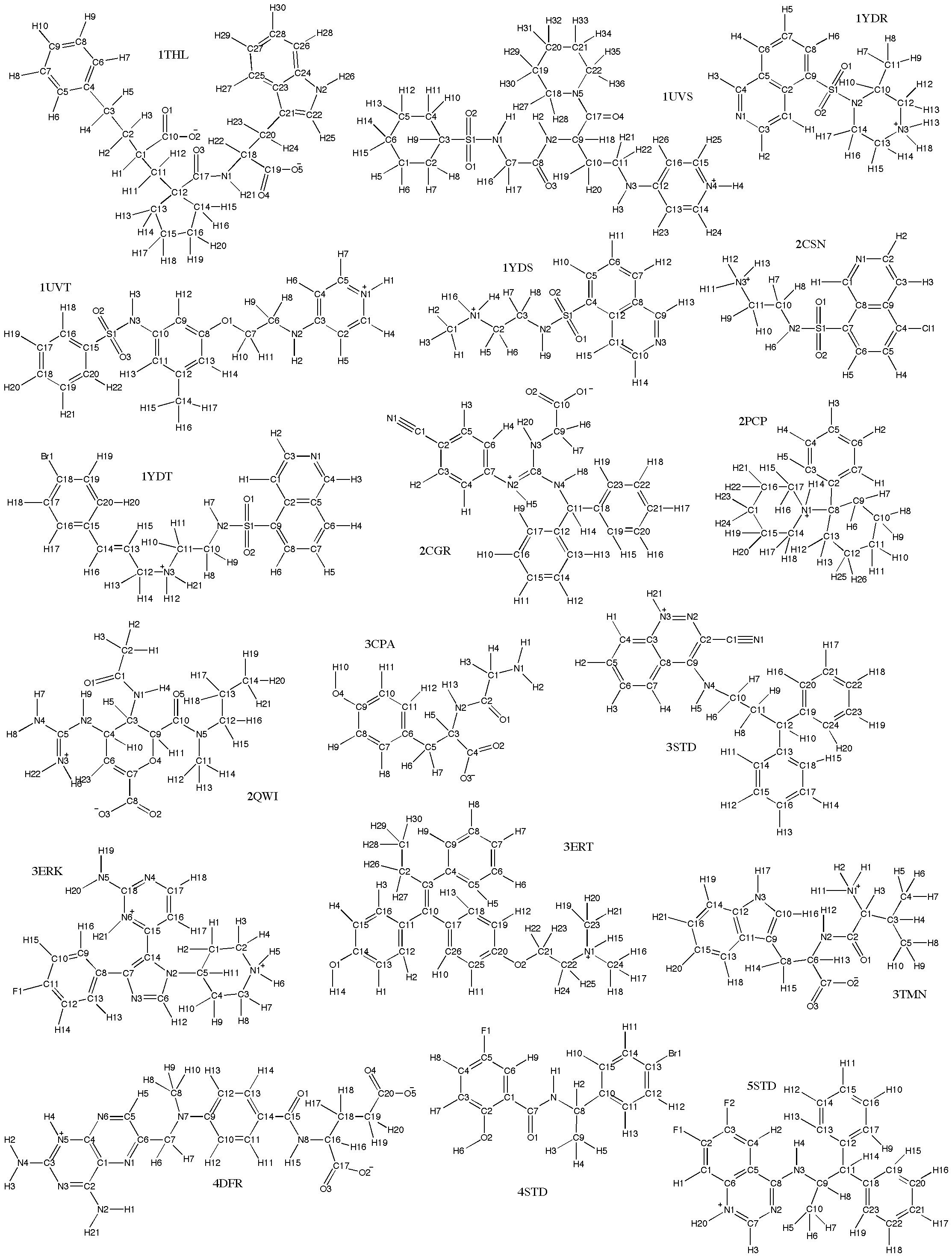

Supplement: Figure S12 — Definitions of atom labels of the 100 protein-bound ligands (Part VI). (6.55 MB TIF) [file pone.0000820.s017.tif]

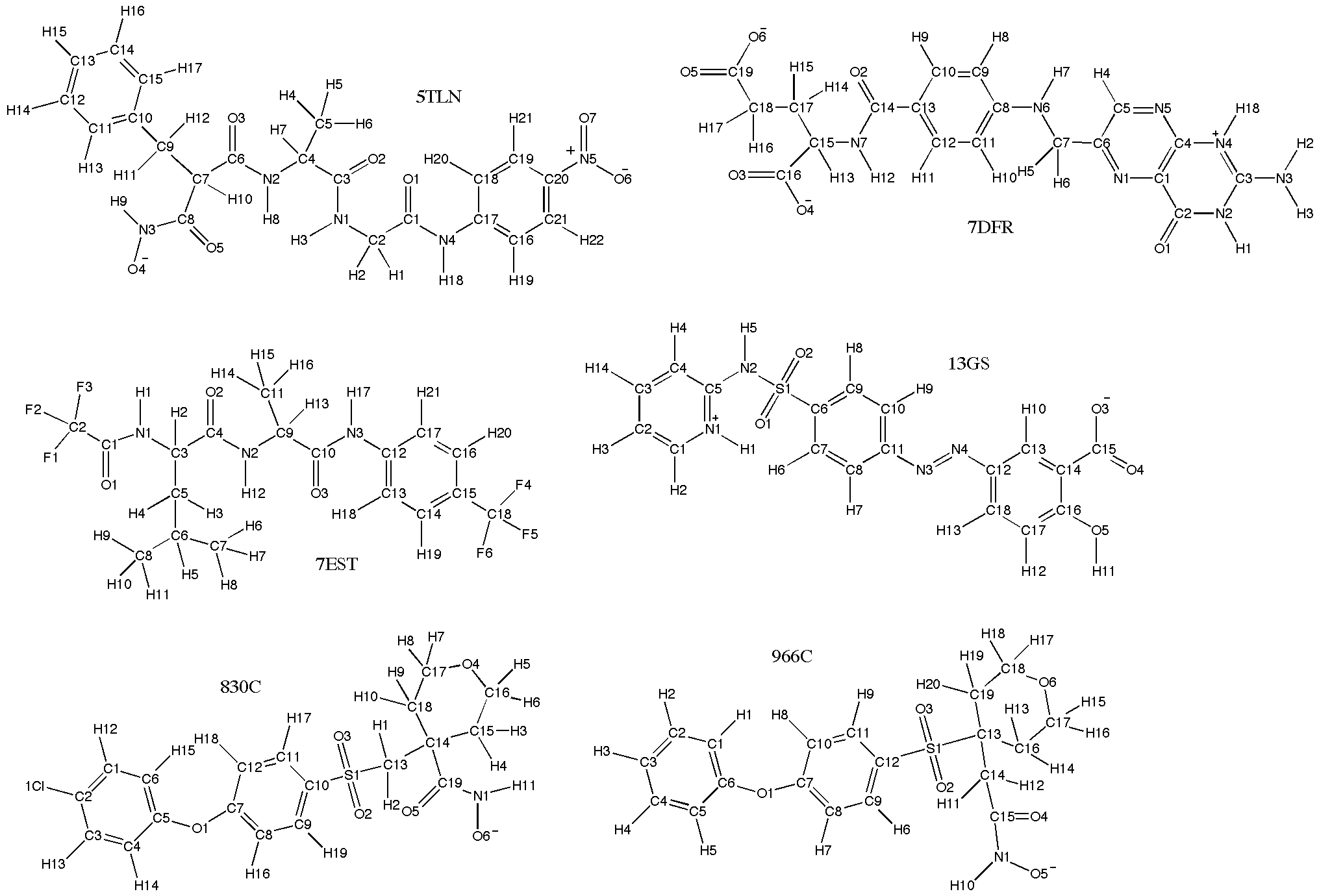

Supplement: Figure S13 — Definitions of atom labels of the 100 protein-bound ligands (Part VII). (2.80 MB TIF) [file pone.0000820.s018.tif]
